# An end-to-end hybrid deep-learning approach for single-shot wavefront sensing and correction

**DOI:** 10.1038/s41467-026-72364-1

**Published:** 2026-05-12

**Authors:** Sina Moayed Baharlou, Muhammad Waleed Khalid, Guli Gulinihali, Jeongho Ha, Liyi Hsu, Samantha C. Lewis, Lei Tian, Yeshaiahu Fainman, Alexander V. Sergienko, Abdoulaye Ndao

**Affiliations:** 1 Department of Electrical and Computer Engineering, University of California, San Diego, La Jolla, CA USA; 2 Department of Electrical and Computer Engineering & Photonics Center, Boston University, Boston, MA USA; 3 Department of Molecular and Cell Biology, University of California, Berkeley, CA USA

**Keywords:** Sub-wavelength optics, Nanophotonics and plasmonics

## Abstract

Optical aberrations fundamentally limit the performance of optical systems across scales, from microscopy and astronomy to quantum imaging and ubiquitous technologies such as smartphone cameras, autonomous vehicles, and optical communication networks. Despite sustained progress in wavefront sensing and correction, existing approaches typically require multiple intensity measurements and iterative phase retrieval algorithms and are often restricted to narrowband operation and simple beam profiles. Their performance further degrades when aberrations are weak or obscured by noise. Here we report a single-shot wavefront sensing and correction paradigm that overcomes the limitations of existing approaches. Central to the approach is the integration of a learned optical phase mask that acts as a physical encoder and is jointly optimized with a neural-network decoder. This encoding removes intrinsic phase ambiguities and significantly enhances sensitivity to weak aberrations. By leveraging this hybrid deep-learning architecture, our method directly and unambiguously retrieves Zernike-based phase distortions from a single focal-plane intensity image, eliminating the need for defocus measurements, or iterative phase reconstruction. The framework is intrinsically broadband, exhibits strong robustness to noise, and generalizes across a diverse class of structured light fields. Together, these capabilities establish a scalable and practical foundation for real-time aberration correction in next-generation optical and photonic systems.

## Abstract (web summary)

A hybrid deep-learning framework is presented that detects and corrects optical aberrations from a single intensity image, using a learned phase mask and neural-based decoder for fast, accurate correction across broadband and structured light fields.

## Introduction

In the early 1600s, Galileo Galilei frustratedly observed that the celestial bodies seen through his telescope were fringed with color and haloed with blur^1^. Since then, optical aberration has continued to fascinate the scientific world. Once dismissed as optical imperfections, aberrations have since catalyzed a vibrant field of research; spanning adaptive optics^2,3^, computational wavefront correction^4^, and engineered materials; where their control is not only fundamental to advancing microscopy^5^, astronomy^6^, and quantum imaging but also dictate how light is captured, focused, and perceived.

Despite sustained advances in waveform shaping over decades^7–14^, optical aberrations continue to impose fundamental limits on optical design performance, with all existing correction strategies constrained by inherent trade-offs in accuracy, speed, generality, and physical scalability. Direct wavefront sensing methods^15^, such as Shack–Hartmann^16^ sensors enable real-time phase recovery, but are hindered by non-common path errors, limited spatial resolution, and the bulky, cost-intensive nature of the hardware^17^. Furthermore, Shack-Hartmann sensors rely on individual pixel values to calculate the local slope of the wavefront, this makes their performance sensitive to intensity fluctuations under low-light conditions. These limitations consequently hinder the scalability of wavefront sensors and their integration into compact platforms. Indirect computational techniques, including iterative phase retrieval algorithms like the Gerchberg-Saxton method^18,19^, and optimization-based^20^ methods eliminate the need for dedicated wavefront sensors, but are inherently slow, susceptible to convergence errors, and fundamentally reliant on multiple intensity measurements due to phase ambiguity. Machine learning^21–25^ approaches have emerged as powerful data-driven alternatives, offering potential gains in speed and generalization; however, they remain fundamentally limited by the phase ambiguity. Such phase ambiguity arises from a fundamental spatial symmetry in coherent imaging systems, whereby sign-negated-reversed phase distributions, $\varphi (x,y)$ and $-\varphi (-x,-y)$, produce identical intensity patterns at the image plane. Overcoming this issue requires capturing multiple intensity images, including one at the focal plane and others at several defocused planes; a technique known as phase diversity^26–28^, which adds considerable time and resource demands to the correction process.

Recently, wavefront-sensing methods rely on nontraditional optical encoders, such as photonic lanterns, optical neural networks, fixed phase masks, or diffusers, often combined with computational reconstruction or data-driven inference. Photonic lanterns^10^ have been proposed as wavefront sensors by placing them before the focal plane and reconstructing the wavefront from measured single-mode output intensities using deep learning. Emerging diffractive neural networks^11,29–31^ have introduced promising hardware-encoded solutions for complex-field imaging. These include methods that map phase objects to intensity images^30^ and an approach using diffractive neural networks to detect Zernike-based aberrations^11^. Photonic lanterns and diffractive neural networks, however, are constrained by computational limitations, narrowband operation, fabrication complexity, and limited compatibility with broadband, non-paraxial, or structured fields. Closely related to diffractive neural networks are works on differentiable optics^32,33^ and PSF engineering^34^, which aim to improve incoherent imaging. In these works, a fixed or optimized phase mask is introduced into the optical path and followed by computational reconstruction to enable lensless imaging and improve image quality. Coherent modulation imaging^35^ employs a phase mask in the optical path to enhance both coherent and incoherent imaging. It often utilizes fixed, non-optimized patterns with iterative reconstruction, which may involve multiple masks^36^. Diffuser-based approaches have enabled single-shot estimation of multiple isoplanatic wavefronts^37^, recovery of spatially varying aberrations via tilted illumination, and compact lenslet-free sensing that requires strict calibration and fixed geometry^38,39^. These works highlight the promise of compact, low-cost encoders; however, they lack encoder phase optimization, and computational efficiency, and are less robust to noise due to beam scattering.

Existing methods have an additional limitation, namely their reliance on idealized Gaussian beams, an assumption that restricts their applicability to complex structured optical fields, including vortex and higher-order modes^40–44^. Structured light fields, such as Hermite-Gaussian (HG) and Laguerre-Gaussian (LG) beams, are integral to a wide range of applications. HG beams are used in mode-division multiplexing and cavity diagnostics for precision interferometry, where their Cartesian nodal structure makes them particularly sensitive to optical aberrations. LG beams play a key role in stimulated emission depletion microscopy and optical tweezers, where their vortex phase structure enables super-resolution and the transfer of optical angular momentum. These examples highlight the need to assess wavefront sensing and correction techniques beyond simple Gaussian beams to ensure relevance to practical optical systems.

Here, we demonstrate a single-shot approach to wavefront sensing and correction that overcomes the aforementioned limitations of conventional techniques. Using a hybrid deep-learning architecture, the method directly reconstructs phase distortions in Zernike space from a single focal-plane intensity image, without requiring defocus measurements, iterative phase retrieval, or prior assumptions about the input beam profile. Beyond its conceptual simplicity, the approach is intrinsically broadband and demonstrates strong robustness to detector noise, low-signal conditions, and saturation effects. Importantly, it enhances the sensitivity of intensity-only measurements to weak aberrations that are otherwise difficult to detect. The framework further generalizes across a wide range of structured optical fields, including Orbital-Angular-Momentum (OAM), Hermite-Gaussian, and Laguerre-Gaussian modes. This approach not only collapses the wavefront sensing pipeline into a single computational step but also establishes a scalable foundation for real-time aberration correction in next-generation optical systems.

## Results

**Fig. 1:**
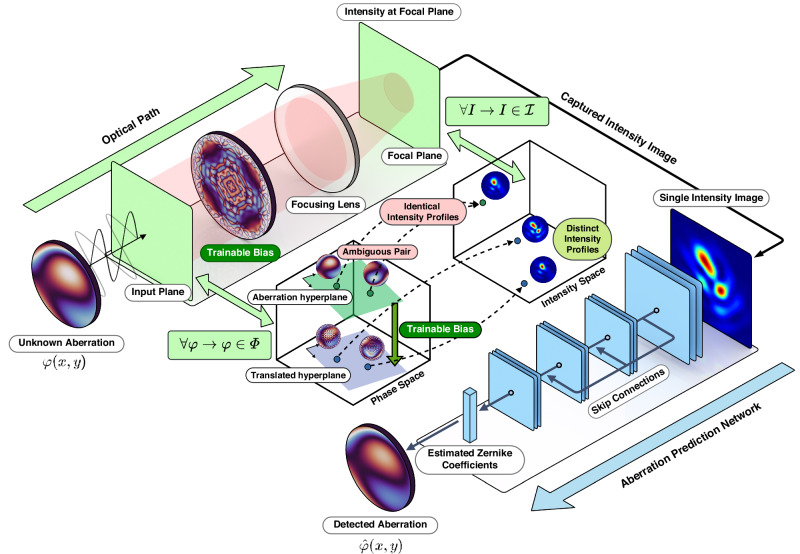
Schematic of the proposed framework to detect optical aberrations from single-intensity images of various beam types. The framework consists of two main components: an optical and a deep learning module. The optical module incorporates a trainable bias, implemented as a phase plate optimized to resolve focal plane ambiguities and enhance sensitivity to weak aberrations. This module receives a beam with unknown aberration as input and operates directly on various beam types, including Gaussian and vortex beams with different topological charges, as well as more complex structured light such as Laguerre-Gaussian (LG) and Hermite-Gaussian (HG) beams. The deep learning module features an aberration prediction network (APN), a residual network trained to infer aberrations in Zernike-space from single intensity measurements. The bias is jointly optimized with the APN and transforms the aberration subspace, which is initially affected by ambiguity and low sensitivity, into a new space where each aberration produces a unique, distinguishable intensity pattern. This concept is depicted using two hyperplanes (original and translated subspaces) and representative points corresponding to aberrations and their associated intensity profiles.

To tackle the challenge of aberration detection from single image measurement, we introduce a hybrid optical-machine-learning framework that leverages deep neural networks (DNNs)^45^ for Zernike-based phase error characterization. The architecture comprises a compact optical front-end and a deep neural backend (Fig. 1), co-designed and jointly optimized to recover wavefront distortions across a wide class of optical fields. Our approach features trainable phase bias, introduced via a spatial light modulator (SLM) or a metasurface, that is designed to eliminate the intrinsic phase ambiguity of the incident wavefront. This engineered phase plate enhances the sensitivity of focal-plane intensity measurements to phase aberrations, enabling robust and highly discriminative aberration detection from a single image. The deep learning module performs two key functions: (i) it learns the optimal phase modulation pattern for the bias through backpropagation^46^ and (ii) it infers the underlying aberrations from the resulting intensity image.

This module includes the Aberration Prediction Network (APN), a deep residual^47^ neural network trained to regress a fixed number of Zernike coefficients that parameterize the wavefront distortion.

In our approach, the optical and learning components operate synergistically: the APN guides the bias modulation to reshape the optical response space, transforming a degenerate and ambiguity-prone aberration subspace into one where each aberration yields a unique and discriminative intensity signature. Conceptually, this transformation can be visualized as a mapping from one aberration manifold to another, where different wavefront errors give rise to unique and readily identifiable intensity profiles. This transformation establishes a one-to-one correspondence, enabling the APN to learn the mapping effectively. Moreover, the framework accurately recovers aberrations not only from standard Gaussian beam profiles but also from a broad class of structured beams, including vortex beams with varying topological charges, and more complex structured profiles such as LG and HG beams extending the applicability of DNN-based inference to a wider range of optical systems.

To lift the inherent ambiguity, we introduce a mathematical model that reveals a predominant requirement: the bias must have a non-zero even symmetrical component that lies outside the subspace spanned by the finite set of even Zernike polynomials producible by the system (Supplementary Information Section 2). This stems from the fact that focal plane intensity (Eq. 1) remains invariant under sign changes of the even symmetric phase components, rendering them intrinsically ambiguous:

1 $${{|}{{{\rm{F}}}}\{{{{\rm{A}}}}{e \, }^{i\left({{{{\rm{\varphi }}}}}_{{{{\rm{o}}}}}{\pm }\,{{{{\rm{\varphi }}}}}_{{{{\rm{e}}}}}\right)}\}|}^{2}{=}{{|}{{{\rm{X}}}}{\pm }i{{{\rm{Y}}}}{|}}^{2}{=}{{{{\rm{X}}}}}^{2}+{{{{\rm{Y}}}}}^{2}$$

Where ${{{\rm{F}}}}$ is the Fourier transform, ${{{\bf{A}}}}$ is the amplitude, ${{{{\boldsymbol{\varphi }}}}}_{{{{\rm{o}}}}}$, ${{{{\boldsymbol{\varphi }}}}}_{{{{\rm{e}}}}}$ are the odd and even components of the phase, respectively, and ${{{\bf{X}}}}$ and ${{{\bf{Y}}}}$ are the real and imaginary parts of the field at the focal plane, respectively. Introducing a phase bias ${{{{\boldsymbol{\varphi }}}}}_{{{{\rm{b}}}}}$ removes this ambiguity by ensuring ${{{{\boldsymbol{\varphi }}}}}_{{{{\rm{e}}}}}{{{\boldsymbol{+}}}}{{{{\boldsymbol{\varphi }}}}}_{{{{\rm{b}}}}}$ and ${-{{{\boldsymbol{\varphi }}}}}_{{{{\rm{e}}}}}+{{{{\boldsymbol{\varphi }}}}}_{{{{\rm{b}}}}}$ yield distinct intensity patterns. Since aberrations typically lie on a low-dimensional manifold within a higher-dimensional phase space, the bias must be chosen in a higher dimension beyond this manifold to effectively resolve the ambiguity.

Furthermore, the framework is trained in an end-to-end fashion to provide real-time detection and in situ correction information. The system is robust to fabrication imperfections, shot noise and camera noise and can operate in the entire visible range (Supplementary Information Sections 3 and 7). To train and validate our framework under a broad range of conditions, we constructed a comprehensive suite of synthetic datasets (Supplementary Information Section 4) comprising manually aberrated beams. The dataset spans ten distinct spatial beam profiles, including Gaussian, vortex, and higher-order structured beams such as LG and HG (Supplementary Table 1). Aberrations were encoded using either the first nine, fourteen, or twenty-seven Zernike polynomials, with coefficients drawn from diverse statistical priors, including uniform distributions, Kolmogorov turbulence models^48^, and sparse one-hot vectors.

To determine the optimal phase distribution for the trainable bias, we trained the framework using a dataset with nine polynomials in which Zernike coefficients were uniformly sampled within the range ( − 1, 1), ensuring uniform coverage of the aberration space and preventing preferential weighting of specific polynomial modes. We applied several symmetrical constraints and presented the results in Section 5 of the Supplementary Information. Performance was quantitatively assessed on a held-out test set by computing the root-mean-squared error (RMSE) between the predicted and ground-truth aberration phases, yielding an average RMS error of $1.9\times 1{0}^{-2}\,\pi$ radians. The convergence behavior of the learning process is shown in Supplementary Fig. 5, where training and validation curves indicate stable optimization and consistent generalization (Supplementary Information Section 5).

**Fig. 2:**
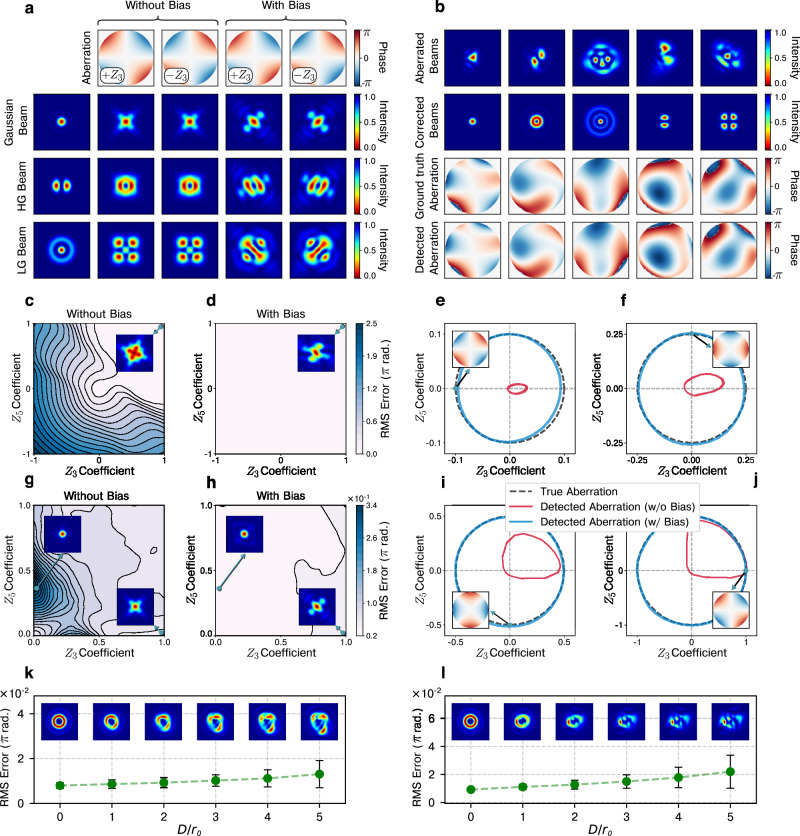
Numerical validation of the aberration detection and correction using the proposed framework. **a** Simulated intensity responses of various beams subjected to astigmatic aberrations of opposite signs (Zernike mode Z_3_), shown before and after bias interaction. In the absence of bias, the beams yield indistinguishable intensity profiles; introducing the trained bias enables the generation of distinct, classifiable patterns. **b** Examples of aberration detection and correction using samples generated from the Kolmogorov turbulence model (D/r_0_=4, Z_14_). The first row shows Gaussian, OAM (${{{\mathcal{l}}}}=1$), LG_1,1_, HG_0,1_and HG_1,1_ beams with applied aberrations. The second row shows the corresponding corrected beams. The third and fourth rows display the ground truth aberrations and those predicted by the APN, respectively. **c**, **d**, **g**, **h** Prediction error of the system (Gaussian beam) evaluated over 2D cross-sections of the Z_3_–Z_5_ aberration space, before and after applying the bias interaction, **c** and **d** are computed over the interval ( − 1, 1) without and with bias, respectively; **g** and **h** are computed over the interval (0, 1), also without and with bias. Insets show the corresponding intensity profiles of the aberrated beams at sel**e**cted po**i**nts. **e,** **f,** **i,** **j** Prediction accuracy for astigmatism coefficient pairs (Z_3_–Z_5_) sampled along circles of varying radii, with and without bias, using a Gaussian beam. Coefficient magnitudes vary continuously between positive and negative values. Dashed lines indicate ground truth, solid red lines show predictions without bias (baseline), and solid blue lines show predictions with bias. Insets show the corresponding normalized aberration profiles. **k**, **l** RMS error of the framework evaluated on the Kolmogorov dataset. Aberrations are represented using the first nine (**k**) and first fourteen (**l**) Zernike polynomials, with varying strength levels. Inset: intensity profile of an OAM beam with ${{{\mathcal{l}}}}=2$ under a random aberration of specified strength. Error bars represent the standard deviation (SD).

Importantly, the learned bias demonstrates promising behavior in resolving the inherent sign ambiguity associated with angularly even Zernike modes, enabling accurate polarity detection by the APN. This capability is illustrated in Fig. 2a, which compares the intensity responses of structured beams subjected to astigmatic aberrations of opposite sign, both before and after interaction with the phase bias. The pre-bias intensity profiles are nearly indistinguishable, whereas post-bias images exhibit clear, differentiable structure, thus demonstrating that the bias renders otherwise degenerate aberrations optically separable. Figure 2b illustrates several examples of numerically performed aberration detection and correction processes. To further validate the robustness and generality of the bias, we designed several extensive benchmark experiments and reported the results in the Supplementary Information Section 6. These include comparisons of the trained bias against fixed phase patterns (Section 6.1.1), evaluations of different bias-aberration interaction areas (Section 6.1.2), investigations into ambiguity removal in higher-order modes (Section 6.1.3), low-light assessments (Section 6.5), out-of-domain evaluations (Section 6.6), and training and testing on a dataset featuring aberrations expressed using the first twenty-seven Zernike polynomials (Section 6.7).

To further demonstrate the effectiveness of the trained bias in recovering the sign of Zernike-based aberrations and enhancing the sensitivity of intensity measurements, we compute the RMS error over a grid of evaluation points spanning the astigmatism cross-section (Z_3_–Z_5_)and compare our framework to a baseline network trained without the bias (Fig. 2c, d). The baseline fails to predict the sign due to ambiguity, whereas the network with a trained bias successfully resolves it. Figure 2g, h show evaluations on positive coefficients, excluding the sign ambiguity effects. The baseline performs poorly in regions where intensity is insensitive to coefficient changes (Fig. 2g), while the network with trained bias achieves consistent, superior performance across the entire area (Fig. 2h).

To rigorously assess the framework’s accuracy, particularly in the regime of weak aberrations, we designed a controlled experiment using astigmatism coefficient pairs (Z_3_–Z_5_) sampled along circles of varying radii. This configuration enables simultaneous testing of sign resolution and sensitivity to low-magnitude inputs. For direct comparison, ground-truth coefficients, alongside predictions from our model and baseline, are plotted. As shown in Fig. 2e, f, i, j the baseline consistently fails to recover the correct sign and magnitude near the origin. In contrast, our framework produces smooth, accurate predictions across all radii, thus highlighting its robustness to low-magnitude aberrations and validating the effectiveness of the learned bias. Further results are provided in Supplementary Information Section 6.2.

Figure 2k and l shows the numerical evaluations of the framework on the turbulence model dataset, with aberrations generated using the first nine (Fig. 2k) and first fourteen (Fig. 2l) Zernike polynomials. Each test set, consisting of 5,000 samples, was evaluated across six levels of aberration strength, scaled by $D/{r}_{0}$, where $D$ is the system diameter and ${r}_{0}$ is the Fried parameter, ranging from 0 (no aberration) to 5. The average RMS error across all aberration strengths is $1.0\times 1{0}^{-2}\,\pi$ radians for Z_9_ and $1.4\times 1{0}^{-2}\,\pi$ radians for Z_14_. Kolmogorov datasets show lower error than the uniform dataset due to its slightly lower aberration strength. Further evaluations on the turbulence and one-hot datasets are provided in Supplementary Information Section 6.3 and 6.4.

To experimentally validate the proposed framework, we begin by generating controlled wavefront aberrations using a liquid crystal on silicon spatial light modulator (LCOS-SLM), applied across a range of beam types, including Gaussian, vortex beams of varying topological charge, and higher-order structured modes. To assess the impact of these aberrations, we record the resulting focal plane intensity profiles after propagation through a lens. To generate well-defined input beams, we employed a broadband supercontinuum laser source (SuperK FIANIUM) with an acousto-optic tunable filter, enabling precise selection of operating wavelengths. The fiber-coupled output ensures a high-purity Gaussian spatial mode, providing a stable and reproducible baseline for all measurements. To demonstrate the broadband achromaticity of our devices, we selected three different wavelengths, 500 nm, 600 nm, and 700 nm, which are widely used in bioimaging applications and performed experiments to verify their performance and robustness across varying optical conditions (Supplementary Information Section 7). To expand the evaluation, we generated HG beams using amplitude modulation and OAM beams using phase-only modulation, following the standard mathematical models described in ref. ^49^.

To introduce and experimentally characterize optical aberrations, we adopted the same protocol used in our numerical simulations (Supplementary Fig. 19), thereby allowing direct comparison between simulated and physical outcomes. Aberrations were generated using the first nine Zernike polynomials, drawn from two previously generated datasets. The first, based on a Kolmogorov turbulence model with $D/{r}_{0}\,=4$, captures stochastic aberrations representative of atmospheric and thermally induced distortions. The second, constructed using uniformly sampled coefficients, provides a controlled environment for probing the system’s response to systematically varied phase errors. We selected 10 percent of the samples from the Kolmogorov and Uniform test sets. These aberration coefficients were applied to the SLM, with adjustments based on beam type, bias configuration, and wavelength. The camera then captured and stored the resulting intensity images. The APN processed the captured images and predicted the corresponding aberrations, which were used to correct the distorted beams.

We introduced a noise regularization scheme during training to enhance the robustness of the APN to real-world imperfections including fabrication tolerances, SLM nonidealities, photon-shot and sensor-induced noise. Specifically, two complementary regularizers were employed to perturb both the input phase maps and the corresponding intensity images. Phase perturbations were sampled from a zero-mean Gaussian distribution with a standard deviation of ${{{\rm{\pi }}}}/8$ radians, emulating high-frequency fabrication defects and SLM calibration errors. Concurrently, intensity perturbations incorporated a range of degradations commonly encountered in imaging systems, including random cropping, low-intensity thresholding, overexposure, additive Gaussian noise, and salt-and-pepper noise. This regularization approach helps the network focus on principal features in the intensity patterns that remain stable despite noise and variations. As a result, the network becomes much more reliable when faced with real experimental fluctuations. The benefits of this noise-aware training are clearly shown through comparison of cases with and without the regularization, as detailed in Supplementary Information Section 7.

**Fig. 3:**
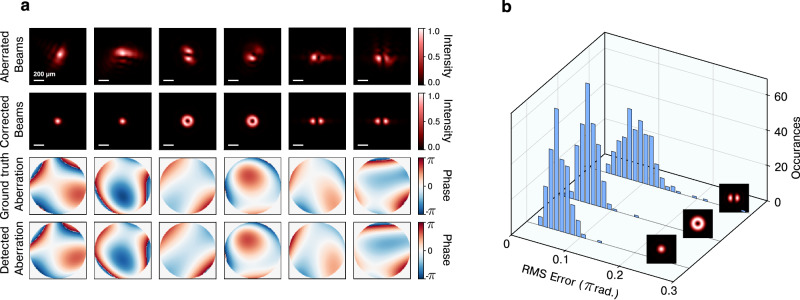
Experimental validation of the framework using the Spatial Light Modulator. **a** Examples of aberration detection and correction using samples generated from the Kolmogorov turbulence model (D/r_0_ = 4, Z_9_) with an SLM performed at $\lambda=700$ nm. The first row illustrates different aberrations applied to Gaussian, OAM (${{{\mathcal{l}}}}=1$), and HG_1,0_ beams, with two samples each. The second row shows the corresponding corrected beams. The third and fourth rows display the ground truth aberrations and those predicted by the APN, respectively. Scale bars represent 200 μm. **b** Distribution of RMS prediction error on the Kolmogorov dataset (D/r_0_ = 4, Z_9_) for three individual beams: Gaussian, OAM (${{{\mathcal{l}}}}=1$), and HG_1,0_. Errors are computed over 250 random aberration instances per beam.

Employing the noise regularizers, we observed an average RMS error of $8.3\times 1{0}^{-2}\,\pi$ radians for the Uniform dataset and $6.9\times 1{0}^{-2}\,\pi$ radians for the Kolmogorov dataset. Figure 3a shows examples of the detection and correction process, while Fig. 3b presents the error distributions for each beam type. Additional results at other wavelengths are provided in Supplementary Section 7.3.

To solve the bottleneck of aberration correction where bulk optics fall short, modern imaging demands optical elements that are thin, lightweight, and compact, yet capable of maintaining diffraction-limited performance. Metasurfaces meet this need by providing precise wavefront control in ultrathin platforms, thereby enabling aberration-free imaging in astronomy, bioimaging, and in vivo applications. We propose replacing the SLM with a metasurface phase plate, offering a compact, passive solution for precise wavefront control.

To introduce the phase modulation profile required for implementing the trained bias, we employ a Pancharatnam-Berry (PB) metasurface composed of spatially rotated, subwavelength anisotropic elements. In this geometric-phase-based scheme, a full $2\pi$ phase shift is achieved solely through the in-plane rotation of structurally identical meta-atoms. When illuminated with right-handed circularly polarized light, the incident wave decomposes along the meta-atom’s principal axes, each associated with complex transmission coefficients ${t}_{0}$ and ${t}_{{{{\rm{e}}}}}$. The transmitted electric field, ${{{{\bf{E}}}}}_{{{{\bf{t}}}}}$, is therefore given by^50^:

2 $${{{{\rm{E}}}}}_{{{{\rm{t}}}}}=\frac{{t}_{0}+{t}_{{{{\rm{e}}}}}}{2}\widehat{{{{{\rm{e}}}}}_{{{{\rm{R}}}}}}+\frac{{t}_{0}-{t}_{{{{\rm{e}}}}}}{2}\exp (i2\theta )\widehat{{{{{\rm{e}}}}}_{{{{\rm{L}}}}}}$$

The first term in Eq. 2 describes the component of the transmitted field that retains the incident helicity and does not acquire any phase shift. The second term corresponds to the cross-polarized component, i.e., the transmitted wave with opposite helicity which carries the Pancharatnam-Berry (PB) phase ($2\theta$), where $\theta$ defines the rotation angle of the meta-element. In theory, a PB metasurface can reach 100% conversion efficiency if the transmitted light is entirely cross-polarized. However, for proof-of-concept demonstrations and imaging applications, such an ideal conversion is not required. A clear image can be obtained by placing a circular polarization filter to suppress the residual co-polarized light as long as the cross-polarized component exceeds the detection threshold (typically >10%). In our implementation, the metasurface is composed of TiO_2_ elliptical nanostructures (height: 250 nm; semi-major axis: 160 nm; semi-minor axis: 60 nm), arranged with a 400 nm periodicity on a SiO_2_ substrate. The simulated polarization conversion efficiency across the design wavelength range is presented in Supplementary Fig. 42.

**Fig. 4:**
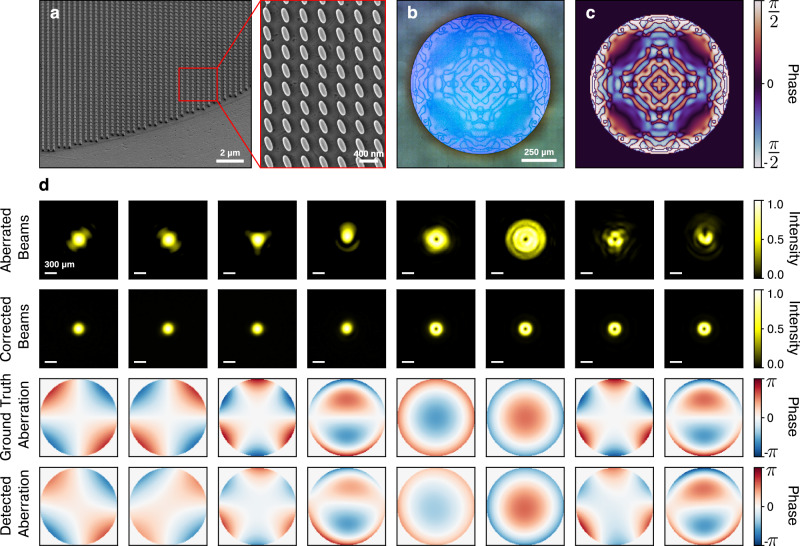
Experimental validation of the framework using the Fabricated Metasurface. **a** Scanning electron microscope (SEM) image of the fabricated TiO_2_ metasurface. The left subfigure shows a tilted view of the uniform nanopillar array on the BK7 substrate. The right subfigure (zoom) shows a magnified top view of the metasurface, revealing elliptical TiO_2_ nanopillars with subwavelength dimensions and spacing. **b** Top-view brightfield optical microscope image of the fabricated metasurface, illustrating the phase pattern and showing close alignment with the desired one. **c** Optimal bias phase profile determined during the training process. **d** Experimental demonstration of aberration detection and correction using the fabricated metasurface at $\lambda=\,600$ nm. The first row shows the intensity profile of aberrated beams, where various Zernike bases are applied to Gaussian and OAM (${{{\mathcal{l}}}}=1$) beams (four samples each). The second row shows the resulting beams after aberration correction. The third row displays the ground truth aberrations used to generate aberrated beams, and the last row presents the aberrations detected by the APN. Scale bars represent 300 μm.

For this experiment, we positioned the metasurface in the optical path after the focusing lens, conducting the tests at a wavelength of 600 nm. We selected manual aberrations using individual Zernike bases to evaluate the framework, focusing specifically on ambiguity removal and the detection of aberrations using the APN in a more complex setup where fabrication imperfections play a significant role. We conducted experiments using Gaussian and OAM (${{{\mathcal{l}}}}=1$) beams. In this setup, the SLM introduced the aberrations, while the metasurface induced the necessary phase shifts. The intensity was captured at the focal plane, and the APN recovered the aberration. The corrected beam was obtained by uploading the conjugate of the estimated aberration onto the SLM. We utilized the first nine Zernike basis functions, including both negative and positive values. The average RMS error obtained from this experiment is $0.104\,\pi$ radians. Samples of this process are illustrated in Fig. 4. A comparison of the intensity responses to Zernike Bases obtained from the simulation, and metasurface can be found in the Supplementary Fig. 24. The results demonstrate the robust and consistent performance of the proposed framework in eliminating ambiguity, recovering, and correcting the beam with the use of the fabricated metasurface.

## Discussion

In this work, we introduce a hybrid end-to-end deep-learning framework for single-shot wavefront sensing and correction based on a single intensity measurement. Central to the approach is the integration of a learned optical phase mask that acts as a physical encoder and is jointly optimized with a neural-network decoder. This encoding removes intrinsic phase ambiguities and significantly enhances sensitivity to weak aberrations. The decoder estimates phase distortions in Zernike space directly from the encoded intensity images, enabling accurate aberration retrieval without iterative optimization. The framework is intrinsically broadband and applicable to a wide range of structured light fields.

The method is validated through comprehensive numerical simulations and experimental demonstrations using both a spatial light modulator and a fabricated metasurface. Importantly, the framework can be extended to handle dynamic and spatially varying aberrations, which are prevalent in biological imaging and atmospheric propagation. Because the approach does not rely on iterative correction loops, aberration prediction occurs in a single inference step, enabling rapid response to time-varying distortions. The achievable correction speed is ultimately limited by the refresh rate of the wavefront-modulating element, such as the SLM, rather than by computational overhead.

For spatially varying aberrations, the framework can be implemented by calibrating local aberration coefficients across the field of view and applying sequential corrections in point-scanning systems. In wide-field configurations, the recorded image can be transformed into the appropriate local reference frame corresponding to the spatially varying aberration profile, effectively rendering the aberrations locally shift-invariant and enabling accurate compensation.

An important consideration in aberration prediction is representational capacity. Our approach adopts an indirect strategy in which the network predicts a fixed set of Zernike coefficients. While extending the output space typically requires retraining, this does not constitute a practical limitation: retraining is computationally efficient, requiring approximately 1.5 hours for 100 epochs with nine coefficients and substantially less time for reduced training schedules. Comparable training times were observed for models predicting higher-order coefficients. Alternative strategies, such as transfer learning^51^, may further reduce training costs, although full retraining consistently yielded the highest accuracy.

We also evaluated a direct phase-reconstruction approach based on a U-Net^52^ architecture, in which the network predicts the phase profile directly rather than estimating Zernike coefficients. This strategy did not provide performance improvements, reflecting the fact that the training data are defined in the Zernike domain and therefore constrain the network’s expressive capacity. Despite this, the indirect model remains robust when presented with aberrations outside the training distribution. Tests on out-of-domain samples including atmospheric turbulence (not generated from Zernike polynomials), smooth random phase profiles, and higher-order aberrations demonstrate that the network reliably recovers the dominant low-order components without collapsing, indicating that it has learned meaningful features rather than overfitting to the training set (Supplementary Information Section 6.6).

To isolate the contribution of the learned optical bias, we assessed its impact on classical intensity-based reconstruction methods. When incorporated into a gradient-descent-based algorithm, the trained bias significantly improved reconstruction accuracy, reducing the root-mean-square error by 38.5%, respectively. This result demonstrates that the learned physical encoding is transferable across reconstruction paradigms and not limited to neural-network-based decoders (Supplementary Information Section 7.3.3).

Finally, we benchmarked the proposed framework against a numerically simulated Shack-Hartmann wavefront sensor. Unlike Shack-Hartmann sensors, whose resolution is constrained by microlens pitch and whose performance degrades under vibration or low illumination, our approach retrieves a continuous wavefront directly from a single focal-plane intensity image and remains effective under low-light and noisy conditions (Supplementary Information Section 10). Together, these results position the proposed framework as a scalable and versatile alternative to conventional wavefront sensing techniques, with clear advantages for real-time, broadband, and structured-light optical systems.

In conclusion, we present a framework for aberration sensing and correction that operates using single focal-plane intensity image. This method demonstrates accurate recovery of system aberrations in Zernike space across a wide range of illumination powers, numerical apertures, and optical configurations, without requiring additional training data or specialized calibration beyond standard SLM characterization. By incorporating the aberration-correction module into the imaging path and sampling a limited fraction of the focal-plane intensity image, wavefront distortions can be inferred and pre-compensated with low latency, enabling improved aberration control at the specimen. This capability points toward future developments in applications that benefit from precise wavefront control, including structured-beam microscopy, high-resolution imaging, and controlled beam-matter interactions.

## Methods

### Numerical simulations

The optical setup is modeled numerically by representing the light field at the entrance pupil of a focusing lens, which is then propagated and focused at the focal plane. The Fresnel diffraction integral is utilized to account for light diffraction. We implemented a differentiable version of the Bluestein method^53^ to model this diffraction integral. This approach provides gradients that aid in determining the optical bias phase distribution and allows for flexibility in defining regions of interest and selecting arbitrary sampling rates. Phase aberrations are modeled using the first nine, fourteen, or twenty-seven polynomials according to ANSI standard indexing. The aberration coefficients are sampled from three different distributions: a uniform dataset used for training and a Kolmogorov and one-hot datasets for testing. To train the bias the uniform dataset with nine coefficients has been used. This dataset consists of 100,000 aberration samples, with 90% allocated for training and 10% reserved for validation and testing (5,000 samples each). Each sample includes a randomly selected beam type and a randomly sampled aberration. The Bluestein method operates with a fixed wavelength of 400 nm to generate the training data. Since we capture the intensity at the focal plane, changes in wavelength only change the geometrical scale of the intensity image ($\lambda /2{NA}$). As a result, capturing the intensity shape with just one wavelength is sufficient. To ensure that the model remains scale invariant in the entire bandwidth, the APN is trained with different scale sizes that mimic variations in wavelength within the bandwidth (Supplementary Section 7.2). The APN is a modified residual network whose architecture has been optimized using hyperparameter optimization method and is similar to ResNet34^47^. To obtain optimal biases and network parameters, the framework is trained for 100 epochs, minimizing the mean-squared-error (MSE) between the predicted and ground truth Zernike coefficients. The Adam optimizer is used with a batch size of 64 and a learning rate of 0.004. The accuracy is evaluated on the test set using root-mean-squared error (RMSE) in both the Zernike and phase domains. The RMSE in the phase domain is computed over the full rectangular computational grid and applied consistently across all models and experiments. The entire framework is implemented in PyTorch^54^ and runs on the Linux operating system with a RTX 3080 TI GPU. Training 100,000 images takes one and half hours.

### SLM calibration and aberration control

To ensure consistency between simulation and experiment, the SLM was first corrected using the manufacturer-provided internal phase calibration. The optical path was kept intentionally minimal to reduce system-induced aberrations. Any residual distortions were then compensated using our model trained on theoretical data, with beam-quality agreement to the ideal profile serving as the figure of merit. All optical components used were selected and coated for the operating wavelength to avoid chromatic effects. We validated the phase response of the SLM by applying individual Zernike modes (Z_1_-Z_9_) and confirming that (i) our sign and amplitude conventions matched those used in simulation and (ii) the applied Zernike amplitudes scaled linearly when written to the SLM. The beam diameter was matched to the mapped Zernike phase profile using digital apertures to remove unused SLM regions. A blazed grating was added to spatially separate the modulated beam from residual unmodulated components arising from finite polarizer extinction. Finally, the image plane was verified at the focal plane of the collection lens, where the ambiguous Zernike pair served as a diagnostic since both signs must yield identical focal-plane intensity patterns.

### Metasurface fabrication

To fabricate the designed metasurface, we use a top-down nanofabrication process. The fabrication uses three main steps. The first step consists of patterning the polymethyl methacrylate (PMMA) resist using electron beam lithography (EBL) which is subsequently developed in solution to remove the exposed PMMA. The pattern is the inverse of our final metasurfaces. In the second step, the exposed sample is transferred to an atomic layer deposition (ALD). The ALD process deposits TiO_2_ thickness so that all features are filled. The third step consists of removing the residual TiO_2_ film that coats the top surface of the resist using reactive-ion-etching. After removing PMMA, the TiO_2_ metasurfaces were obtained. As shown in the scanning electron microscope (SEM) image of the metasurface in Fig. 4a, b, the structure exhibits high fidelity, confirming the quality of fabrication.

## Supplementary information

Supplementary Information

Transparent Peer Review file

## Data Availability

The datasets generated in this study are under restricted access due to an ongoing patent. Access can be obtained from the corresponding author upon request to ensure reproducibility. The datasets will be publicly released upon patent publication.

## Code availability

The code developed in this study is under restricted access due to an ongoing patent. Access can be obtained from the corresponding author upon request to ensure reproducibility. The code will be publicly released upon patent publication.
