# Supplementary material for: An end-to-end hybrid deep-learning approach for single-shot wavefront sensing and correction
Source: Nat Commun. 2026 May 12;17:6340. doi: 10.1038/s41467-026-72364-1 (PMC13377082; doi:10.1038/s41467-026-72364-1)
Supplement: Supplementary file 1 — Supplementary Information [file 41467_2026_72364_MOESM1_ESM.pdf]

Supplementary Information for An end-to-end hybrid deep-learning approach for single-shot wavefront sensing and correction

Sina Moayed Baharlou[1,2†], Muhammad Waleed Khalid[1†], Guli Gulinihali[1], Jeongho Ha[1], Liyi Hsu[1], Samantha C. Lewis[3], Lei Tian[2], Yeshaiahu Fainman[1], Alexander V. Sergienko[2], and Abdoulaye Ndao[1,2*]

[1]Department of Electrical and Computer Engineering, University of California, San Diego, La Jolla, CA 92093, USA

[2]Department of Electrical and Computer Engineering & Photonics Center, Boston University, Boston, MA, USA

[3]Department of Molecular and Cell Biology, University of California, Berkeley, CA, USA

[†]These authors contributed equally

[*]Address correspondence to: a1ndao@ucsd.edu

1 Challenges of Single-Shot Systems

The goal of the proposed framework is to detect optical aberrations from a single-intensity image captured from various beam types. Existing approaches face limitations in achieving this task: First, they are generally restricted to using the Point Spread Function (PSF) and cannot directly detect aberrations from intensity images of other complex structured lights. Second, they require two or more intensity images captured at different focal planes to recover the phase due to the inherent focal plane ambiguity.

Focal Plane Ambiguity occurs when two or more phase aberrations produce nearly identical intensity distributions. The prevailing case is when a phase and its sign-negated-reversed produce the same intensity profile. In less frequent cases, different aberrations can produce similar effects on intensity images. For instance, vertical astigmatism and defocus can both result in the expansion of a Gaussian beam when the intensity of the aberration is low. Additionally, when the system is designed to work with different beam types, and the type is not known in advance, this can introduce ambiguity across different beams. For example, a first-order OAM beam that has vertical astigmatism aberration can produce a similar intensity profile as a Hermite Gaussian beam.

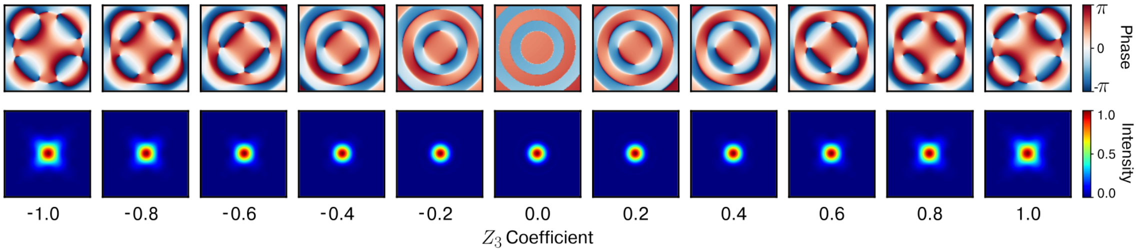

Figure S1: Gaussian beam aberrated with varying values of oblique astigmatism. The figure demonstrates the ambiguity issue, where aberrations produce similar effects regardless of their sign, and highlights plateau regions where changes in aberration coefficients within the range of -0.4 to 0.4 do not significantly affect the beam intensity.

When predicting aberration from a single image, ambiguity is not the only challenge, even when the system is designed to trade off accuracy to estimate sign-less aberration coefficients. Another challenge is the presence of plateau regions in the phase-to-intensity mapping. Plateau regions are areas where changes in aberration coefficients do not significantly affect the intensity. In those regions, the intensity profile provides limited information about the phase, making it more difficult for the system to learn the inverse mapping and accurately predict the aberrations.

Figure S1 illustrates a scenario where both ambiguity and plateau issues can be observed. This figure displays a Gaussian beam that is aberrated with varying values of oblique astigmatism (Z_3), ranging from -1 to 1. Both positive and negative aberration coefficients yield identical intensity profiles, highlighting the ambiguity. Additionally, the intensity profiles remain mostly stationary within the range of -0.4 to 0.4, which demonstrates the plateau issue.

In what follows, we demonstrate how a trained bias can resolve the ambiguities (disambiguation) and overcome issues caused by plateau regions (amplification).

2 Optical Model and Focal Plane Ambiguity

In this section, we first present the model used to represent optical aberrations, then demonstrate the most prevalent source of Focal Plane Ambiguity (FPA), and finally show how a single engineered bias can eliminate this ambiguity in the presence of wavefront aberrations.

Given a 2-dimensional input field $U_0(x,y) = U(x,y,z=0)$ at the entrance pupil of a focusing lens, the resulting field at the back focal plane, $U_f(x,y) = U(x,y,z=f)$, can be determined by:

$$U_f(u,v) = \frac{e^{ikf} e^{\frac{ik\left(x^2+y^2\right)}{2f}}}{i\lambda f} \mathcal{F}\left\{U_0\left(x,y\right) A_p(x,y) e^{i\varphi_w(x,y)}\right\}(u,v) \tag{1}$$

where λ is the wavelength, k is wavenumber, $A_p(x,y)$ is the aperture function, and φ_w is the wavefront aberration. The leading prefactor, including global and quadratic phase terms, does not affect the normalized intensity and can be omitted for simplicity:

$$U_f(u,v) \propto \mathcal{F}\left\{U_0\left(x,y\right) A_a(x,y) e^{i\varphi_w(x,y)}\right\}(u,v) \tag{2}$$

The input field is defined as $U_0(x,y) = A(x,y)e^{i\varphi_p(x,y)}$, where, $A(x,y)$ denotes the amplitude, and $\varphi_p(x,y)$ the phase profile of the beam, both assumed to be real-valued functions. The intensity distribution of the beam at the focal plane is given by:

$$I(u,v) \propto \left|\mathcal{F}\left\{U_0\left(x,y\right)A_a(x,y)e^{i\varphi_w(x,y)}\right\}(u,v)\right|^2 \tag{3}$$

2.1 Focal Plane Ambiguity

Since our primary focus is on phase ambiguity and the effect of aberrations on the intensity profile, we consider the case of a plane wave, $U_0(x,y) = 1$, and denote the aberration phase as $\varphi(x,y)$ for simplicity, instead of $\varphi_w(x,y)$. We then define the modified pupil function as $\tilde{U}_0(x,y) = A_a(x,y)\,e^{i\varphi(x,y)}$. Therefore, Equation 2 can be rewritten as:

$$U_f(u,v) \propto \iint \tilde{U}_0(x,y)e^{-2\pi i(\xi x+\eta y)}\mathrm{d}x\mathrm{d}y \tag{4}$$

The phase term $\varphi(x,y)$ can be written as a decomposition of even and odd symmetry functions:

$$\varphi(x,y) = \varphi_o(x,y) + \varphi_e(x,y) \tag{5}$$

Using the decomposed phase, removing the Euler representation, and performing the multiplication yields the following:

$$\begin{gathered}\tilde{U}_0(x,y) = A_a(x,y)e^{i\varphi_e(x,y)}.e^{i\varphi_o(x,y)} \\ = A_a(x,y)(\cos\varphi_o(x,y) + i\sin\varphi_o(x,y))(\cos\varphi_e(x,y) + i\sin\varphi_e(x,y)) \\ A_a(x,y)(\cos\varphi_o\cos\varphi_e + i\cos\varphi_o\sin\varphi_e + i\sin\varphi_o\cos\varphi_e - \sin\varphi_o\sin\varphi_e)\end{gathered} \tag{6}$$

To simplify our equations, we will set $A_a(x,y) = 1$ and omit it from our calculations. Each term in Equation 6 is either even or odd:

$$\tilde{U}_0(x,y) = \overbrace{\overbrace{\cos\varphi_o}^{\text{even}}\overbrace{\cos\varphi_e}^{\text{even}}}^{\text{even}} + i\overbrace{\overbrace{\cos\varphi_o}^{\text{even}}\overbrace{\sin\varphi_e}^{\text{even}}}^{\text{even}} + i\overbrace{\overbrace{\sin\varphi_o}^{\text{odd}}\overbrace{\cos\varphi_e}^{\text{even}}}^{\text{odd}} - \overbrace{\overbrace{\sin\varphi_o}^{\text{odd}}\overbrace{\sin\varphi_e}^{\text{even}}}^{\text{odd}} \tag{7}$$

Using an abbreviated form, we have:

$$\tilde{U}_0(x,y) = c_{\varphi_o}c_{\varphi_e} + ic_{\varphi_o}s_{\varphi_e} + is_{\varphi_o}c_{\varphi_e} - s_{\varphi_o}s_{\varphi_e} \tag{8}$$

The odd and even parts in the complex exponential of Equation 4 can be determined as follows:

$$e^{-2\pi i(\xi x+\eta y)} = \overbrace{\cos\big(\overbrace{2\pi(\xi x+\eta y)}^{\text{odd}}\big)}^{\text{even}} - i\overbrace{\sin\big(\overbrace{2\pi(\xi x+\eta y)}^{\text{odd}}\big)}^{\text{odd}} \tag{9}$$

Writing this equation in an abbreviated form, we obtain the following:

$$e^{-2\pi i(\xi x+\eta y)} = c_f - is_f \tag{10}$$

Substituting Equation 8 and 10 into 4, we obtain:

$$U_f(u,v) \propto \iint (c_{\varphi_o}c_{\varphi_e} + ic_{\varphi_o}s_{\varphi_e} + is_{\varphi_o}c_{\varphi_e} - s_{\varphi_o}s_{\varphi_e})(c_f - is_f)\mathrm{d}x\mathrm{d}y \tag{11}$$

By performing the multiplications and separating the integrals, we can determine the odd and even parts:

$$\begin{gathered} U_f(u,v) \propto \\ \iint \overbrace{c_{\varphi_o}c_{\varphi_e}c_f}^{\text{even}} - i\iint \overbrace{c_{\varphi_o}c_{\varphi_e}s_f}^{\text{odd}} + i\iint \overbrace{c_{\varphi_o}s_{\varphi_e}c_f}^{\text{even}} + \iint \overbrace{c_{\varphi_o}s_{\varphi_e}s_f}^{\text{odd}} \\ + i\iint \overbrace{s_{\varphi_o}c_{\varphi_e}c_f}^{\text{odd}} + \iint \overbrace{s_{\varphi_o}c_{\varphi_e}s_f}^{\text{even}} - \iint \overbrace{s_{\varphi_o}s_{\varphi_e}c_f}^{\text{odd}} + i\iint \overbrace{s_{\varphi_o}s_{\varphi_e}s_f}^{\text{even}} \end{gathered} \tag{12}$$

The integral of odd functions over symmetrical limits is zero; therefore, we can reduce Equation 12 to only four even terms:

$$U_f(u,v) \propto \iint c_{\varphi_o}c_{\varphi_e}c_f + i\iint c_{\varphi_o}s_{\varphi_e}c_f + \iint s_{\varphi_o}c_{\varphi_e}s_f + i\iint s_{\varphi_o}s_{\varphi_e}s_f \tag{13}$$

We can write Equation 13 in terms of the real and imaginary parts:

$$U_f(u,v) \propto \overbrace{\iint c_{\varphi_e}(c_{\varphi_o}c_f + s_{\varphi_o}s_f)}^{X:Real} + i\overbrace{\iint s_{\varphi_e}(c_{\varphi_o}c_f + s_{\varphi_o}s_f)}^{Y:Imaginary} \tag{14}$$

The intensity at the focal plane is determined as the sum of the squares of the following terms:

$$I_f(u,v) = |U_f(u,v)|^2 \propto X^2 + Y^2 \tag{15}$$

Since the focal-plane intensity depends only on the magnitude of the Fourier transform, any phase function $\varphi(x,y)$ and its sign-negated-reversed counterpart can produce identical intensity patterns. This introduces an ambiguity in which $\varphi(x,y)$ and $-\varphi(-x,-y)$ (the "ambiguous pair") yield the same focal-plane intensity, which we refer to as the **Focal Plane Ambiguity (FPA)**. To illustrate this symmetry, we decompose $-\varphi(-x,-y)$ into its even and odd components:

$$-\varphi(-x,-y) = -(\varphi_o(-x,-y) + \varphi_e(-x,-y)) = \varphi_o(x,y) - \varphi_e(x,y) \tag{16}$$

This shows that $\varphi(x,y)$ and $-\varphi(-x,-y)$ differ only in the sign of the even part. The intensity profile of this ambiguous pair can be determined as follows:

$$\begin{gathered} \varphi(x,y) = \varphi_o(x,y) + \varphi_e(x,y) \to U_f(u,v) \propto X + iY \to |U_f(u,v)|^2 \propto X^2 + Y^2 \\ -\varphi(-x,-y) = \varphi_o(x,y) - \varphi_e(x,y) \to U_f(u,v) \propto X - iY \to |U_f(u,v)|^2 \propto X^2 + Y^2 \end{gathered} \tag{17}$$

This shows that the intensity at the focal plane *does not* depend on the sign of the even component

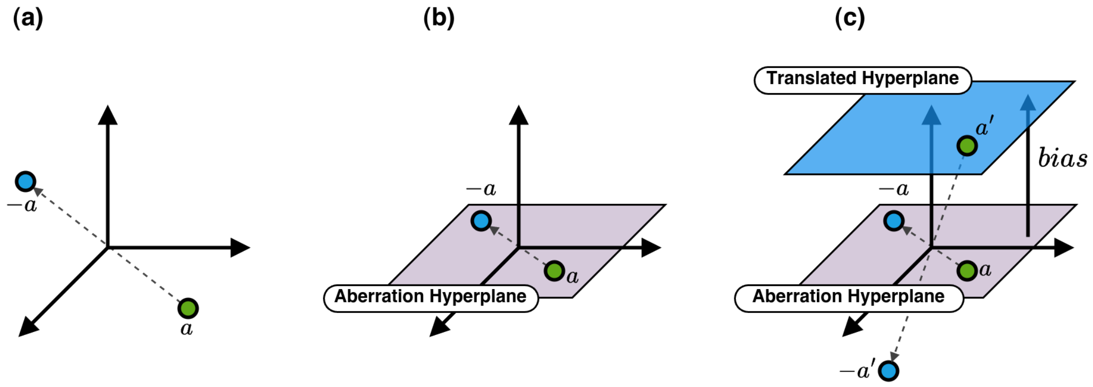

Figure S2: Illustrating the existence of ambiguity in the even phase space under different conditions. (a) Ambiguity exists throughout the entire space, when there are no constraints on representing the phase, (b) ambiguity exists within the aberration hyperplane, (c) ambiguity does not exist in the translated hyperplane, as the reflections of the points do not lie in the same hyperplane.

of the phase distribution.

2.2 Role of the Bias

We can represent the even component $\varphi_e(x, y)$ of the phase as a point in the even phase space, denoted by $\mathbf{a} \in \mathcal{R}^n$, where n represents the dimension of the space. This representation is achieved by vectorizing the discretized function $\varphi_e(x, y)$. Furthermore, $-\varphi_e(x, y)$ corresponds to $-\mathbf{a}$. According to Equation 17, if the odd part is fixed, any arbitrary point $\mathbf{a}$ in this space and its reflection $-\mathbf{a}$ will produce identical intensity distributions. Therefore, this ambiguity is present throughout the entire space, as illustrated in Figure S2.a.

2.2.1 Aberration Hyperplane

In case of wavefront aberrations, the phase distribution is typically a smooth function that can be represented in a lower-dimensional space using Zernike polynomials. We can write Equation 5 in terms of angularly even and odd Zernike terms as follows:

$$\varphi(x, y) = \sum_{odd\ l,r} \alpha_{l,r} Z_r^l + \sum_{even\ l,r} \beta_{l,r} Z_r^l \tag{18}$$

Where $\boldsymbol{\alpha} \in \mathcal{R}^k$, $\boldsymbol{\beta} \in \mathcal{R}^m$ are the coefficients of angularly odd and even polynomials, respectively. When we visualize the even part as points in the even phase space, these points are confined to a subspace within the larger phase space. This confinement occurs because the even part is expressed using $\boldsymbol{\beta}$, which has a lower dimension than the original space $m \ll n$. To better understand this, we let $m = n - 1$ so the aberration subspace can be viewed as a hyperplane. In this situation, point $\mathbf{a}$ cannot take arbitrary values but only those that lie on the aberration hyperplane.

The origin of the space also lies within the hyperplane ($\boldsymbol{\beta} = 0 \rightarrow \sum_{even\ l,r} \beta_{l,r} Z_r^l = 0$). Therefore, for any point on this hyperplane, the corresponding reflection also exist on the same plane. This leads to the persistence of ambiguity within that hyperplane, as illustrated in Figure S2.b.

2.2.2 Translated Hyperplane

The aberration hyperplane can be translated away from the origin in a way such that for any point on the translated plane $\mathbf{a}'$, its reflection $-\mathbf{a}'$ does not lie on the same plane (shown in Figure.S2.c). This translation can be expressed as a bias term $\mathbf{a}' = \mathbf{a}+\mathbf{b}$ where $\mathbf{b}$ is the vectorized form of $\varphi_b(x, y)$. This term can be interpreted as an additional phase shift that can be applied to the system using a single phase plate:

$$\Phi(x, y) = \varphi(x, y) + \varphi_b(x, y) = \sum_{odd\ l,r} \alpha_{l,r} Z_r^l + (\sum_{even\ l,r} \beta_{l,r} Z_r^l + \varphi_b(x, y)) \tag{19}$$

The aberrations are fully represented using $\boldsymbol{\alpha}$ and $\boldsymbol{\beta}$ as before, and since the reflection of the points does not lie in the translated hyperplane, all aberrations will have unique intensity distributions. This leads to a new subspace where the ambiguity does not exist. It is also important to note that the ambiguous pair in the original space will produce distinct intensity profiles in the translated space as well.

To make sure the origin doesn't lie within the translated hyperplane, the following condition must met:

$$\forall \beta_{l,r}\ , (\sum_{even\ l,r} \beta_{l,r} Z_r^l + \varphi_b(x, y)) \neq 0 \tag{20}$$

In essence, the bias should not be attenuated or canceled by the even component of the aberration. As a result, it needs to reside in a higher-dimensional space than the finite set of even Zernike modes supported by the system. Under this condition, **any bias with a non-zero even component that lies outside the span of the system's producible even Zernike polynomials can resolve the focal-plane ambiguity.** While, in principle, any bias meeting this criterion can lift the FPA, the specific pattern of the bias is crucial for optimal practical performance.

Given a limited camera frame that is also sensitive to noise, the even symmetrical phase should be chosen in a way to provide constructive interference with the beams, ensuring that ambiguous pairs and weak aberrations remain distinguishable. A bias with too low a magnitude may produce changes that are indistinguishable or undetectable by the camera. Conversely, if the magnitude is too high or if the bias consists primarily of high-spatial-frequency even components, it can scatter the beam, increase sensitivity to shot noise, and cause important information to fall outside the intended crop where the intensity is low. For example, we have shown Z_{12}^{-10} as the bias in Figure S3. This specific mode has a high spatial frequency and primarily induces changes outside the area of interest. In this figure, the intensity response of the two ambiguous pairs of oblique astigmatism looks almost identical. Given this, our goals is to learn the optimal bias using machine learning to ensure that important information remains distinguishable and contained within a limited crop.

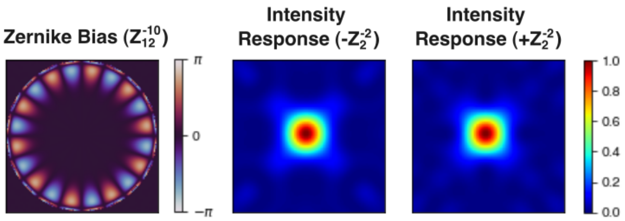

Figure S3: High-order Zernike polynomials as a bias, resulting in changes outside the cropped area.

2.2.3 Odd Components

The phase of the bias does not need to be purely even, it may include odd components as well. The necessary condition is the existence of a non-zero even symmetric component. Based on our experiments, the presence of odd components does not contribute to resolving the ambiguity, and in some cases, as we observed, having an odd component may even slightly decrease the accuracy by introducing additional distortions. Therefore, while a purely even phase is not strictly necessary, it's the even component that is essential.

3 Proposed Framework

The primary objective of our framework is to determine the phase aberration using a single-intensity image. In the previous section, we demonstrated how a single-phase mask can resolve the ambiguity caused by wavefront aberrations. This makes it possible to recover the aberration based on just one intensity image.

We propose a framework that utilizes Deep Neural Networks (DNNs) to accomplish this task. The framework consists of two main components: an optical part, which includes a trainable bias and is designed to eliminate the FPA and mitigate the impact of plateau regions, and a machine learning part, which is defined as a DNN responsible for detecting the aberration from the captured intensity images. We refer to latter component as the Aberration Prediction Network (APN). In our simulations and training, we utilize $\Phi(x, y)$ instead of $\varphi(x, y)$, which represents the phase when the bias is applied. In addition, in the experiments, we insert a phase plate before the focusing lens to induce the necessary phase shift. The framework is designed to detect aberrations not only from the aberrated intensity images of Gaussian beams but also from a variety of other structured beam profiles listed in Table S1.

The optical part is defined as a function that applies additional phase shift to the aberrated beam and captures the intensity image at the focal plane. This function maps aberrations to their unique intensity images:

$$\begin{gathered} O_{\varphi_b} : \varphi \to I \\ I = O_{\varphi_b}(\varphi; U_0) \propto \left| \mathcal{F} \left\{ U_0\left(x, y\right) A_a(x, y) e^{i[\varphi(x,y)+\varphi_b(x,y)]} \right\} (u, v) \right|^2 \end{gathered} \tag{21}$$

where U_0 indicates the beam type, A_a is the aperture function, and φ_b denotes the trainable bias term. The propagation scaling factor from Equation1 is omitted as it has no effect on the normalized intensity. APN then recovers the aberration using the single-intensity image and is defined as follows:

$$D_\theta : I \to \hat{\varphi} \tag{22}$$

Here $\hat{\varphi}$ is an approximation of φ, and θ represents the trainable parameters of the APN. The overall process is depicted in Figure 1 and can be expressed using the following expression:

$$\hat{\varphi} = D_\theta(O_{\varphi_b}(\varphi; U_0)) \tag{23}$$

Based on this, the framework can be viewed as an *autoencoder* [1], where the optical part serves as the encoder and the neural network functions as the decoder.

Autoencoders are special type of neural networks designed to transform the input data into a latent space, typically with the reduced dimension, using the encoder, and recover the original data from the latent space using the decoder. They are commonly used for feature extraction, dimensionality reduction, and denoising. Autoencoders can also be interpreted as models that learn to approximate the identity function. In this context, learning the identity function is beneficial as it ensures that we don't lose important information required to recover the aberration.

The goal is to find the optimal parameters θ and φ_b so that, for a beam with an unknown aberration φ, O_{φ_b} can eliminate the ambiguity, alleviate the impact of plateau regions, and produce a single intensity image, while D_θ, our APN, can estimate the aberration $\hat{\varphi}$ that is close to the true one given the intensity image. Once the parameters are determined in the simulation, O_{φ_b} can run optically while the neural network operates computationally.

We train our framework in an end-to-end fashion, where the optical part and the neural network are jointly trained. We express the aberrations using Zernike Polynomials, which allows us to represent the aberration only using the coefficients $\boldsymbol{\alpha}$ and $\boldsymbol{\beta}$. To simplify, we use a single term, $\mathbf{c} = [\boldsymbol{\alpha}; \boldsymbol{\beta}]$, which combines all the coefficients together. Therefore, the neural network only needs to estimate $\mathbf{c}$, a fixed number of Zernike coefficients:

$$\hat{\mathbf{c}} = D_\theta(O_{\varphi_b}(\varphi; U_0)) \tag{24}$$

Equation 18 can be used to determine the phase given the Zernike coefficients. To train the framework, we created large-scale datasets of optical and atmospheric aberrations and minimized the following Mean-Square-Error (MSE) function:

$$\min_{\theta, \varphi_b} \mathcal{L}(\theta, \varphi_b), \qquad \text{where } \mathcal{L}(\theta, \varphi_b) = \frac{1}{N}\frac{1}{C}\sum_{i=1}^{N}\sum_{j=1}^{C}(c_j^i - \hat{c_j^i})^2 \tag{25}$$

where $\mathcal{L}$ is the cost function, N is the number of training samples, C is the total number of Zernike

coefficients.

In our simulation, we developed and implemented a differentiable version of the Bluestein method [2] as our light propagation model, instead of using the Fourier transform. This approach allows us to accurately determine the intensity distribution at the focal plane while providing flexibility in defining regions of interest and adjusting sampling rates. The function E_θ is modeled using a modified Residual Neural Network [3]. The optimal architecture and training hyper-parameters are determined using a Hyper-Parameter Optimization (HPO) process utilizing the Optuna framework [4].

We implemented various symmetrical constraints, including arbitrary biases that consist of both odd and even components, as well as pure even symmetrical biases. In the case of the even configuration, we examined scenarios where only a quarter of the parameters are learnable, as well as a scenario where only one-eighth of the parameters are learnable. We refer to these latter configurations as radial biases, characterized by a repeating pattern when rotated by a specific degree, similar to that of a starfish. Additionally, we constrained the bias values between $-\pi/2$ and $\pi/2$ using a *TanH* function to prevent the beam from scattering.

Since the framework is designed to learn the identity function between φ and $\hat{\varphi}$, and given that D_θ is defined as a discriminative neural network which is only capable of learning one-to-one mappings, it is essential that the mapping between the aberration and the intensity images is also one-to-one. Initially, during the training process, the bias phase distribution is set to zero, leading to phase ambiguity, and one-to-many mappings in the inverse problem. As a result, the network cannot differentiate between φ and $\bar{\varphi}$. Therefore, a significant portion of the error from Equation 25, arises from this ambiguity. However, since the bias is trainable and given the imposed constraints, the phase distribution is updated step by step using backpropagation to minimize this error. Another part of the error is caused by APN's mistakes in predicting the aberrations in the plateau regions. The bias is also trained to minimize this error by shifting the aberration space to a subspace where small changes in aberration result in more significant changes in the intensity profile. Over time, the phase distribution converges to a value where all aberrations have distinct and distinguishable intensity profiles, which also enables the neural network to learn the inverse mapping efficiently. In this approach, since the neural network relies on the images to detect the aberration, it ensures that the bias is adjusted in a way that the differences between the ambiguous pairs are distinguishable and keeps any informative changes within the image crop.

Next, we will discuss the various datasets we generated to train and evaluate our framework, and provide more details regarding the training parameters and results.

4 Datasets

We generated several datasets to train and evaluate the efficiency of our framework. These datasets contains the Zernike coefficients, together with indices of the assigned beam profile (listed in Table. S1). Since the intensity images are not constant, they are generated within the framework given the aberration and the current bias. Throughout this work, aberrations are represented using either the first nine polynomials (third radial order), employed in both simulations and experiments,

or the first fourteen polynomials (fourth radial order), used in simulations only. These coefficients capture the most common aberrations encountered in optical systems. To demonstrate the model's adaptability to higher-order aberrations, we additionally utilized datasets generated using the first 27 polynomials (sixth radial order). For robustness experiments, datasets with aberrations expressed using up to 55 polynomials (ninth radial order) were also generated.

To determine the optimal bias and train the APN, we utilized datasets with uniformly sampled Zernike coefficients, and to validate the framework's efficiency, we tested it on more realistic datasets including the Kolmogorov [5, 6] and one-hot datasets. Below, each of these datasets is discussed in detail.

Index	Beam Profile	Order
1	Gaussian Beam	-
2	OAM	L=1
3	OAM	L=2
4	OAM	L=3
5	HG	N=1, M=0
6	HG	N=0, M=1
7	HG	N=1, M=1
8	LG	N=1, L=0
9	LG	N=2, L=0
10	LG	N=3, L=0

Table S1: Different beam profiles used in the proposed framework.

4.1 Datasets with Uniform Coefficients

The aberrations in this dataset are generated using the first nine polynomials (Z_9), and the coefficients are uniformly sampled within the range of -1 to 1. There is no correlation between the polynomials and each of them has an equal chance of appearance. The dataset consists of 100,000 samples, each randomly assigned with a different beam profile. In this dataset, 90% of the data is used for training, 5% for validation, and 5% for testing. The strength of each aberration can be determined by the Root-Mean-Square (RMS) function:

$$RMS(\varphi) = \sqrt{\frac{1}{n}\sum_{x,y}\varphi(x,y)^2} \tag{26}$$

Here n denotes the total number of pixels.

4.1.1 Dataset with Adjusted Strength

We created a dataset similar to the one described in Section 4.1, where the RMS strength is sampled from a normal distribution with a mean of $\pi/2$ and a standard deviation of $\pi/8$. This new dataset captures a broad range of aberration strengths, and the coefficients can have values greater than 1. In addition, we created another dataset with aberrations expressed using the first fourteen polynomials

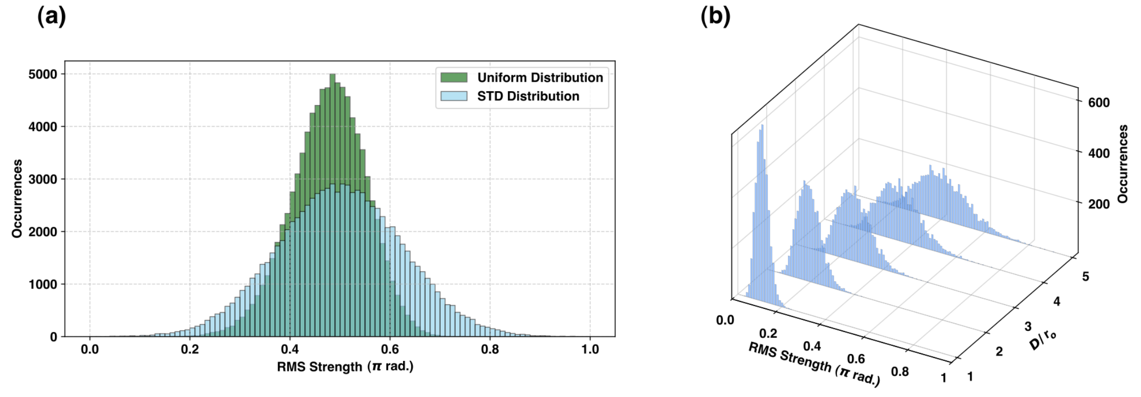

Figure S4: The RMS strength distribution of the generated datasets. (a) the RMS strength distribution of the dataset with uniform coefficients, and the dataset with adjusted standard strength, (b) the RMS strength distribution of the Kolmogorov dataset given different D/r_o values.

(Z_{14}). This dataset contains $500,000$ samples, with the same portion of training, validation, and test sets.

The distribution of aberration strength of these two datasets is depicted in Figure S4.a.

4.2 Kolmogorov Dataset

When dealing with more realistic aberrations caused by atmospheric turbulence, we typically rely on the Kolmogorov turbulence model [5, 6]. In this model, the Zernike coefficients are not statistically independent. Instead, they follow a Gaussian distribution with a mean of zero and a specific covariance matrix. This covariance matrix can be derived as follows:

$$\Sigma_{zz'} = \frac{K_{zz'}\delta_z\Gamma\left(\frac{n+n'-5/3}{2}\right)(D/r_o)^{5/3}}{\Gamma\left(\frac{n-n'+17/3}{2}\right)\Gamma\left(\frac{n'-n+17/3}{2}\right)\Gamma\left(\frac{n+n'+23/3}{2}\right)} \tag{27}$$

where n and m are the radial and azimuthal orders of z_j, respectively, while n' and m' represent the radial and azimuthal orders for $z_{j'}$, both following Noll's convention. $(D/r_o)^{5/3}$ is scaling factor which depends on the aperture size D, and r_o which is Fried's parameter. δ_z and $K_{zz'}$ are expressed as follows:

$$\delta_z = (m = m') \wedge \left(\overline{\text{parity}(j, j')} \vee (m = 0)\right). \tag{28}$$

$$K_{zz'} = 2.2698(-1)^{(n+n'-2m)/2}\sqrt{(n+1)(n'+1)}. \tag{29}$$

The covariance matrix of the first 14 polynomials is presented in Table S2. This table shows that the polynomials that have the same angular frequency number are correlated. Additionally, it is evident that the variance (the diagonal values), which indicates a relative strength of the aberration modes, decreases significantly as we progress to higher-order polynomials.

Z_i	1	2	3	4	5	6	7	8	9	10	11	12	13	14
1	0.454	0	0	0	0	0	-0.014	0	0	0	0	0	0	0
2	0	0.454	0	0	0	0	0	-0.014	0	0	0	0	0	0
3	0	0	0.023	0	0	0	0	0	0	0	-0.004	0	0	0
4	0	0	0	0.023	0	0	0	0	0	0	0	-0.004	0	0
5	0	0	0	0	0.023	0	0	0	0	0	0	0	-0.004	0
6	0	0	0	0	0	0.006	0	0	0	0	0	0	0	0
7	-0.014	0	0	0	0	0	0.006	0	0	0	0	0	0	0
8	0	-0.014	0	0	0	0	0	0.006	0	0	0	0	0	0
9	0	0	0	0	0	0	0	0	0.006	0	0	0	0	0
10	0	0	0	0	0	0	0	0	0	0.002	0	0	0	0
11	0	0	-0.004	0	0	0	0	0	0	0	0.002	0	0	0
12	0	0	0	-0.004	0	0	0	0	0	0	0	0.002	0	0
13	0	0	0	0	-0.004	0	0	0	0	0	0	0	0.002	0
14	0	0	0	0	0	0	0	0	0	0	0	0	0	0.002

Table S2: Covariance matrix of the first 14 Zernike polynomials expressed in ANSI standard scheme. Z_i denotes the order of the polynomial, and $D/r_o = 1$.

Having the covariance matrix, we can sample points from this distribution using the SVD decomposition. We generated two test sets using the Kolmogorov Model: one that expresses aberrations using the first nine polynomials and another using fourteen polynomials. In both sets, we varied the scale parameter D/r_o value from 0 to 5, which characterizes the distortion strength. We generated $5,000$ samples for each of these sets. The RMS strength of these datasets is shown in Figure S4.b.

4.3 One-hot Dataset

In this dataset, aberrations are created using a single Zernike polynomial. The generation process is as follows: a polynomial is chosen randomly from Z_1 to Z_9 or Z_{14} then its coefficient is uniformly sampled within the range of -1 to 1. We generated two test sets, each containing $5,000$ samples, for Z_9 and Z_{14}.

5 Training Process

The optical part is simulated using a differentiable version of the Bluestein method [2] that we implemented. This method takes a complex field as the input and calculates the propagated field at the given distance. By using a focusing lens with the input field, we can obtain an accurate intensity distribution at the focal plane. The Bluestein method operates with a fixed wavelength of 400 nm to generate the intensity images. Since we capture the intensity at the focal plane, changes in wavelength only change the geometrical scale of the intensity image (λ/2NA). As a result, capturing the intensity shape with just one wavelength is sufficient. To ensure that the model remains scale invariant in the entire bandwidth, the APN used in experiments is trained with different scale sizes that mimic variations in wavelength within the bandwidth (explained in Section 7.2).

In our simulations, the source field represents the aberrated beam augmented with the phase profile of the bias. The complex field is discretized into 128×128 pixels. The width of the input field is 8640 μm, while the output field has a smaller width size of 250 μm. We calculate the normalized intensity from the output field, and feed it into our APN. The output of the network

consists of the predicted Zernike coefficients $\hat{\mathbf{c}}$.

The entire framework is implemented in PyTorch [5] and runs on the Linux operating system with an RTX 3080 GPU.

5.1 Hyper-Parameter Optimization

The optimal architecture of the APN, along with the training hyper-parameters, is determined through the Hyper-Parameter Optimization (HPO) process using the Optuna framework [4]. Given a predefined range of parameters, Optuna explores this space to find the best settings that minimize the objective function.

We defined a smaller dataset with $40,000$ samples and ran the HPO process for 100 trials. The distribution of the parameters is as follows: The batch size can take values of 32 or 64, and the learning rate is sampled from a logarithmic uniform distribution within the range of 10^{-5} to 10^{-2}. When determining the network architecture, HPO can select from simple convolutional neural networks to more complex, deeper residual ones.

The network can utilize fully connected layers, with different configurations listed in Table S4. It may also incorporate skip connections. The configuration for the convolutional layers is sampled from the items listed in Tables S5 and S6. Table S5 displays the number of residual blocks, while Table S6 shows the number of channels for each block. During the HPO, the bias was allowed to learn both even and odd components. Interestingly, in nearly all training runs, the learned bias spontaneously evolved into a predominantly even symmetrical pattern. This indicates that the optimization process naturally suppresses odd components. Figure S30 provide several examples where the bias converges into even symmetrical shapes. The final performance is evaluated using the MSE on the validation set. Table S3 lists the top five configurations with the lowest error values. We selected trial number 43 as our optimal setting because it achieved strong results while using fewer network weights. The final architecture is a modified version of ResNet34 [3].

Trial Number	Batch Size	Learning Rate	Enable FC Layer	FC Layer Config	Skip Conn.	ResNet Blocks IDX	ResNet Channel IDX	MSE Val
59	32	0.001857	True	2	True	2	1	0.000755
43	64	0.004056	False	-	True	1	1	0.000894
75	64	0.002922	True	1	True	2	1	0.000979
5	32	0.001668	False	-	True	2	2	0.001024
96	64	0.002981	True	1	True	1	1	0.001055

Table S3: The network configuration and hyper-parameters for the top five trials determined by the HPO process.

5.2 Training Results

We trained our framework using the obtained hyper-parameters for 100 epochs on the dataset with uniform coefficients. This dataset ensures that the aberration space is evenly sampled and no

Index	Dense Layers
0	128
1	256, 128
2	512, 256, 128

Table S4: Dense Layer CFG

Index	ResNet Blocks
0	1, 1, 1, 1
1	1, 2, 2, 1
2	2, 2, 2, 2
3	3, 4, 6, 3

Table S5: ResNet Block CFG

Index	ResNet Channels
0	32, 64, 128, 256
1	64, 128, 256, 512
2	64, 128, 128, 128

Table S6: ResNet Channel CFG

polynomials are prioritized over the others. We deployed a learning rate scheduler that reduces the learning rate during the final epochs to improve convergence stability. The bias phase profile is trained for the first 20 epochs and then held fixed to prevent saturation. The network was trained using one arbitrary bias and two pure even symmetrical biases (Section 3). Ultimately, the radially symmetric bias (one-eighth) demonstrated the most stable performance and the highest accuracy. The learned phase distribution of this bias is illustrated in Figure S5.a. The training and validation curve is shown in Figure S5.b, which demonstrates the successful convergence of the network with no signs of underfitting or overfitting. This indicates that the framework successfully learned to eliminate FPA and map the intensity images to the corresponding aberration parameters.

To evaluate the network efficiency on the test set, we define the Root-Mean-Square Error (RMSE) function over the full rectangular (Cartesian) computational grid as follows:

$$RMSE(\varphi, \hat{\varphi}) = \sqrt{\frac{1}{n}\sum_{x,y}(\varphi(x,y) - \hat{\varphi}(x,y))^2} \tag{30}$$

This function measures the error in the phase space, with the same definition applied consistently across all models and experiments. The average RMSE obtained is $0.0194\,\pi$ radians. The distribution of errors is shown in Figure S5.c, and the average error in predicting each Zernike coefficient is reported in Figure S5.d. Figures S26 to S28 present a detailed analysis of the performance of our framework. Figure S26 displays the training and validation curves for each Zernike polynomial individually. Figure S27 illustrates the error distribution for each beam profile, showing a comparable distribution of errors across the different profiles. Meanwhile, Figure S28 highlights the average prediction error for each polynomial across all beam profiles, confirming the stable performance of our network.

We also provided several qualitative examples of the process of aberration detection and correction using our framework. Figure S6 presents instances where the aberrated and corrected beams are displayed alongside the true aberration and the detected aberration, both shown in Zernike and phase spaces. Figure S29 illustrates samples where the intensity profiles after the bias modulation are also shown.

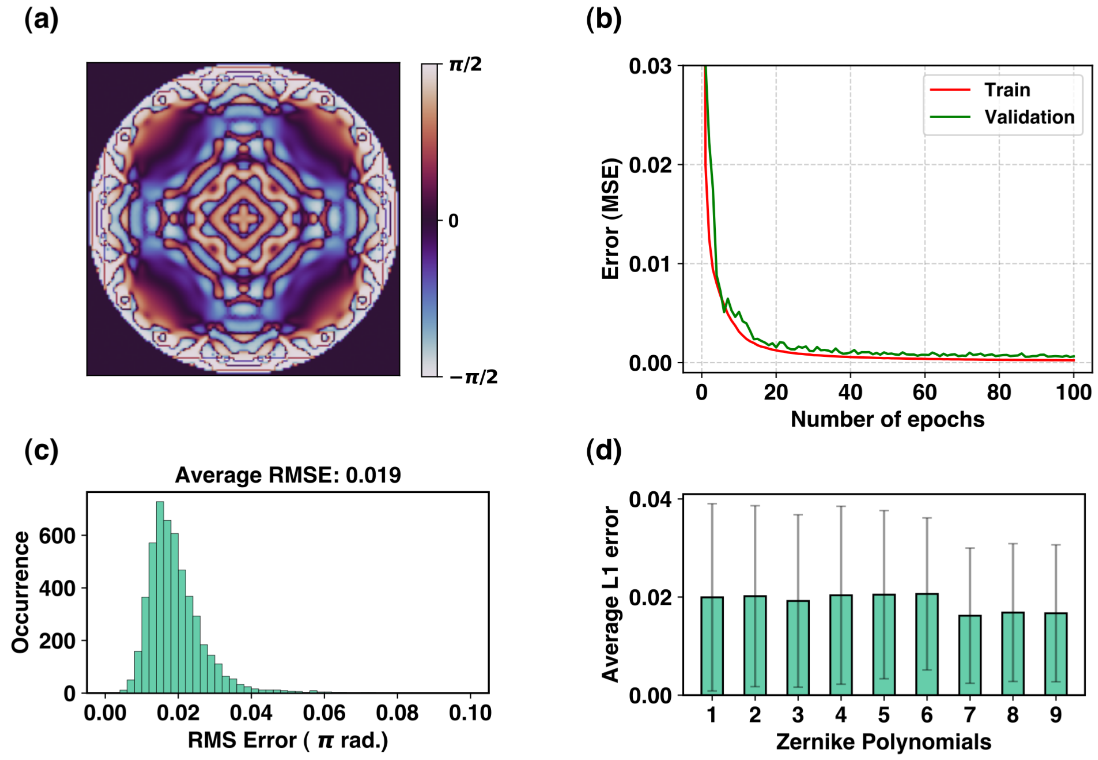

Figure S5: Results of the training process. (a) The optimal phase distribution of the bias obtained from the training process, (b) learning curve showing training and validation error over epochs, (c) error distribution on the test set, (d) average L1 error for each Zernike polynomial on the test set. Error bars represent the standard deviation (SD).

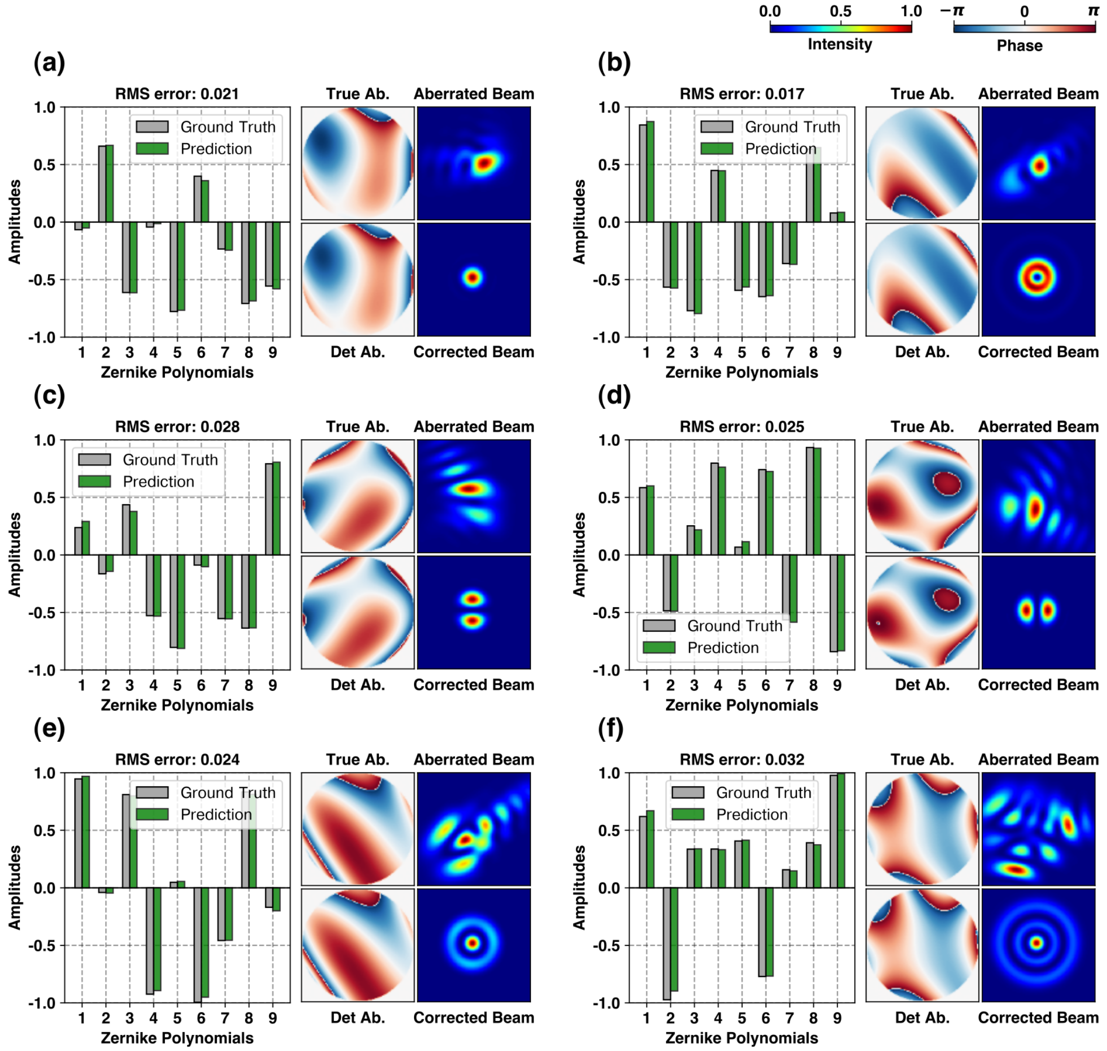

Figure S6: Qualitative examples of aberration detection using our framework on the dataset with uniform coefficients: (a) Gaussian, (b) OAM with $L = 1$, (c) $\mathrm{HG}_{1,0}$, (d) $\mathrm{HG}_{0,1}$, (e) $\mathrm{LG}_{1,0}$, (f) $\mathrm{LG}_{2,0}$. For each case, the corresponding ground truth and predicted aberration are shown in phase and Zernike space, alongside the aberrated beam and the corrected one.

6 Evaluation

To evaluate the framework under more realistic scenarios, we fine-tuned the APN on the dataset with adjusted strength, while keeping the bias phase constant. This enables the network to predict coefficients that are larger than one and improves its performance across a broader range of aberration strengths. A similar training parameters were used as in the previous section, and the network was trained on both Z_9 and Z_{14} datasets. The fine-tuned networks are evaluated on robustness test cases, Kolmogorov, and one-hot datasets.

6.1 Robustness Tests

6.1.1 Bias Comparison

In this experiment, we compare the effectiveness of our trained bias against several common phase profiles used as biases. We trained the APN using various biases on the dataset with aberrations generated by the first nine Zernike polynomials (Section 4.1.1). The biases were not trained; only the APN was. Initially, we established a baseline error by training the model without any biases. Following this, we incorporated low-order Zernike basis functions, specifically Z_3 and Z_4, as well as higher-order ones including Z_{12}, Z_{22}, Z_{23}, and Z_{37}. Additionally, we used two random phase profiles for comparison. The phase shift for each discretized pixel in the random bias is uniformly distributed between 0 and 2π. This creates a maximally random mask that generates a speckle intensity pattern at the focal plane, similar to the effect of an optical diffuser. We also designed a symmetric random mask by randomizing a quarter of the mask and then reflecting it to create the remaining portion. Figure S7.a shows the validation curve from the training process, clearly indicating that our trained bias consistently outperforms the others.

The lower-order Zernike-polynomial based biases lead to higher errors because they can be canceled out by their conjugate aberrations, e.g $+Z_3$ bias will be minimized or canceled by the $-Z_3$ aberration completely. In contrast, random phase profiles demonstrate better performance than lower-order polynomials. However, these random phase profiles are more vulnerable to photon shot noise due to beam scattering, causing the detector to collect fewer photons per unit area. Additionally, they are less resilient to phase noise.

Higher-order Zernike modes demonstrated improved performance compared to the lower modes and the random bias. This observation supports the theory discussed in Section 2.2.3. Ultimately, the trained bias outperforms the other biases because it is specifically optimized to eliminate ambiguity, create distributable patterns, and account for crop properties such as resolution and frequency range. Additionally, due to the complex, even symmetrical pattern of the trained bias, it is less likely to be canceled by higher-order aberrations compared to fixed biases.

Figure S7.b shows the mean and standard deviation of each bias evaluated on the test set. To emphasize the effectiveness of each bias, we have also plotted the individual RMSE for each beam profile (Figure S31). This figure demonstrates that our trained bias outperforms all other biases in every setting.

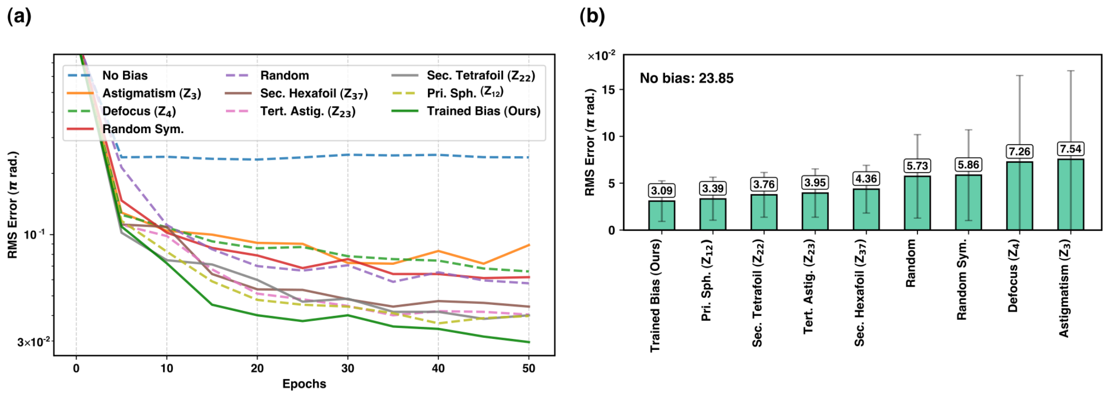

Figure S7: Comparison of the trained bias against other fixed biases. (a) the flow of the learning process and the corresponding validation errors, (b) the RMSE of the biases on the test set (Z_{12} value has been corrected). Error bars represent the standard deviation (SD).

6.1.2 Bias Interaction

We evaluated our framework in a scenario where there is only partial interaction between the beam and the bias size, concretely, when the beam's diameter is less than the bias. This situation can occur when the wavelength in the optical system varies. For this test, we re-trained the network with various beam-bias interaction ratios and evaluated the framework on a test set with a specified beam size. Figure S8.b shows a consistent behavior when the beam interaction area ranges from 0.5 to 1. This also confirms that the bias can eliminate the FPA even when the beam does not fully interact with the bias.

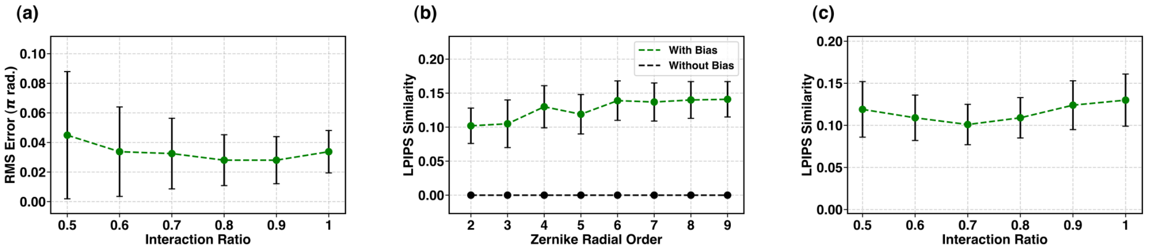

Figure S8: Demonstrating the robustness of our framework under different conditions. (a) Performance of the framework in terms of RMS error at different interaction ratios between the beam and the bias, (b) perceptual similarity between ambiguous pairs expressed using different Zernike orders, and (c) perceptual similarity between the ambiguous pairs with varying interaction ratios between the beam and the bias. Error bars represent the standard deviation (SD).

6.1.3 Ambiguous Pair Distinction

We performed a robustness test to assess whether the FPA is eliminated in higher-order aberration spaces by examining the intensity profiles of ambiguous phase pairs. We used the the Learned Perceptual Image Patch Similarity (LPIPS) metric [7] to quantitatively measure the perceptual

differences between a given phase and its corresponding ambiguous pair. In the absence of bias, such phase pairs produce identical intensity profiles, resulting in a one-to-many inverse mapping. This test therefore directly examines whether the learned bias renders previously ambiguous phases distinguishable, thereby improving injectivity without requiring explicit network training.

We created several data sets, each containing 5000 samples, where a random aberration is expressed using Zernike radial orders ranging from the second to the ninth order (Z_5 to Z_{55} polynomials). For each aberration $\varphi(x, y)$, we calculated $-\varphi(-x, -y)$ and determined their intensity profiles before and after bias interaction. We then used LPIPS to measure the similarity of each pair. This method offers a robust measurement of similarity based on the features extracted by trained DNNs. In this metric, zero signifies identical images, whereas one denotes substantial dissimilarity between them. This approach is computationally efficient and well-suited for high-dimensional aberration spaces (e.g., 55 Zernike modes), where direct training would require prohibitively large datasets due to the curse of dimensionality.

Figure S8.c shows the average LPIPS metric for the ambiguous pair with and without bias interaction. The results indicate that all ambiguous pairs produce distinct intensity profiles across a broad range of aberrations. This confirms that the bias effectively eliminates ambiguity in higher-order aberrations as well. Figure S8.d shows that the intensity profiles of the ambiguous pairs remain distinct, even when the beam partially interacts with the bias.

6.2 Plateau Regions

We conducted several experiments to demonstrate the existence of plateau regions, their impact on predicting optical aberrations, and how the trained bias mitigates the effects of these regions. As previously noted, plateau regions are areas where the intensity distribution exhibits low sensitivity to changes in aberration coefficients. Consequently, the system shows reduced accuracy when predicting aberrations within these regions. The plateau regions predominantly occur when the aberrations are not severe; that is, when the aberration coefficients are close to zero, as observed in Gaussian, Hermite-Gaussian, and Laguerre-Gaussian beams ($L = 0$). These beams show lower sensitivity to even symmetrical aberrations in these regions, as such aberrations primarily affect areas with lower intensity. An instance of this slow rate of change is shown in Figure S1.

To demonstrate this numerically, we calculate the Jacobian of the propagation function with respect to the aberration coefficients. The Jacobian and its norm quantify how the intensity distribution changes as the aberration coefficients vary. Figure S9 shows calculated components of the Jacobian (partial derivatives with respect to a specific aberration) of the Gaussian beam, both before and after using the bias, with all aberration coefficients set to zero. This figure illustrates how the intensity distribution changes when a specified aberration is introduced into the system. When the bias is utilized, the partial derivatives show much higher magnitudes and influence a larger area. As a result, the calculated norms have values that are at least 50 times greater. This implies that the intensity becomes more sensitive to the introduced aberrations when the bias is applied.

Figure S10 shows the calculated Jacobian norm on 2D cross-sections of the aberration space, illustrating the overall rate of change at various locations within this space. The blue regions indicate

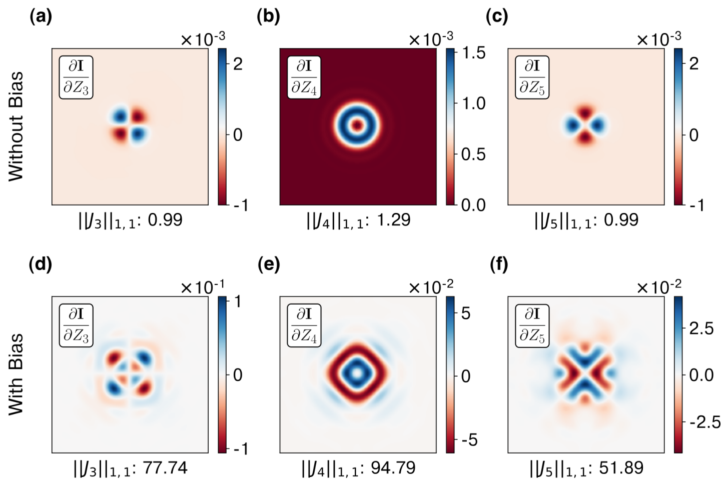

Figure S9: Partial derivatives (Jacobian components) of the intensity distribution of a Gaussian beam with respect to second-order Zernike aberrations, shown both before and after applying the bias. (a–c) the partial derivatives and their norms before using the bias, (d–f) partial derivatives and their norms after using the bias, demonstrating a significant increase in the norm.

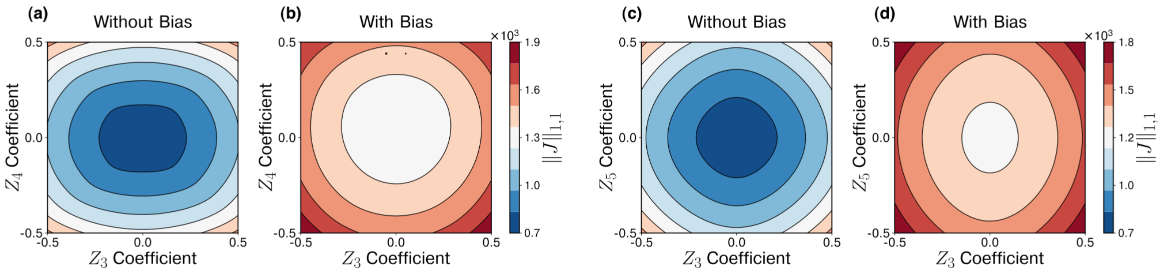

Figure S10: Jacobian norm calculated on 2D cross-sections of the aberration space (Gaussian beam), illustrating the rate of change before and after applying the bias. (a, b) Z_3–Z_4 space, (c, d) Z_3–Z_5 space. The bias increases the Jacobian norm, amplifying the effect of aberrations on intensity.

areas where the rate of change is minimal, referred to as plateau regions. After applying the bias, the rate of change increases, and the impact each aberration has on the intensity distribution is amplified.

In the next set of experiments, we demonstrate how the plateau regions affect the accuracy of aberration prediction and how the trained bias helps mitigate this issue. For comparison, we trained an additional network without using the bias, using the same configuration as the APN. To exclude the adverse effects of the ambiguities, this network was trained and evaluated only using the aberrations with positive even components in their phase distribution. We then compare the prediction accuracy of this network to the one that is using the bias.

The accuracy of the mentioned networks is evaluated across 2D cross-sections of the aberration space, as illustrated in Figure 2 and S33. Each point represents a beam with varying levels of aberration coefficients, with the color indicating the prediction error. Evaluations were conducted

both within the plateau region, where aberration coefficients are in the range of 0.0 to 0.1, and across a broader range from 0 to 1. It can be observed that the network with the bias performs significantly better than the one without, exhibiting much more consistent accuracy throughout the selected aberration space.

We performed an additional evaluation to further demonstrate the accuracy of our proposed framework both within plateau regions and in other areas of the aberration space. In this experiment, the beam is aberrated by a combination of two aberration coefficients, which are varied continuously between their positive and negative values in a circular pattern. For example, if the aberration terms are Z_3 and Z_5, this variation corresponds to a 180-degree rotation of the aberration pattern. We generated several aberration patterns using different aberration magnitudes: 0.1, 0.25, 0.5, and 1. The two networks from the previous section are tasked with estimating the corresponding aberration coefficients, and their predicted points are illustrated and compared to the ground truth.

Figure 2 and S34 present instances of this experiment, where the ground truth aberration is represented by a dashed circle, and the estimations from the two networks are shown as red and blue solid lines. The deviations of the red and blue curves from the dashed circle indicate the prediction errors of each network. The network without bias is not expected to predict the values in any negative region, as these were excluded from the training set to eliminate the impact of the ambiguity. However, this network also exhibits poor accuracy in the positive region, which shows the negative impact of the plateau regions. On the other hand, the network with the bias closely follows the dashed lines and successfully predicts both the magnitude and sign of the aberrations with high precision.

6.3 Atmospheric Turbulence

To verify the effectiveness of our framework, we evaluated it on Kolmogorov test sets designed to simulate aberrations caused by atmospheric turbulence. We generated two test sets using the first nine and fourteen polynomials. In each set, the aberration strength D/r_o varied from 0 to 5. 0 indicates no aberration (we selected a number close to 0 to maintain a consistent standard deviation), while 5 represents strong aberration.

We evaluated the framework and calculated the RMSE for each test set, achieving an average RMSE of 0.010 π radians across all D/r_o values for Z_9, and an RMSE of 0.014 π radians for Z_{14} (depicted in Figure 2.c-d of the main text). The error measured in the Kolmogorov dataset is generally lower than that in the uniform dataset, because the overall aberration strength of the Kolmogorov samples is slightly less than that of the uniform distribution.

We also present qualitative examples of the detection process for $(Z_{14}, D/r_o = 4)$ in Figure S11. Figures S35 and S36 illustrate the RMSE (in π radians) for the two sets, Z_9 and Z_{14}, respectively. The error is plotted for each beam profile separately, which shows consistent performance across these beams. Figure S37 shows six instances where an $HG_{2,2}$ beam is aberrated with increasing D/r_o strength, ranging from 0 to 5, and shows how the framework effectively detects these aberrations.

6.4 One-hot Dataset

We evaluated our framework using the one-hot dataset, which features aberrations represented by a single Zernike mode. The RMS error obtained on this test set is 0.013 π radians. The error distribution of this test is depicted in Figure S12.a, and the average prediction error for each polynomial is shown in Figure S12.b. We also included several quantitative examples of the detection process in Figure S13.

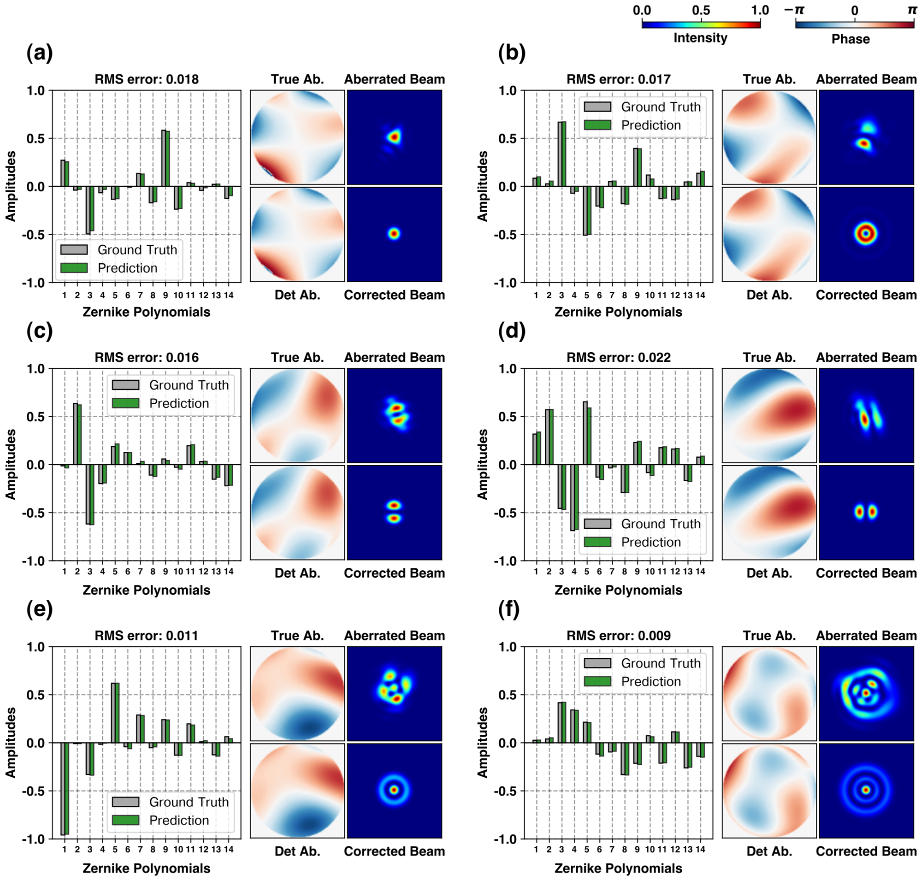

Figure S11: Qualitative examples of aberration detection using our framework on the Kolmogorov dataset ($D/r_0 = 4$): (a) Gaussian, (b) OAM with $L = 1$, (c) $HG_{1,0}$, (d) $HG_{0,1}$, (e) $LG_{1,0}$, (f) $LG_{2,0}$. For each case, the corresponding ground truth and predicted aberration are shown in phase and Zernike space, alongside the aberrated beam and the corrected one.

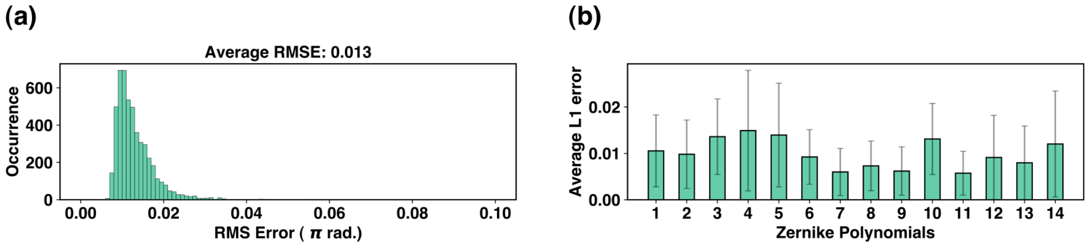

Figure S12: Evaluation results on the one-hot dataset. (a) Error distribution on the test set, (b) average L1 error for each Zernike polynomial on the test set. Error bars represent the standard deviation (SD).

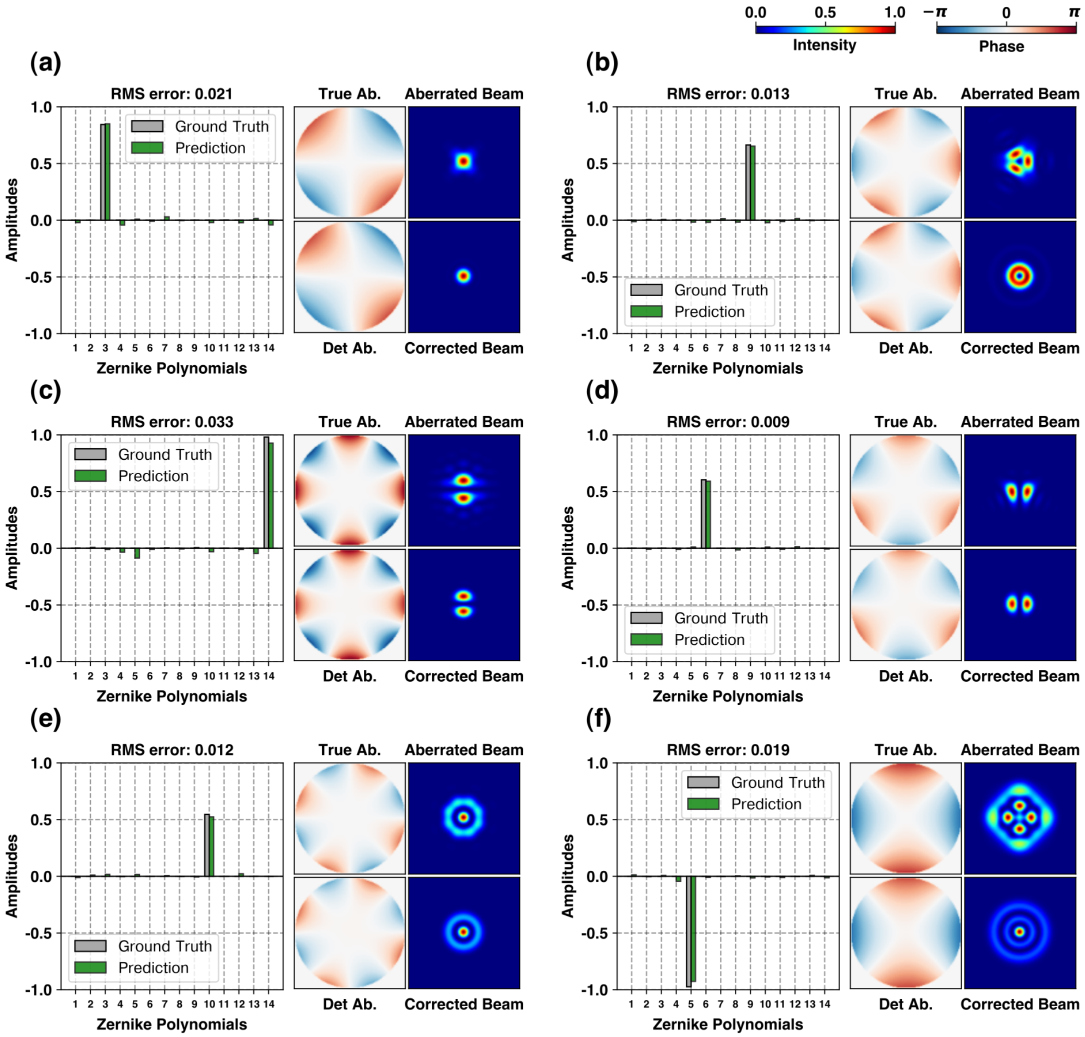

Figure S13: Qualitative examples of aberration detection using our framework on the one-hot dataset: (a) Gaussian, (b) OAM with $L = 1$, (c) $HG_{1,0}$, (d) $HG_{0,1}$, (e) $LG_{1,0}$, (f) $LG_{2,0}$. For each case, the corresponding ground truth and predicted aberration are shown in phase and Zernike space, alongside the aberrated beam and the corrected one.

6.5 Low-light Evaluations

To demonstrate the model's robustness in low-light conditions, which are characterized by a low signal-to-noise ratio (SNR), we conducted a series of evaluations where photon shot noise and camera read-out noise were modeled. During these evaluations, we utilized the APN, which was trained with a noise-robust module (explained in Section 7.2). This module acts as a regularizer, helping the network to prevent overfitting and learn the dominant features in the image instead of relying on individual pixel values.

We have utilized the following approach to model the SNR in the system [8]:

$$\mathrm{SNR} = \frac{\mathrm{S}}{\sqrt{\sigma_{\mathrm{S}}^2 + \sigma_{\mathrm{D}}^2 + \sigma_{\mathrm{R}}^2}} \tag{31}$$

Where S is the total detected number of photons, σ_{S}^2 is the photon shot noise, σ_{D}^2 is the dark shot noise, and σ_{R}^2 is the read noise. We assume a quantum efficiency of one, and model the shot noises, using the Poisson distribution and used the Gaussian distribution for read noise.

We evaluated our approach using two different configurations. In the first set of evaluations, we assumed that the pixel with the highest intensity for each aberration receives a fixed amount of average photons in the presence of dark shot noise ($\sigma_{\mathrm{D}}^2 = 2$) and read noise ($\sigma_{\mathrm{R}^2} = 4$). In this scenario, the SNR for each type of aberration remains constant, and each intensity image is subjected to a predefined fixed amount of noise. Specifically, the brightest pixel is assumed to receive between 100 and 900 photons, which, when considering dark shot and read noise, results in a linear SNR ranging from 9.71 to 29.90.

We evaluated the model using the Kolmogorov dataset (Z_9, $D/r_o = 5$), and the results are illustrated in Figure S14. The insets of the figure show a Gaussian beam that has been aberrated with a positive value of astigmatism. The results highlight the consistent performance of the network in the presence of shot noises and read noise.

In the second set of evaluations (photon-limited ones), we used identical exposure settings for all aberrations. To establish a reference, we assigned the photon count based on the brightest pixel in the diffraction-limited beam, which serves as the baseline for all other beams. For example, in the case of a 100-photon setting, the brightest pixel in the diffraction-limited beam receives, on average, 100 photons. For aberrated beams, the brightest pixel has a lower intensity than in the diffraction-limited case and therefore receives a proportionally smaller number of photons. This approach models a realistic scenario in which the total photon budget is limited, the camera exposure time is fixed, and different aberrations or phase plates produce focal-plane intensity patterns with varying brightness. For these evaluations, we focus solely on the effect of photon shot noise. The SNR for the brightest pixel varies with each aberration, it ranges from 10 to 30 for the diffraction-limited beam. The results of this experiment are illustrated in Figure S15. The findings indicate that the APN performs consistently well under low-light conditions, even when the captured intensity image is suboptimal. Several detection and correction instances of this process are illustrated in Figure S38.

Our approach focuses on a limited crop to minimize scattering of the beam (see Section 2.2.3). Scattering causes the photons to become more sensitive to shot noise, which results in the detector

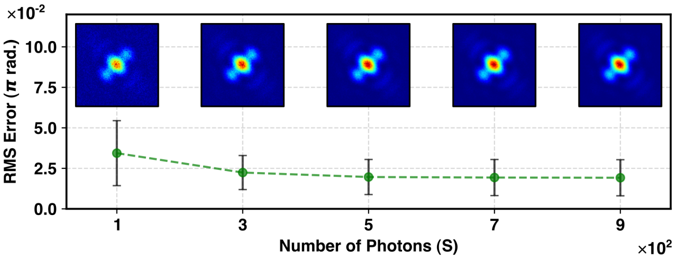

Figure S14: The performance of the model under low-light conditions. Evaluation has been done in the presence of photon shot noise, dark noise and read-out noise. The number of photons indicates the average photons the brightest pixel of each aberration receives. The insets in the figure show a Gaussian beam aberrated with a positive value of oblique astigmatism. Error bars represent the standard deviation (SD).

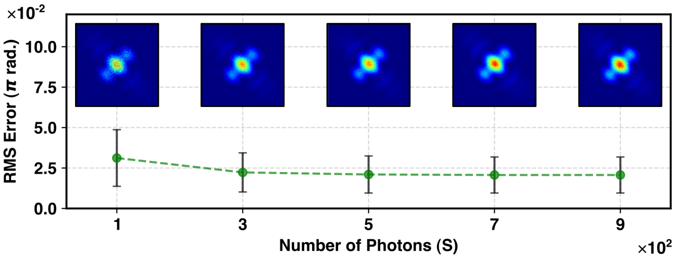

Figure S15: The performance of the model under low-light conditions. Evaluation has been done in the presence of photon shot noise. The number of photons indicates the average photons the brightest pixel a diffraction limited beam receives. The insets in the figure show a Gaussian beam aberrated with a positive value of oblique astigmatism. Error bars represent the standard deviation (SD).

collecting fewer photons per unit area. That is the reason why we prefer a bias that keeps the light as focused as possible. To gain a better understanding of this, we conducted additional simulations comparing the trained bias to a random phase bias discussed in Section 6.1.1. The random bias scatters the beam and creates a speckle pattern at the focal plane. We repeated the second set of evaluations with a fixed photon budget. Figure S39 demonstrates these results. The same intensity image receive fewer photons when using a random bias, making the pattern barely visible under low light conditions. Consequently, the error in predicting the aberration is large. In contrast, the intensity pattern using the trained bias retains its original shape, resulting in a lower error margin.

6.6 Out-of-Domain Evaluations

Up to this point, we have evaluated the proposed model using out-of-distribution samples, specifically the Kolmogorov and one-hot datasets. These samples are generated from the same underlying Zernike polynomials used during training, but feature a different coefficient distribution that the network has not encountered. To further demonstrate the generalizability of our model, we tested it

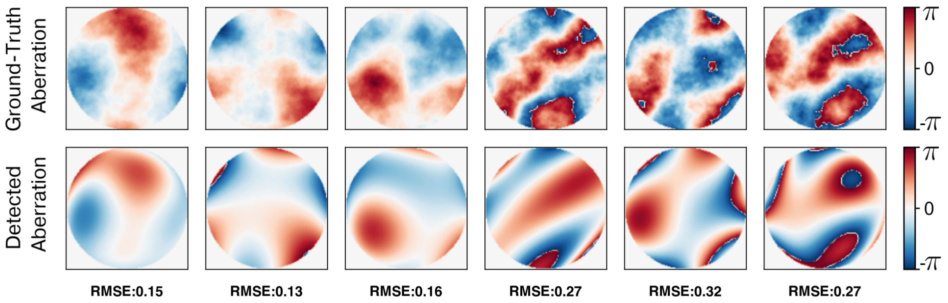

Figure S16: Evaluation instances of out-of-domain atmospheric turbulence. The first three instances on the left are generated with a Strehl ratio of 0.5, while the last three instances are generated with a Strehl ratio of 0.2. The error is represented in phase space using π radians.

on out-of-domain instances, particularly, aberration instances that are not directly generated based on the Zernike polynomials or those created using higher-order polynomials, which the network has not seen before.

We created three distinct categories of out-of-domain aberrations. The first category consists of atmospheric turbulences that are not directly generated by Zernike polynomials and includes a range of spatial frequencies that can alter the phase, and hence the intensity profile. The second category involves unstructured, smoothed random phase. To generate this phase profile, we create a low-resolution mask with random phases ranging from 0 to 2π, which is then scaled and smoothed. The third category includes aberrations produced using higher-order Zernike modes, particularly the first 7th radial order (35 Zernike coefficients).

For each aberration type, we evaluated performance under two different levels of complexity. For the turbulence model, we selected two Strehl ratios: 0.5 and 0.2, representing less and more severe aberrations, respectively. For the random smooth phase, we chose two different spatial resolutions of 8 and 15. Additionally, for the higher-order Zernike polynomials, we selected coefficients that are equally distributed across the modes, as well as a configuration with more dominant lower modes. For each complexity level we evaluated the model on 5000 random instances. Figure S16, S17 and S18 illustrates several instances from each of these experiments. The model effectively estimated the dominant low-order aberrations for both levels of complexity concerning turbulence aberrations, which further confirms its robustness in dealing with atmospheric turbulence. The obtained average RMSE for this experiment is 0.14 π radians for the SR of 0.5, and 0.31 π radians for SR of 0.2. In cases of random phase with less spatial fluctuations, the model identified the closest match within its representational capability. For random phases with more rapid fluctuations, the model attempted to find the best match; however, due to the increased complexity, it could only predict the center of mass of the phase distribution. The obtained average RMSE is 0.38 π radians for the spatial resolution of 8, and 0.36 π radians for the spatial resolution of 15. The same conclusion applies to Zernike polynomials expressed using higher-order terms. In each case, the predicted samples closely matched the lower modes of the generated aberrations without collapsing. The obtained average RMSE for the instances with equal mode distribution is 0.68 π radians, and for the ones

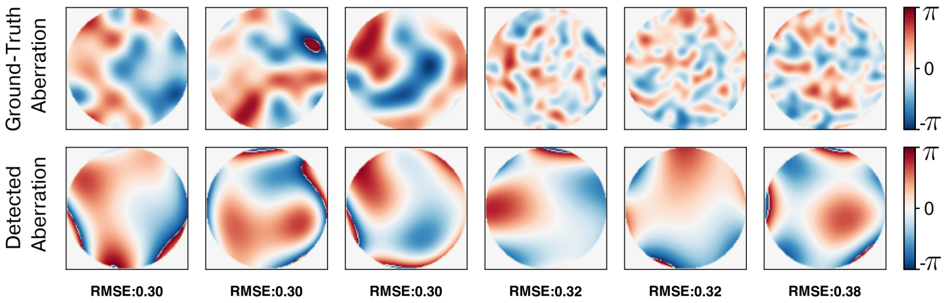

Figure S17: Evaluation instances of out-of-domain smooth random aberrations. The first three instances on the left are generated with a spatial resolution of 8, while the last three instances are generated with a spatial resolution of 15. The error is represented in phase space using π radians.

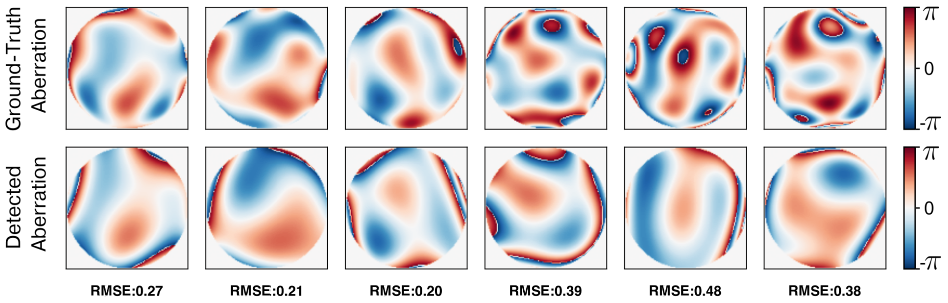

Figure S18: Evaluation instances of out-of-domain Zernike aberrations. The first three instances on the left are generated from the first 35 polynomials that have dominant lower modes, while the last three instances are generated from the first 35 polynomials with coefficients that are evenly distributed across the modes. The error is represented in phase space using π radians.

with dominant lower modes is 0.26 π radians.

The results confirm that the proposed network shows good generalizability for out-of-domain instances. Additionally, the findings indicate that the network has not over-fitted to the trained domain and has successfully learned the key features in the intensity images, which are essential for predicting the phase. We also trained the APN with a different architecture (UNet [9]) to directly estimate the aberration phase, in order to see if it would improve adaptability. However, our experiments revealed that it demonstrated the same level of adaptability as the model with fixed coefficients, though it came with increased computational cost.

6.7 Higher-order Training and Evaluation

To demonstrate the adaptability of the model with higher-order Zernike polynomials that express more complex aberrations, we extended our training and evaluation to a dataset with two additional radial orders (6th radial order, 27 Zernike coefficients). We created a dataset containing 1 million samples, and trained the model utilizing the same bias as before. We achieved an average RMSE of 0.074 π radians on the test set, which is practicable considering that the dimensionality increased

from 14 to 27. This experiment demonstrates that our proposed method can effectively eliminate ambiguity and detect more complex aberrations. The error distribution on the test set is illustrated in Figure S41, and several quantitative examples of the detection process are presented in Figure S40.

7 Experiments

For the practical experiments, the power of the Gaussian beam was around 50 μW. For the sensor, we used a BFS-U3-200S6C-C Blackfly USB 3.1 camera with an exposure range of 69 μs to 30 s and a gain range of 0–27 dB. All our data were collected at 0 dB gain and 80 ms exposure. For our experiments, we used a pulsed supercontinuum laser (NKT SuperK Fianium 15) together with an acousto-optic modulator to select the desired wavelengths. The tunable acousto-optic filter allowed us to isolate the specific wavelength of interest for each measurement. We then used a SuperK Connect module to couple the selected wavelength into a single-mode fiber, and an aspheric lens to collimate the output. This ensured that the beam emerging from the fiber was a high-purity Gaussian, providing a clean and well-defined input for all subsequent experiments.

To confirm the efficiency of our framework, we conducted two sets of experiments: (i) using only the SLM, which generated both the aberrations and the phase shift serving as the trained bias, and (ii) using the SLM to create the aberrations combined with a fabricated metasurface providing the trained bias as a separate optical element. A liquid crystal on silicon spatial light modulator (LCOS-SLM) was used to generate aberrations for different beams. We then captured their intensity images and estimated their aberration using our framework. Since the aberrations are manually created, we are able to demonstrate beam correction by subtracting the predicted aberration coefficients from the ground-truth ones and capturing the intensity again.

7.1 Optical setup calibration

We calibrated our setup to minimize the discrepancies between the simulation and the experiments. First, we corrected any inherent beam aberrations. Next, we ensured that the Zernike polynomials were defined in a canonical form for both the simulation and the experiment. We also confirmed that

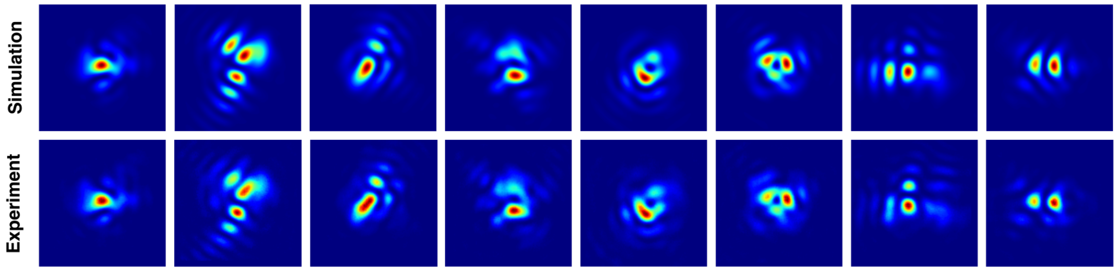

Figure S19: Comparison of simulated and experimentally generated aberrations. The top row shows simulation-generated aberrations, while the bottom row shows aberrations experimentally generated using a Spatial Light Modulator (SLM).

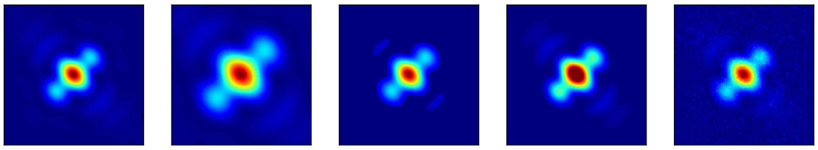

Figure S20: To ensure robustness, the framework was trained using images intentionally corrupted with common imperfections, including random cropping, random cut-off, random exposure variations, and Gaussian salt-and-pepper noise. As a result, the network accurately predicts the aberrated phase profiles from experimentally obtained images despite these imperfections.

the beam interacted fully with aberration and bias, and we adjusted the beam width to match our aperture size of 1 mm. Additionally, we separated the zeroth-order diffraction from the modulated beam by using a blazed grating. In addition, we carefully examined the intensity response of each beam to different Zernike basis functions to eliminate any further discrepancies. To maintain consistency, we evaluate our approach by looking at the extreme values of each Zernike basis function and ensuring that the total scaled aberrations match simulation predictions.
Figure S19 illustrates the simulated and experimentally generated aberrations after the calibration process, demonstrating their close alignment.

The wavelength-specific calibration we performed primarily involved adjusting the blazed-grating angle for each operating wavelength. A blazed grating works by directing most of the optical power into the "blazed" diffraction order, which corresponds to a specific angle. This angle depends on the wavelength, so changing the wavelength alters both the diffraction efficiency and the diffraction angle. Therefore, for each wavelength, we updated the blaze angle to ensure that the maximum power remained in the blazed order and that the diffracted beam followed the user-defined optical path. This is important for keeping internal aberrations minimal, particularly those corresponding to Z_1 and Z_2, which represent x-tilt and y-tilt respectively. We also ensured that the imaging lens used in the experiment had an AR coating suitable for the chosen wavelength and that it was achromatic. Apart from these adjustments, no additional wavelength-specific calibration was required in our experiment.

7.2 Noise Module

Several factors can still cause discrepancies between the intensity images generated in the simulation and those captured in experiments. Simulations are performed in ideal and noiseless settings, while experiments commonly encounter different types of noise. This noise can originate from the camera, SLM discretizations, fabrication imperfections in optical components, and more.

To overcome this issue, we introduce different types of noise during our simulations and retrained our framework. The updated framework can be modeled as follows:

$$\hat{\varphi} = D_\theta(\mathcal{G}(O_{\varphi_b}(\varphi + \varepsilon; U_0))) \tag{32}$$

Here ε represents the phase noise which follows a normal distribution $\varepsilon \sim \mathcal{N}(0, \left(\frac{\pi}{8}\right)^2)$. $\mathcal{G}(.)$ is a function that applies various types of noise to the intensity image, including cropping, low-intensity cut-off, overexposure, Gaussian noise, and salt-and-pepper noise, each applied randomly with a predefined probability. Figure S20 demonstrates the functionality of this function.

The phase noise ε ensures that the system is robust against phase modulation imperfections, whether the modulation is performed using SLM or a fabricated device. $\mathcal{G}$ extends this robustness to the noise during image capturing. This process can be viewed as a regularization technique, where the system learns to rely on the dominant features of the intensity image and discard the details that are altered due to the noise.

7.3 Results

7.3.1 Spatial light modulator

To quantitatively measure the performance of the framework in the first experiment, we evaluate it on the dataset with uniform coefficients and the Kolmogorov dataset (Z_9, $D/r_o = 4$). We randomly sampled 250 aberrations from each dataset and generated aberrated Gaussian, OAM, and HG beams. We obtained an average RMS error of 0.083 π radians for the uniform dataset and 0.069 π radians for the Kolmogorov dataset. The distribution of errors is shown in Figure S21a-c. This figure also depicts the error before incorporating the noise into our system, which demonstrates the improvement in detection accuracy after using the noise module.We collected several qualitative samples of the correction process in Figure 3a for the Kolmogorov dataset and Figure S22 for the uniform dataset.

We also tested the system using different wavelengths of 500, 600, and 700 nm. We ensured that the beam interaction area remained consistent as we varied the wavelength. This experiment was performed with the Gaussian beam and the dataset with uniform coefficients. Figure S21.d illustrates the achieved RMS error. Additionally, the qualitative samples of the detection process at different wavelengths are depicted in Figure S23.

The results show that the aberrations detected by our system closely match the ground truth aberrations across this 200 nm range. This confirms that our system operates effectively over a broad wavelength range, validating that our approach is not constrained by wavelength limitations.

7.3.2 Metaurface

To further demonstrate the effectiveness of the framework, we fabricated a TiO_2 metasurface to serve as the trained bias and position it in the setup (Figure S25). The fabrication process is explained in the manuscript and is depicted in Figure S43. The efficiency of the fabricated metasurface is shown in Figure S42. This experiment focused on ambiguity removal in a setup with fabrication imperfections introduced by the metasurface. Using Gaussian and OAM ($L = 1$) beams, the SLM applied aberrations based on the first nine individual Zernike modes with both positive and negative coefficients, while the metasurface provided phase shifts. The APN recovered aberrations and corrections were applied via the SLM (examples shown in Figure 4d). Figure S24 illustrates the intensity response of a Gaussian beam subjected to various aberrations corresponding to Zernike modes of opposite signs, along with comparisons to simulated intensity responses.

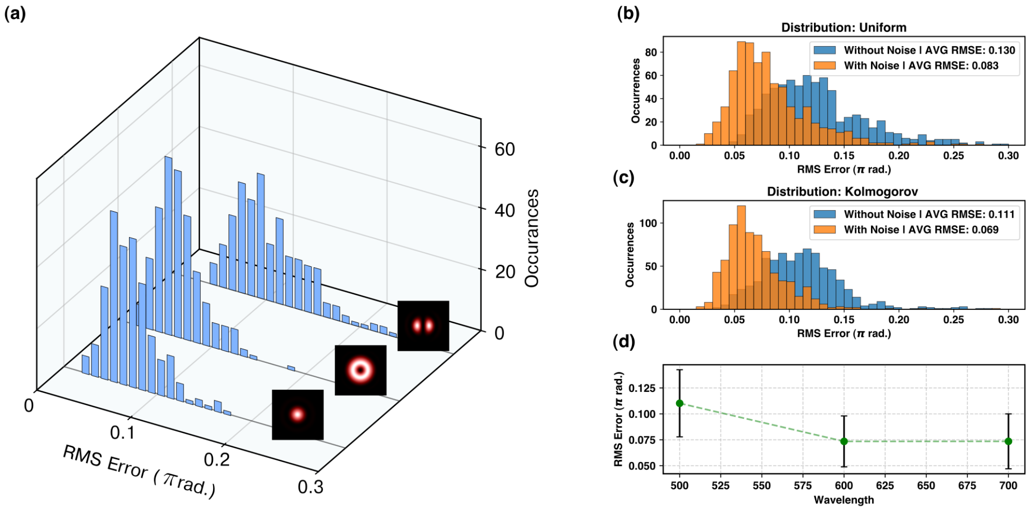

Figure S21: Quantitative results of the experiments. (a) Error distribution of the dataset with uniform coefficients calculated independently for each beams, (b) error distribution of the dataset with uniform coefficients, both before and after the incorporation of the noise module, (c) error distribution of the Kolmogorov dataset, also before and after adding the noise module, (d) performance of the framework across various wavelengths. Error bars represent the standard deviation (SD).

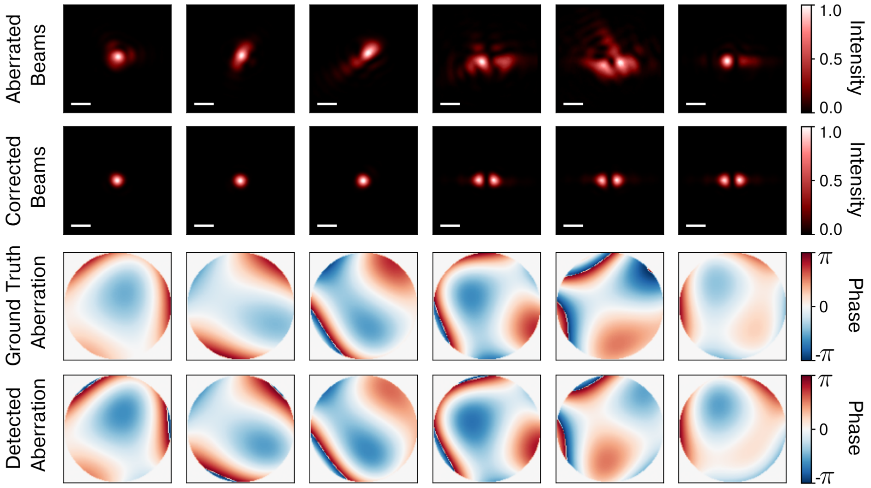

Figure S22: Qualitative examples of aberration detection and correction process experimentally performed at the wavelength of 700 nm. The first row illustrates different aberrations applied to Gaussian, and $HG_{1,1}$ beams, with three beams each. The second row shows the resulting beams after aberration correction using the neural network. The third row displays the ground truth aberrations used to create the aberrations. The last row presents the aberrations detected by the experimental system. Scale bars represent 200 um.

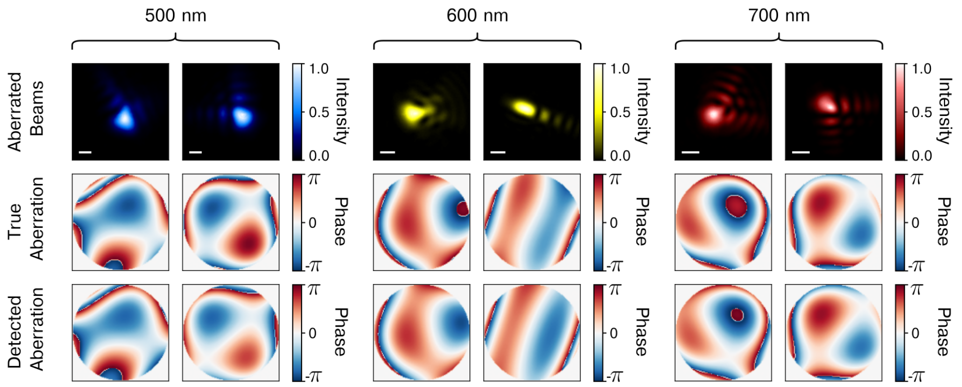

Figure S23: Verification of the broadband capability of our framework. Experiments were conducted at wavelengths of 500 nm, 600 nm, and 700 nm to test the network's performance. Scale bars represent 200 um.

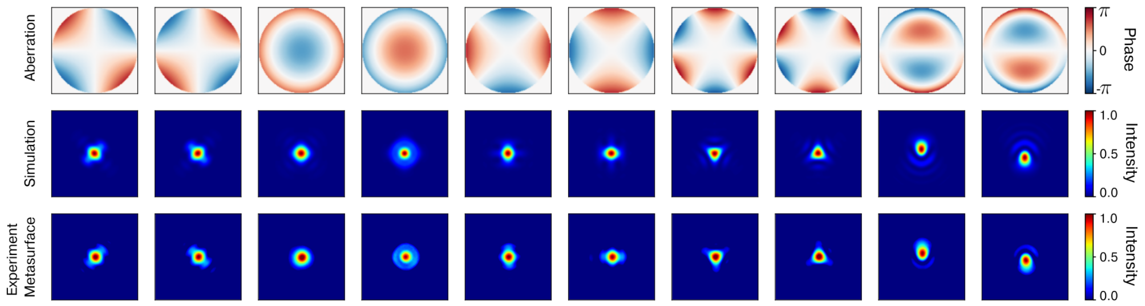

Figure S24: Intensity response of a Gaussian beam subjected to different aberrations (aberration maps shown in the first row), corresponding to Zernike modes of opposite signs. Results are shown for simulation (second row) and experiment using the fabricated metasurface (third row). The simulation accounts for the metasurface efficiency and the beam size used in the experimental setup.

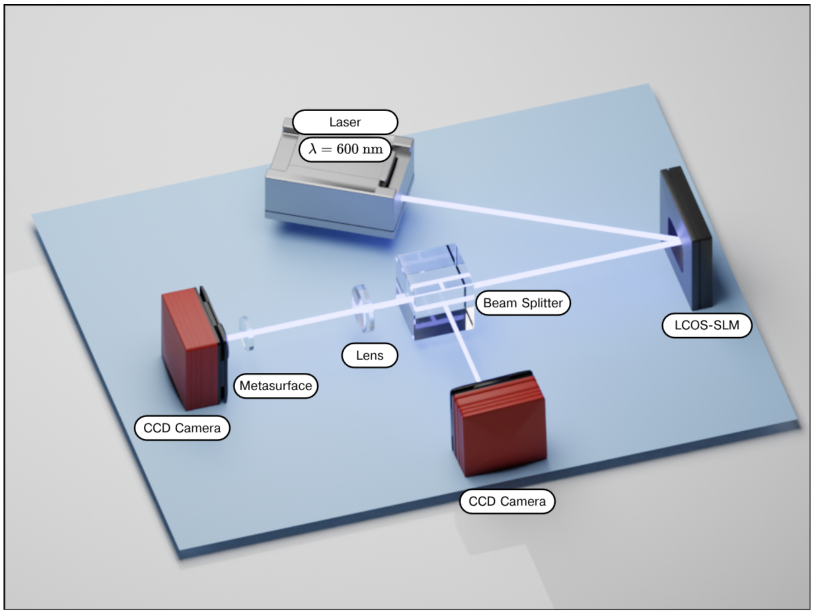

Figure S25: Experimental setup for the metasurface experiment. The beam is first aberrated using the SLM and then directed to a beamsplitter. One path passes through a focusing lens and the metasurface, which applies a phase shift to resolve phase ambiguity and enhance sensitivity to aberrations, before being captured by a CCD camera. The resulting intensity image is processed using the APN network to determine the aberrations, which are then uploaded to the SLM for beam correction. The corrected beam is directed along the other path of the beamsplitter and captured by a second camera.

7.3.3 Comparison

To demonstrate the effectiveness of the trained bias in eliminating the FPA and to highlight the contribution of our neural network-based estimator to the overall accuracy of the system, we compared our method to existing intensity-based techniques. Specifically, we analyzed the performance of our approach against the Gerchberg-Saxton (GS) algorithm and a Gradient Descent-Based (GDB) optimization method, assessing both with and without the trained bias.

The GDB method leverages the gradient information to iteratively update either the phase or the Zernike coefficients to minimize the discrepancy between the numerically generated intensity image and the experimentally captured one. The implemented GDB method uses the Adam optimizer [10] and incorporates the Bluestein propagation [2] method to get the intensity at the focal plane. The GS algorithm, utilizes the Fast Fourier Transform (FFT) to propagate the beam at the focal plane. To achieve the same frequency range and scale as the Bluestein method, we needed to zero-pad the FFT to a larger size, which increases the computational complexity of the method.

We conducted experiments using both the uniform and Kolmogorov datasets, which were collected experimentally. Table S7 and S8 displays the obtained RMSE for each of the datasets, confirming that our proposed method outperforms both the GDB and the GS algorithm.

By incorporating the trained bias, we have improved not only the neural network-based estimator but also the gradient-based one, demonstrating the effectiveness of the trained bias in enhancing the injectivity of the function. This result also indicates that once the bias is trained using our proposed approach, it can be utilized in other methods. Although this may lead to reduced performance, even if it may provide greater flexibility.

Both neural network-based and gradient-based models come with their own advantages and disadvantages. The neural network approach requires a large dataset for training and is typically less flexible than gradient-based methods. It may also display undesirable behavior if trained on a limited dataset or if the appropriate regularization techniques are not used. However, neural networks often deliver more accurate and faster predictions, and they are generally more robust to noise.

On the other hand, gradient-based models offer greater flexibility and interpretability. However, they tend to be slower due to their iterative nature and are prone to getting stuck in local minima. Additionally, the choice of the initial point can significantly impact their performance. The obtained results confirm that the GDB underperforms compared to APN because of the mentioned issues. GS algorithm did not show any improvements when incorporating the bias. It generally performed poorly in aberration detection because it lacks direct optimization and was designed to recover smooth, low-order, subtle aberrations. This makes it highly sensitive to noise and prone to stagnation.

**Model**	Gaussian	OAM (l=1)	HG_{10}	Total
GS w/o Bias	45.2 ± 12.0	40.3 ± 12.4	45.5 ± 12.2	43.7 ± 12.4
GS w Bias	46.0 ± 13.5	41.6 ± 12.7	46.2 ± 12.1	44.6 ± 13.0
GDB w/o Bias	33.3 ± 22.7	29.8 ± 23.5	36.5 ± 20.8	33.2 ± 22.5
GDB w Bias	16.7 ± 18.1	19.5 ± 22.9	25.1 ± 20.4	20.4 ± 20.9
APN w/o Bias	29.3 ± 12.7	12.5 ± 4.5	32.7 ± 12.5	24.8 ± 13.8
**APN w Bias (ours)**	$\mathbf{7.4 \pm 2.7}$	$\mathbf{6.7 \pm 2.3}$	$\mathbf{11.0 \pm 4.6}$	$\mathbf{8.3 \pm 3.8}$

Table S7: Comparison of the proposed method to other intensity-based methods experimentally evaluated on the dataset with uniform coefficients (Average RMSE $10^{-2} \times \pi$ rad.).

**Model**	Gaussian	OAM (l=1)	HG_{10}	Total
GS w/o Bias	29.5 ± 9.9	23.8 ± 8.3	32.3 ± 13.0	28.5 ± 11.1
GS w Bias	30.2 ± 11.4	24.3 ± 8.3	31.2 ± 12.3	28.5 ± 11.2
GDB w/o Bias	23.4 ± 19.8	11.8 ± 14.4	26.0 ± 18.2	20.4 ± 18.6
GDB w Bias	8.2 ± 14.9	8.1 ± 8.4	17.8 ± 13.9	11.4 ± 13.5
APN w/o Bias	22.4 ± 10.6	11.0 ± 4.2	27.7 ± 12.4	20.4 ± 12.0
**APN w Bias (ours)**	$\mathbf{5.9 \pm 1.7}$	$\mathbf{5.7 \pm 1.6}$	$\mathbf{9.0 \pm 3.3}$	$\mathbf{6.9 \pm 2.8}$

Table S8: Comparison of the proposed method to other intensity-based methods experimentally evaluated on the Kolmogorov dataset (Average RMSE $10^{-2} \times \pi$ rad.).

8 Additional Results

In this section, the additional results from Section 5 to 7 are illustrated.

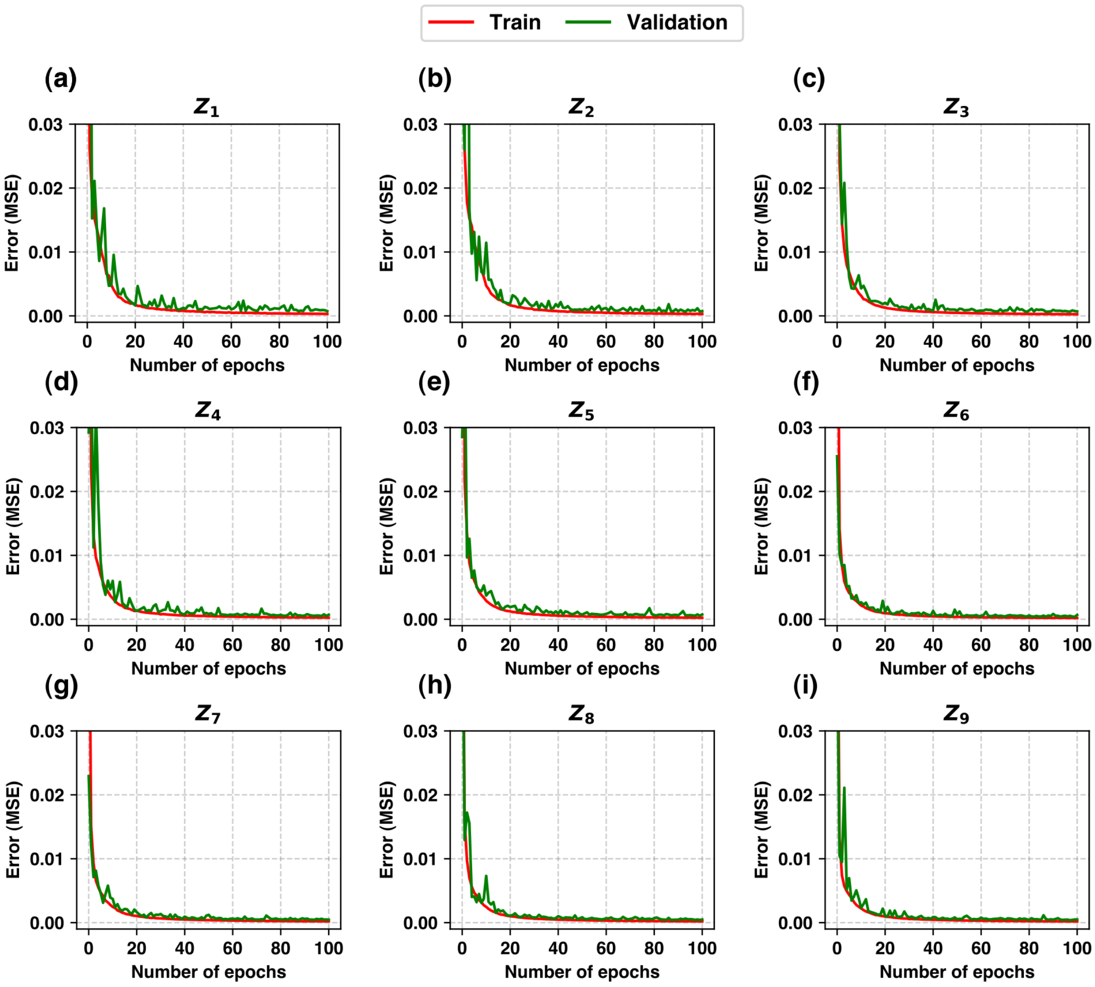

Figure S26: Learning curves segmented by individual Zernike polynomials, illustrating the training and validation errors for each term. The curves demonstrate stable convergence, indicating that neither underfitting nor overfitting is present.

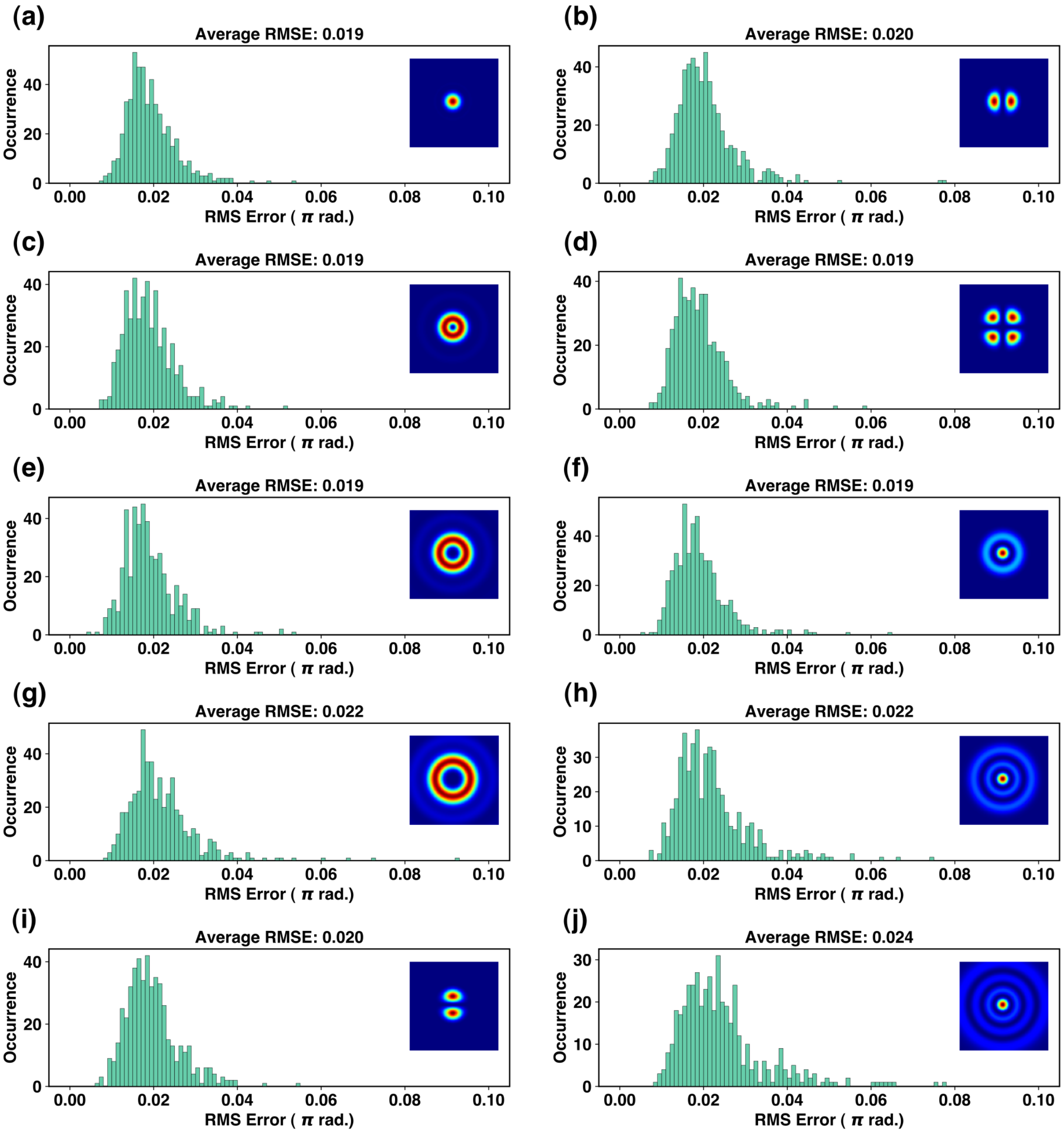

Figure S27: Error distribution per beam profile evaluated on the uniform dataset test set: (a) Gaussian, (b) $HG_{1,0}$, (c) OAM $L = 1$, (d) $HG_{2,2}$, (e) OAM $L = 3$, (f) $LG_{1,0}$, (g) OAM $L = 2$, (h) $LG_{2,0}$, (i) $HG_{0,1}$, (j) $LG_{3,0}$.

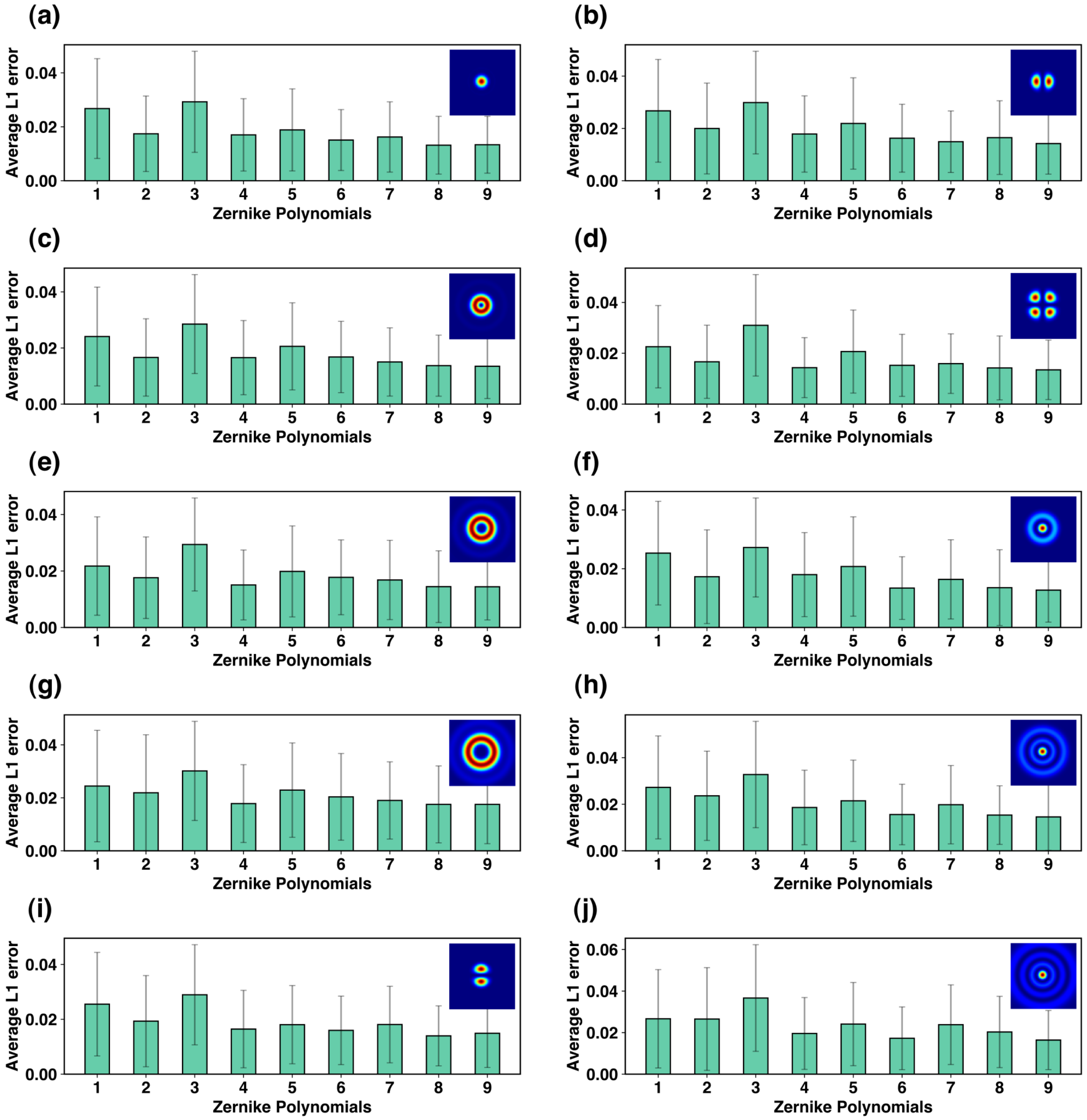

Figure S28: Prediction error for coefficients of each Zernike polynomial for each beam profile: (a) Gaussian, (b) $HG_{1,0}$, (c) OAM $L = 1$, (d) $HG_{2,2}$, (e) OAM $L = 3$, (f) $LG_{1,0}$, (g) OAM $L = 2$, (h) $LG_{2,0}$, (i) $HG_{0,1}$, (j) $LG_{3,0}$. Error bars represent the standard deviation (SD).

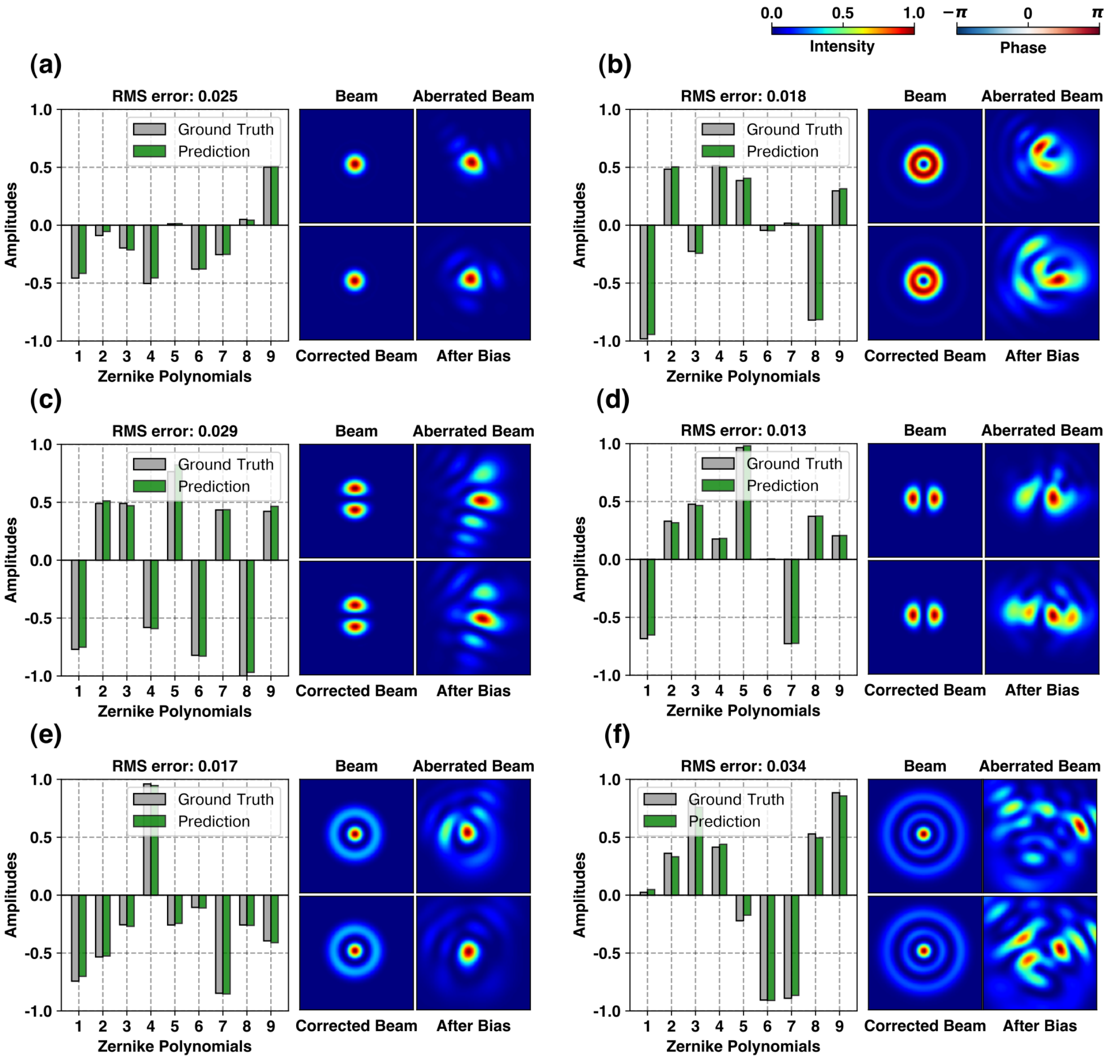

Figure S29: Qualitative examples of aberration detection using our framework on the dataset with uniform coefficients: (a) Gaussian, (b) OAM with $L = 1$, (c) $HG_{1,0}$, (d) $HG_{0,1}$, (e) $LG_{1,0}$, (f) $LG_{2,0}$. For each case, we show the ground-truth and predicted Zernike polynomials, the beam type, the aberrated beam, the intensity distribution after the bias, and the corresponding corrected beam.

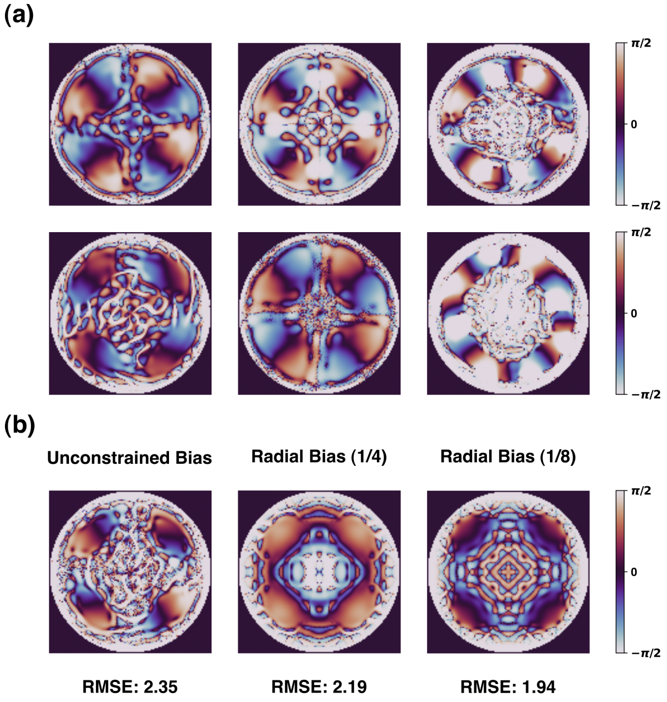

Figure S30: (a) Multiple instances of the trained biases without symmetry constraints evolved into a predominantly even symmetrical patterns, (b) Trained biases with and without symmetry constraints (RSME $10^{-2}\pi$ rad.).

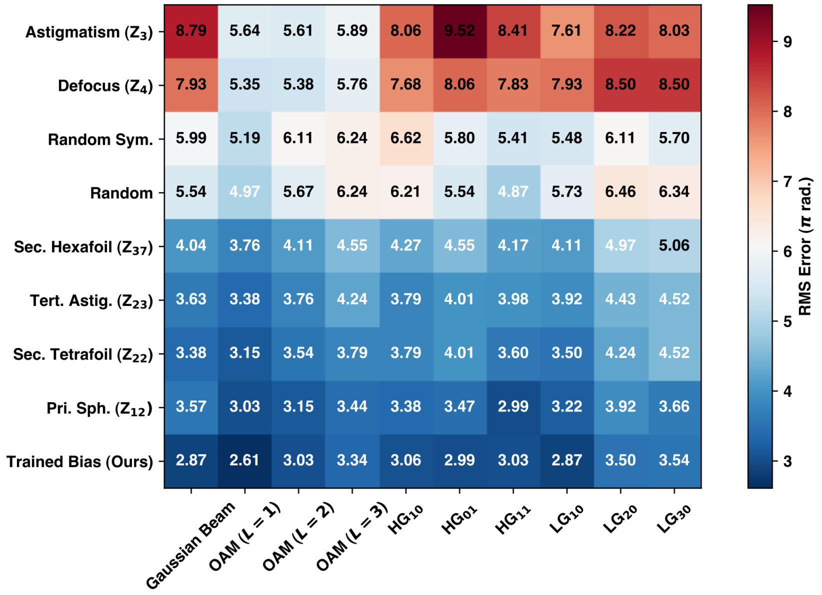

Figure S31: Individual RMSE values computed for each bias and each beam profile (The experiment with no bias was omitted for visualization purposes, Z_{12} value has been corrected).

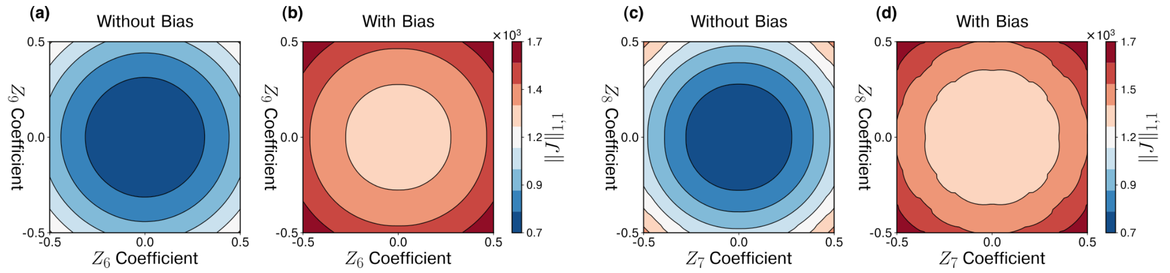

Figure S32: Jacobian norm calculated on 2D cross-sections of the aberration space (Gaussian beam), illustrating the rate of change before and after applying the bias. (a, b) Z_6–Z_9 space, (c, d) Z_7–Z_8 space. The bias increases the Jacobian norm, amplifying the effect of aberrations on intensity.

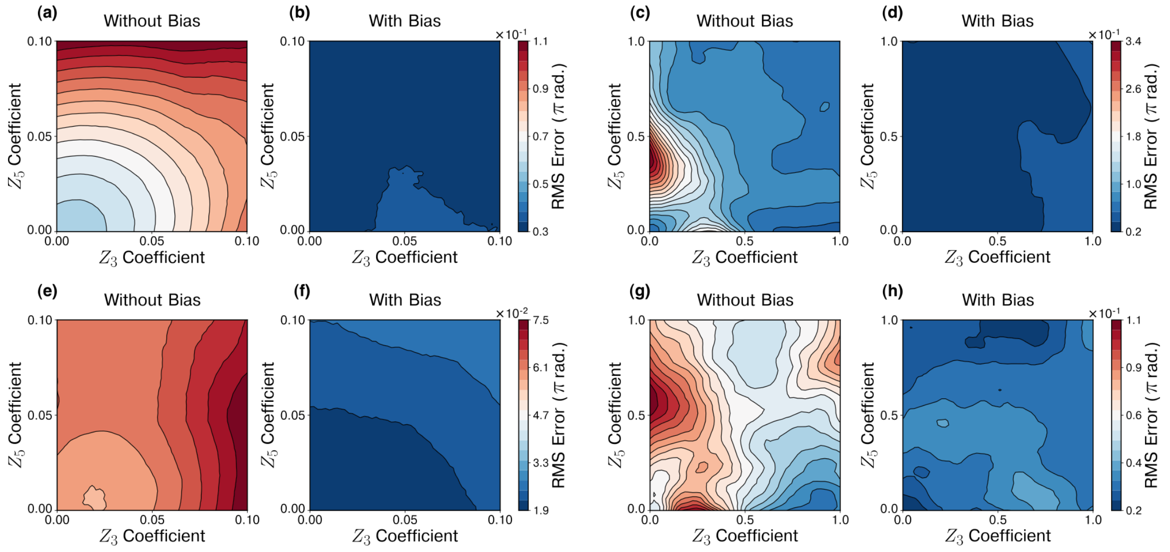

Figure S33: Prediction error of the system evaluated on the 2D cross-section of the Z_3-Z_5 before and after applying the bias. (a, b) Gaussian beam, range 0-0.1, (c, d) Gaussian beam, range 0–1, (e, f) OAM beam ($L = 1$), ranges 0–0.1, (g, h) OAM beam ($L = 1$), ranges 0–1. The bias significantly reduces prediction error and ensures consistent performance across different regions.

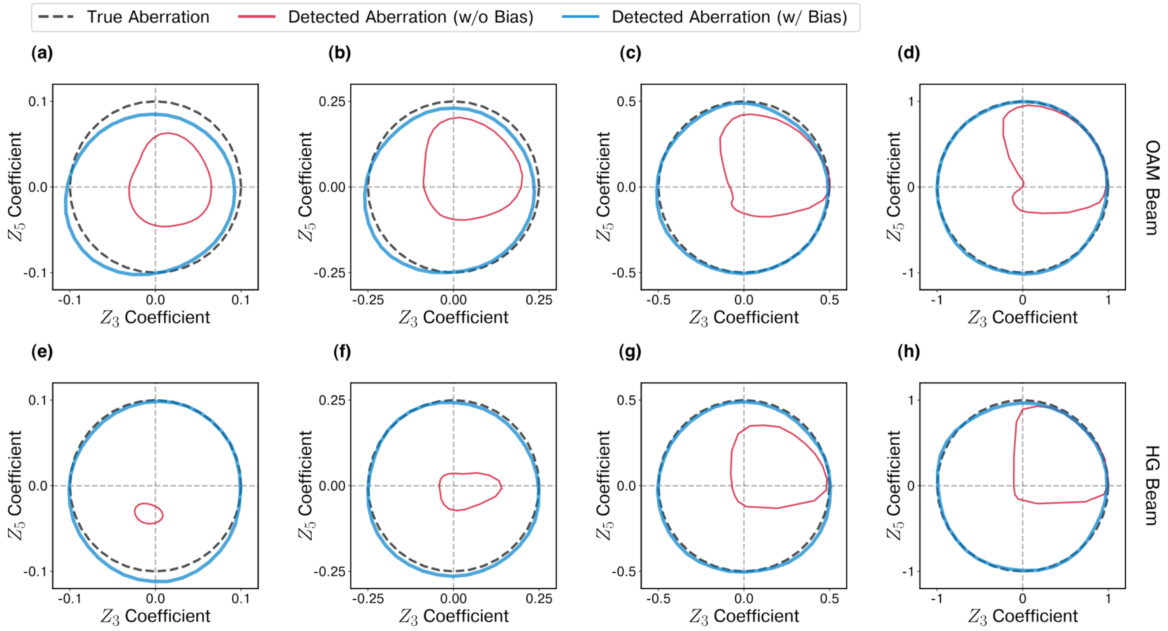

Figure S34: Network accuracy in predicting combinations of two aberration coefficients (Z_3–Z_5), varied continuously between positive and negative values in a circular pattern, with and without bias. Dashed lines indicate the ground truth aberration, solid red lines show predictions without bias, and solid blue lines show predictions with bias. (a–d) OAM beam ($L = 1$) evaluated at aberration magnitudes of 0.1, 0.25, 0.5, and 1. (e–h) HG_{01} beam evaluated at the same magnitudes. The network using the bias accurately detects the aberration sign and maintains consistent, high accuracy across all magnitudes.

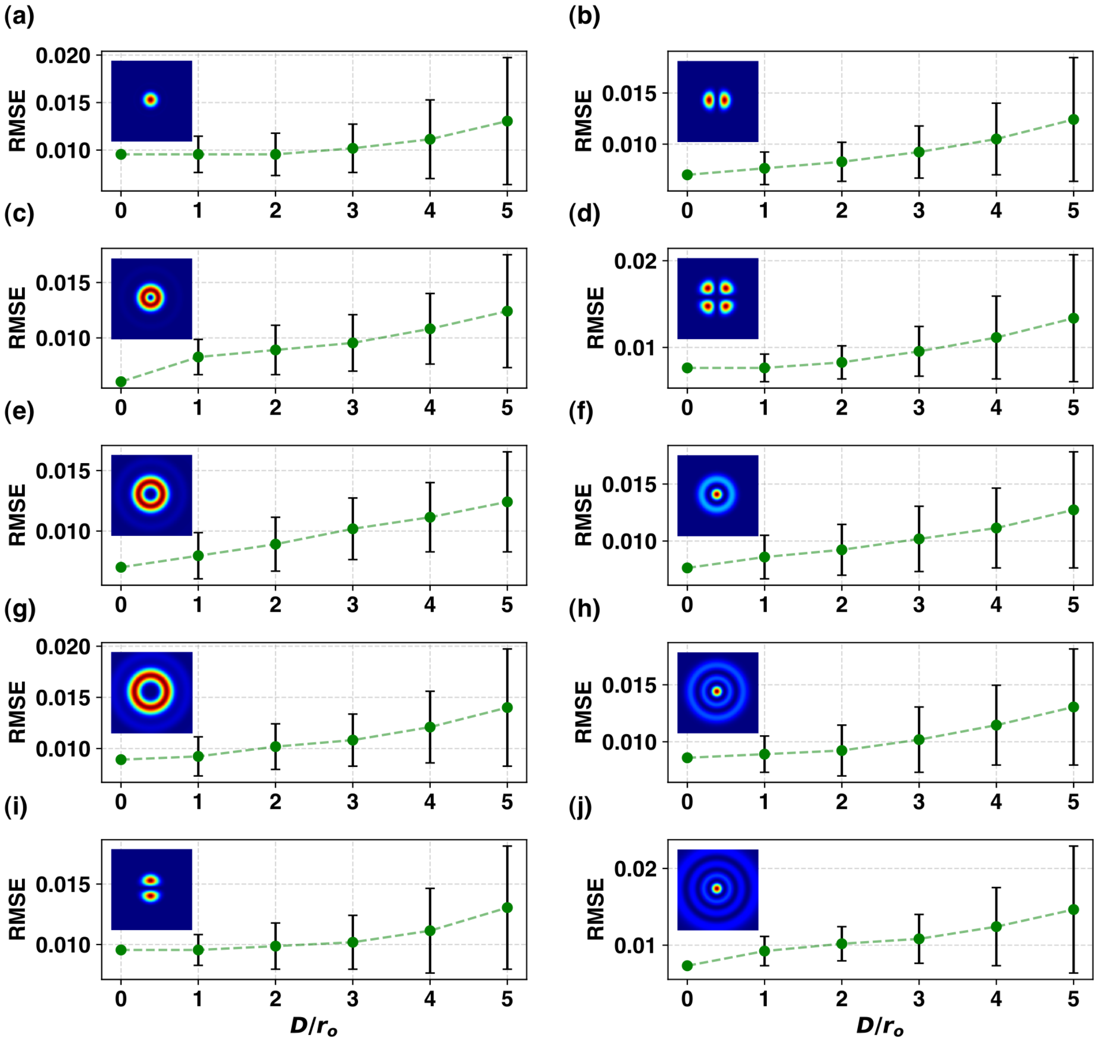

Figure S35: Effect of increasing aberration strength on RMSE across different beam types (aberration expressed in Z_9): (a) Gaussian, (b) $HG_{1,0}$, (c) OAM $L = 1$, (d) $HG_{2,2}$, (e) OAM $L = 3$, (f) $LG_{1,0}$, (g) OAM $L = 2$, (h) $LG_{2,0}$, (i) $HG_{0,1}$, (j) $LG_{3,0}$. Error bars represent the standard deviation (SD).

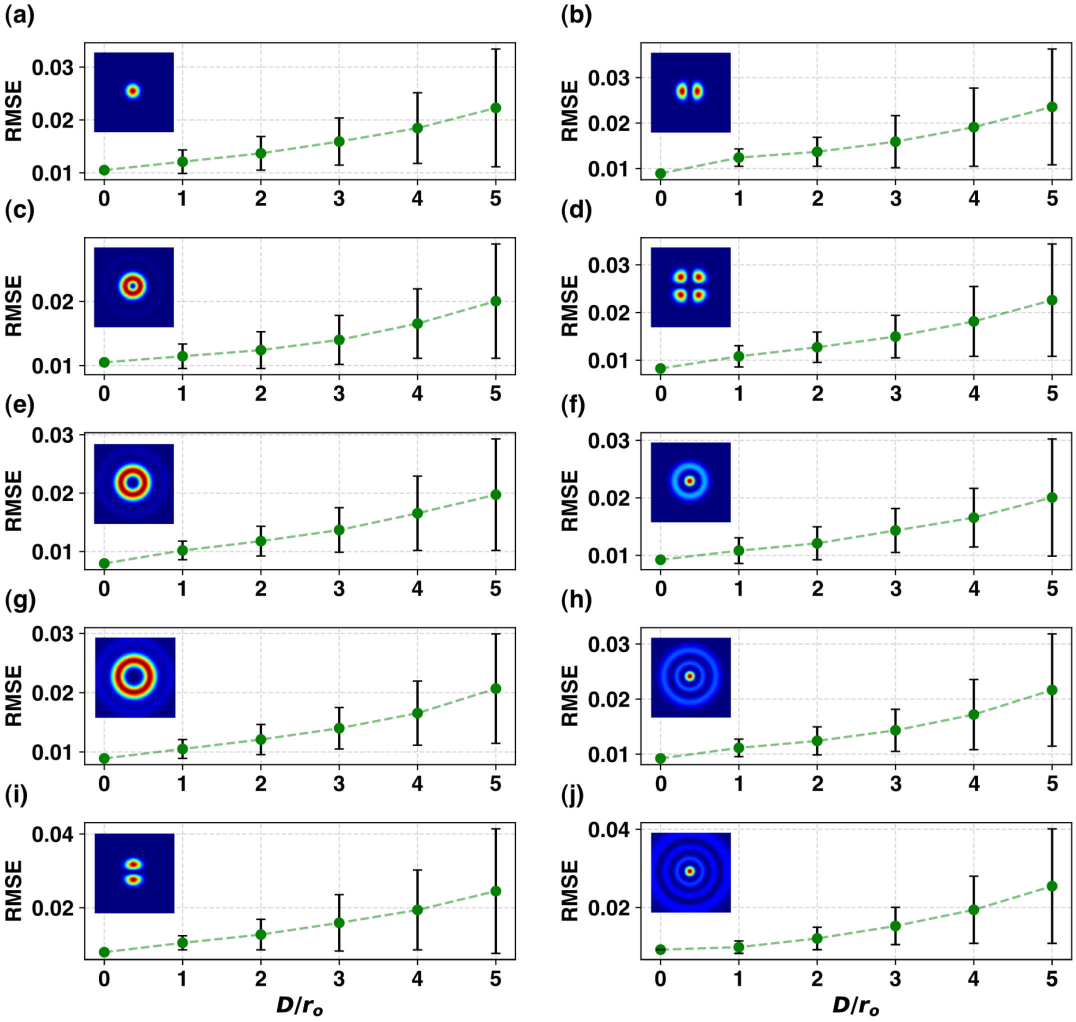

Figure S36: Effect of increasing aberration strength on RMSE across different beam types (aberration expressed in Z_{14}): (a) Gaussian, (b) $\mathrm{HG}_{1,0}$, (c) OAM $L = 1$, (d) $\mathrm{HG}_{2,2}$, (e) OAM $L = 3$, (f) $\mathrm{LG}_{1,0}$, (g) OAM $L = 2$, (h) $\mathrm{LG}_{2,0}$, (i) $\mathrm{HG}_{0,1}$, (j) $\mathrm{LG}_{3,0}$. Error bars represent the standard deviation (SD).

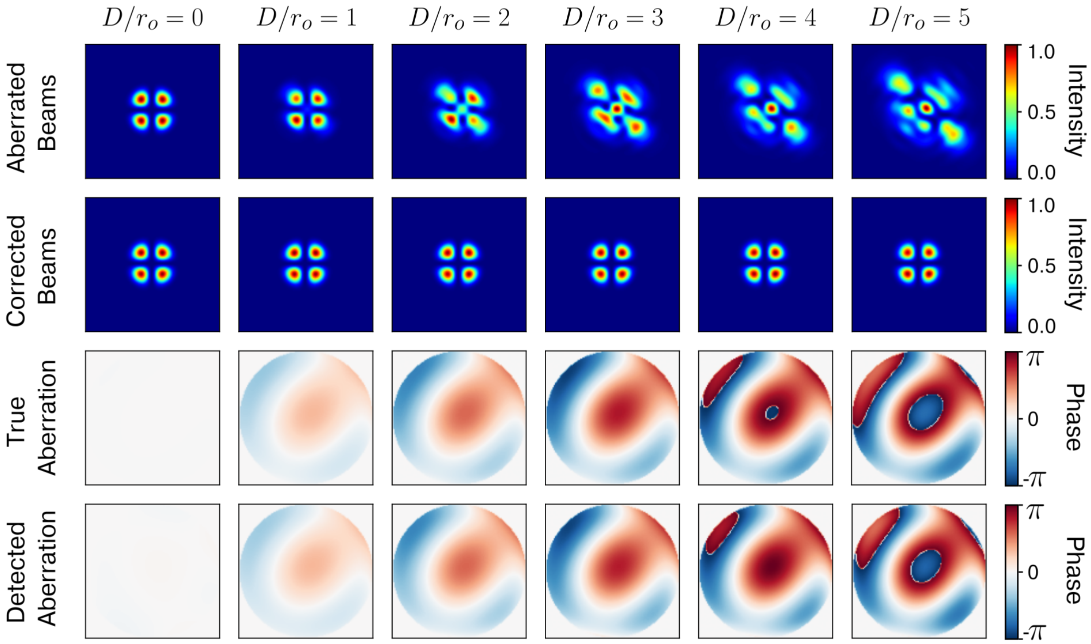

Figure S37: Effect of increasing aberration intensity on the $HG_{2,2}$ mode. The true and detected aberration phase profiles closely match, which is further supported by the corrected beams closely resembling the ideal $HG_{2,2}$ beam across all intensity levels.

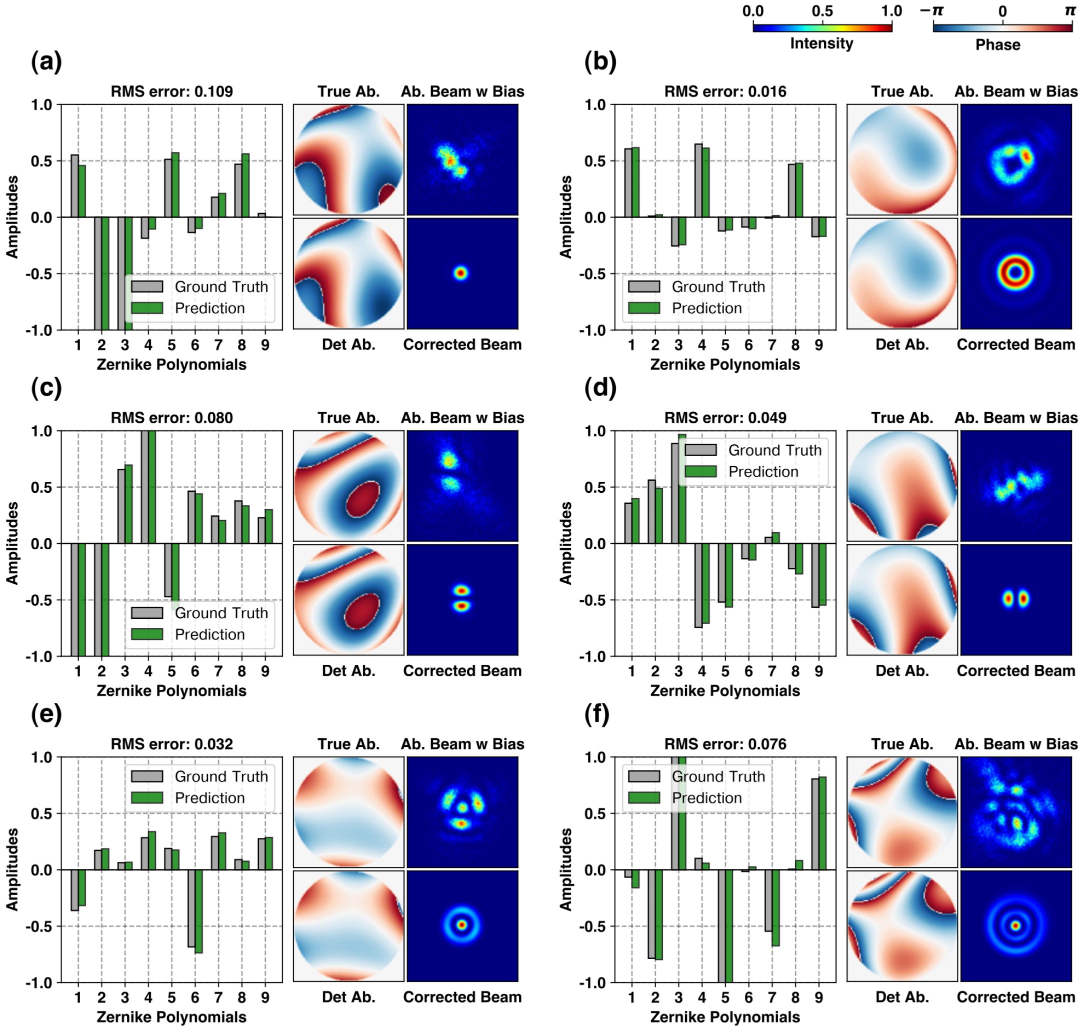

Figure S38: Qualitative examples of aberration detection using our framework in the presence of photon shot noise with 100 average photons received at the brightest pixel in the diffraction limited beam.

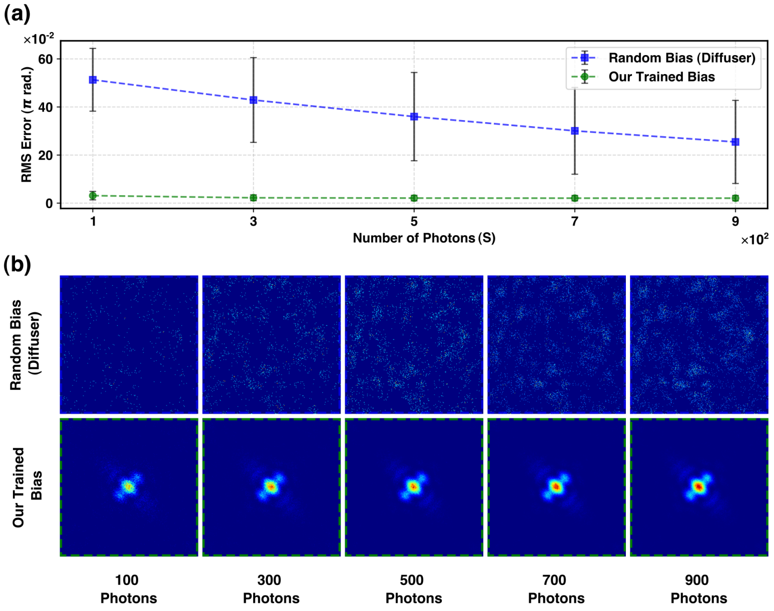

Figure S39: Comparison of trained bias and random bias in the presence of shot noise. (a) RMS error as a function of the number of photons, (b) intensity response of the biases to astigmatism aberration. Error bars represent the standard deviation (SD).

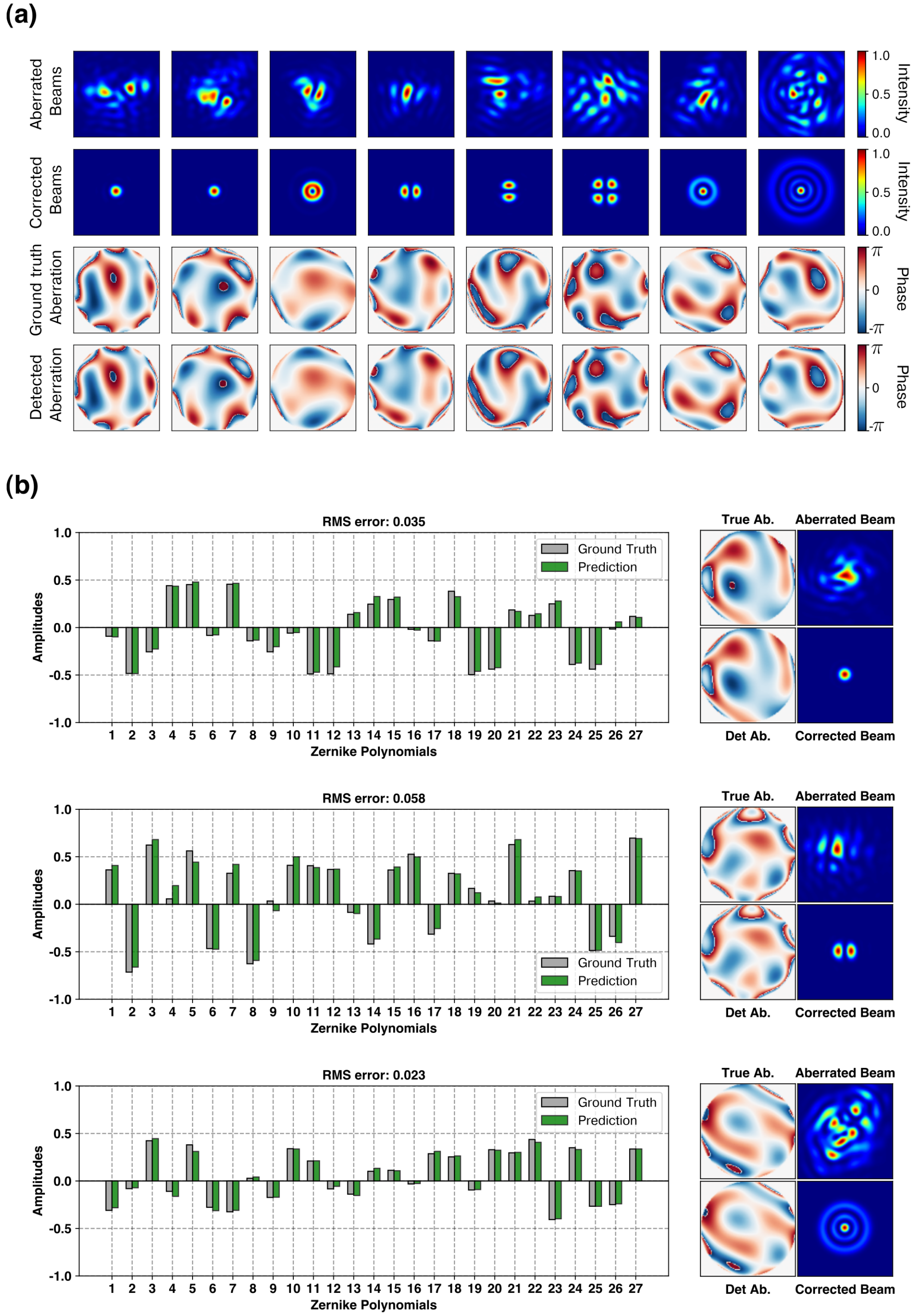

Figure S40: Qualitative examples of aberration detection using our framework on the dataset with aberrations created using the first 27 Zernike polynomials.

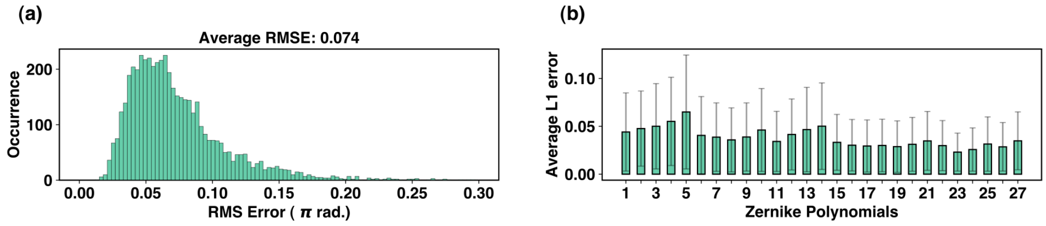

Figure S41: Evaluation results on the Z_{27} dataset. (a) Error distribution on the test set for the dataset with aberrations created using the first 27 Zernike polynomials, (b) average L1 error for each Zernike polynomial on the test set. Error bars represent the standard deviation (SD).

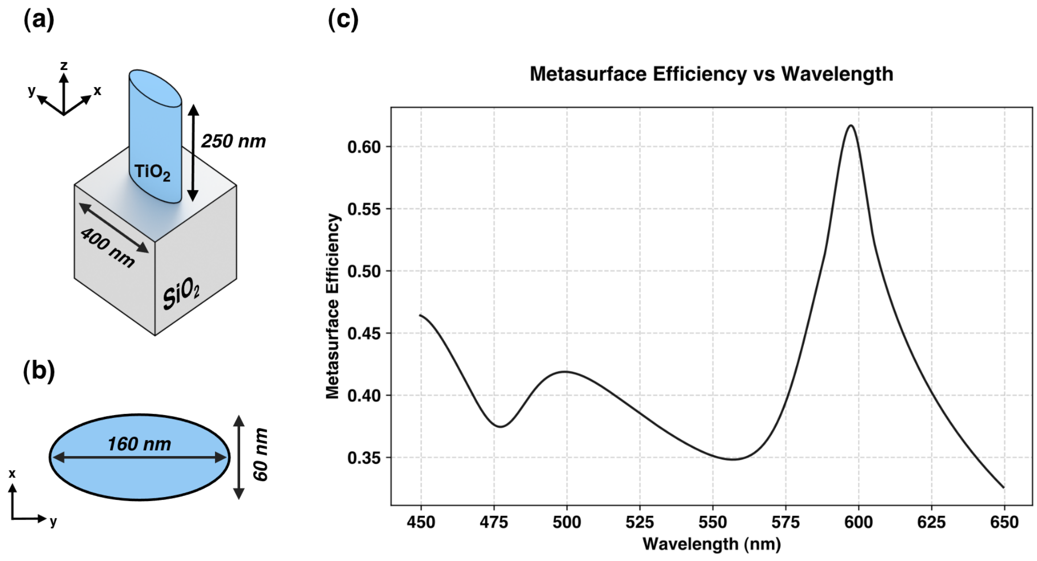

Figure S42: (a) Unit cell of the metasurface (tilted view), (b) Unit cell of the metasurface (top view), (c) efficiency vs wavelength.

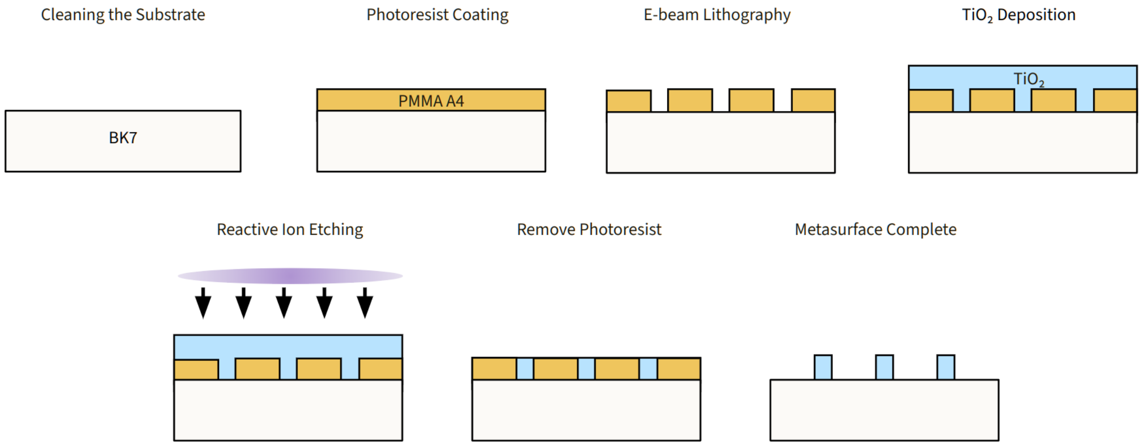

Figure S43: Metasurface fabrication process. A BK7 glass substrate was first cleaned using acetone and isopropyl alcohol (IPA) with sonication, followed by rinsing with deionized (DI) water to ensure a contaminant-free surface. A 260 nm layer of PMMA A4 electron beam resist was then spin-coated onto the substrate and baked at 180 °C for 1 minute. Electron beam lithography was performed to define the desired metasurface pattern, followed by development. A 150 nm thick TiO_2 layer was subsequently deposited via atomic layer deposition (ALD) at 90 °C. The low-temperature deposition ensures the formation of amorphous TiO_2 and prevents contamination of the ALD chamber. Reactive ion etching (RIE) was then used to remove excess TiO_2, employing a gas mixture of BCl_3 and Cl_2 with an etch rate of approximately 20 nm/min. Finally, the remaining photoresist was removed through a liftoff process, completing the fabrication of the TiO_2 metasurface.

9 Inserting aberration correction system into a practical optical setup

To integrate this system into a practical configuration, only minimal changes are needed. The primary modification is the entry point of the laser into the imaging path. The key idea is to guarantee that the beam illuminating the specimen (for example, biological tissue) is itself not aberrated. A relevant example comes from STED microscopy, where donut-shaped beams have been widely used. In many implementations, by the time the donut beam reaches the specimen, the optical system has already introduced aberrations. Our approach ensures that the beam reaching the specimen is pre-corrected. Experimentally, this is achieved by taking a portion of the focal-plane image through an unequal beam splitter (e.g., R:T = 8:92), feeding it to the SLM for pre-compensation, and ensuring that the beam transmitted toward the specimen is corrected. To guarantee that the beam remains pure, we collect approximately 8% of the beam using an unequal beam splitter and send it into our aberration-correction system. This system consists of a focusing lens and the metasurface used to lift the FPA ambiguity. The captured intensity image on the camera is then used to infer the aberration, and the corresponding conjugate phase is applied by the SLM. In this way, the SLM pre-compensates the optical system for the aberrations that would otherwise distort the OAM mode before it reaches the DUT. This pre-compensation ensures that a clean, well-defined OAM beam interacts with the sample, allowing us to quantify the effects of both the sign and magnitude of the OAM mode on the DUT. In practice, the procedure requires only a single intensity image, and the aberration-correction algorithm supplies the necessary corrective phase. As a result, we can observe both the corrected beam (for aberration detection) and the aberration-free beam (for evaluating the DUT response) on the camera.

10 Shack-Hartmann Comparison

To provide a concrete comparison and demonstrate how our approach can be more robust than traditional wavefront sensors, we implemented a Shack-Hartmann wavefront sensor (SHWFS) numerically and compared its performance with our method. We used a 20x20 lenslet array mask, which is adequate for the spatial frequency of our dataset. Additionally, increasing the number of lenslets did not enhance accuracy, and only increased the computational burden. For each lenslet, we computed the intensity response, determined the local slope from the centroid displacement, and reconstructed Zernike coefficients using a least-squares method [11].

We further evaluated SHWFS on the Kolmogorov dataset ($Z_9, D/r_o = 5$) and compared it to our method, both with and without noise. The evaluation was limited to only the Gaussian beam, as structured beams lead to significantly larger errors in the presence of noise for SHWFS. In the case without noise, the RMS error obtained from our method is $2.3 \times 10^{-2}\pi$ radians, while the error associated with the Shack-Hartmann Wavefront Sensor (SHWFS) is $2.8 \times 10^{-2}\pi$ radians which is still higher even under ideal conditions. In contrast, when mild photon shot noise is introduced into the system, the error for SHWFS increases significantly to $27.8 \times 10^{-2}\pi$ radians. Meanwhile, our method maintains a consistent error of $2.5 \times 10^{-2}\pi$ radians. Several examples of the detection and

correction process are presented in Figure S44. The results indicate that the mild noise disrupted the performance of the SHWS. While the polarity of the Zernike modes was identified correctly, the magnitudes of each mode could not be accurately detected due to the noise. This limitation arises because SHWFS reconstruction depends on the precise estimation of spot centroids, and its performance is significantly affected by photon shot noise, camera read noise, and other fluctuations in intensity. This makes SHWFS particularly non-robust in low-light conditions, further limiting its applicability in many practical scenarios. In contrast, our network demonstrates consistent performance even in noisy conditions. The APN does not depend on the intensities of individual pixels; instead, it focuses on the local correlations between each pixel and its neighbors. As a result, the reconstruction is more robust to various types of noise.

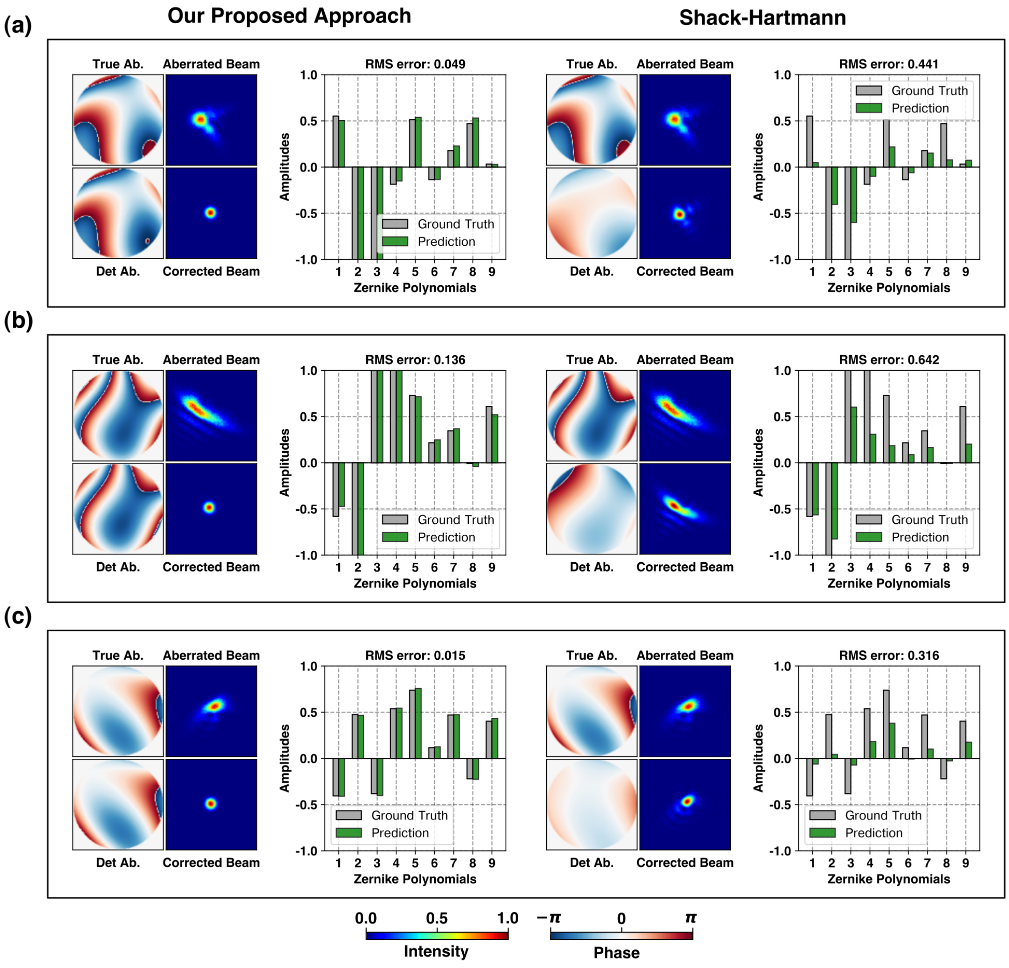

Figure S44: Instances illustrating the detection and correction process, comparing our method to the simulated Shack-Hartmann approach in the presence of noise.

11 Overview of the Beams

Here we summarize the key beam solutions of the paraxial wave equation, describing the propagation of Gaussian beams in free space, including Laguerre-Gaussian beams, their specialized form as Orbital Angular Momentum (OAM) beams, and Hermite-Gaussian beams.

11.1 Gaussian Beam

$$\begin{aligned} E_0(x, y, z) =& E_0 \frac{w_0}{w(z)} \exp\left(-\frac{x^2+y^2}{w^2(z)}\right) \times \exp\left(-i\frac{k(x^2+y^2)}{2R(z)}\right) \\ & \times \exp(i\psi(z)) \exp(-ikz). \end{aligned} \tag{33}$$

- **Beam waist (w_0):** The minimum beam radius at the focus ($z = 0$). At this radius, the electric field amplitude drops to $1/e$ of its on-axis value.
- **Rayleigh range (z_R):** The distance over which the beam significantly diverges, defined as:

  $$z_R = \frac{\pi w_0^2}{\lambda}.$$

  At $z = z_R$, the beam radius expands to $\sqrt{2}w_0$.
- **Beam radius ($w(z)$):** Describes the beam expansion at distance z:

  $$w(z) = w_0\sqrt{1 + \left(\frac{z}{z_R}\right)^2}.$$

- **Radius of curvature ($R(z)$):** The wavefront curvature radius at position z:

  $$R(z) = z\left[1 + \left(\frac{z_R}{z}\right)^2\right].$$

- **Gouy phase ($\psi(z)$):** Longitudinal phase shift acquired through propagation:

  $$\psi(z) = \arctan\left(\frac{z}{z_R}\right).$$

These parameters collectively describe beam propagation, divergence, and phase accumulation.

11.2 Laguerre-Gaussian (LG) Modes and OAM

Laguerre-Gaussian modes form a set of cylindrically symmetric solutions characterized by radial index p and azimuthal index ℓ. The electric field amplitude in cylindrical coordinates (r, θ, z) is expressed as:

$$E_{p,\ell}(r,\theta,z) = E_0 \frac{w_0}{w(z)} \left(\frac{r\sqrt{2}}{w(z)}\right)^{|\ell|} L_p^{|\ell|}\left(\frac{2r^2}{w^2(z)}\right) \times \exp\left(-\frac{r^2}{w^2(z)}\right) \exp\left(-i\frac{kr^2}{2R(z)}\right) \times \exp(i\ell\theta)\exp(i\psi(z))\exp(-ikz), \tag{34}$$

where $L_p^{|\ell|}$ are generalized Laguerre polynomials.

11.2.1 Orbital Angular Momentum (OAM) Beams

LG modes with $p = 0$ ($\mathrm{LG}_{0,\ell}$) carry orbital angular momentum (OAM). They exhibit a doughnut-shaped intensity profile and a helical wavefront:

$$E_{0,\ell}(r,\theta,z) = E_0 \frac{w_0}{w(z)} \left(\frac{r\sqrt{2}}{w(z)}\right)^{|\ell|} \times \exp\left(-\frac{r^2}{w^2(z)}\right) \exp\left(-i\frac{kr^2}{2R(z)}\right) \times \exp(i\ell\theta)\exp(i\psi(z))\exp(-ikz). \tag{35}$$

Each photon in this beam carries OAM equal to $\ell\hbar$.

11.3 Hermite-Gaussian (HG) Modes

Hermite-Gaussian modes are rectangularly symmetric beam solutions characterized by indices l and m denoting the node number along the x and y axes. The electric field amplitude in Cartesian coordinates (x, y, z) is:

$$E_{l,m}(x,y,z) = E_0 \frac{w_0}{w(z)} H_l\left(\frac{\sqrt{2}x}{w(z)}\right) H_m\left(\frac{\sqrt{2}y}{w(z)}\right) \times \exp\left(-\frac{x^2+y^2}{w^2(z)}\right) \exp\left(-i\frac{k(x^2+y^2)}{2R(z)}\right) \times \exp\left(i\psi_{l,m}(z)\right)\exp(-ikz), \tag{36}$$

with H_l and H_m being Hermite polynomials. Higher-order HG modes have more complex intensity patterns and accumulate Gouy phase faster than the fundamental mode, following:

$$\psi_{l,m}(z) = (l+m+1)\arctan\left(\frac{z}{z_R}\right).$$

HG modes form a complete basis set for describing rectangular-symmetric paraxial beams. They naturally represent the transverse eigenmodes of laser resonators with rectangular geometry.

References

1. Hinton GE and Zemel R. Autoencoders, Minimum Description Length and Helmholtz Free Energy. In: *Advances in Neural Information Processing Systems*. Ed. by Cowan J, Tesauro G, and Alspector J. Vol. 6. Morgan-Kaufmann, 1993.
2. Hu Y, Wang Z, Wang X, et al. Efficient full-path optical calculation of scalar and vector diffraction using the Bluestein method. Light: Science & Applications 2020;9:119.
3. He K, Zhang X, Ren S, and Sun J. Deep Residual Learning for Image Recognition. In: *2016 IEEE Conference on Computer Vision and Pattern Recognition (CVPR)*. 2016:770–8. DOI: 10.1109/CVPR.2016.90.
4. Akiba T, Sano S, Yanase T, Ohta T, and Koyama M. Optuna: A Next-generation Hyperparameter Optimization Framework. In: *Proceedings of the 25th ACM SIGKDD International Conference on Knowledge Discovery and Data Mining*. 2019.
5. McGlamery BL. Computer Simulation Studies Of Compensation Of Turbulence Degraded Images. In: *Image Processing*. Ed. by Urbach JC. Vol. 0074. International Society for Optics and Photonics. SPIE, 1976:225–33. DOI: 10.1117/12.954724. URL: https://doi.org/10.1117/12.954724.
6. Roddier N. Atmospheric wavefront simulation using Zernike polynomials. Optical Engineering 1990;29:1174–80.
7. Zhang R, Isola P, Efros AA, Shechtman E, and Wang O. The Unreasonable Effectiveness of Deep Features as a Perceptual Metric. In: *CVPR*. 2018.
8. Janesick JR. Scientific charge-coupled devices. Press Monographs. Bellingham, WA: SPIE Press, 2001.
9. Ronneberger O, Fischer P, and Brox T. U-Net: Convolutional Networks for Biomedical Image Segmentation. In: *Medical Image Computing and Computer-Assisted Intervention – MICCAI 2015*. Ed. by Navab N, Hornegger J, Wells WM, and Frangi AF. Cham: Springer International Publishing, 2015:234–41.
10. Kingma DP and Ba J. Adam: A Method for Stochastic Optimization. In: *ICLR (Poster)*. Ed. by Bengio Y and LeCun Y. 2015. URL: http://dblp.uni-trier.de/db/conf/iclr/iclr2015.html#KingmaB14.
11. Cubalchini R. Modal wave-front estimation from phase derivative measurements. Journal of the Optical Society of America 1979;69:972.
