# Supplementary material for: An end-to-end hybrid deep-learning approach for single-shot wavefront sensing and correction
Source: Nat Commun. 2026 May 12;17:6340. doi: 10.1038/s41467-026-72364-1 (PMC13377082; doi:10.1038/s41467-026-72364-1)
Supplement: Supplementary file 2 — Transparent Peer Review file [file 41467_2026_72364_MOESM2_ESM.pdf]

nature portfolio

Peer Review File

An end-to-end hybrid deep-learning approach for single-shot wavefront sensing and correction

Corresponding Author: Professor Abdoulaye Ndao

This file contains all reviewer reports in order by version, followed by all author rebuttals in order by version.

Version 0:

Reviewer comments:

Reviewer #1

(Remarks to the Author)
The authors propose a deep learning approach for aberration detection.
My recommendation would be to make the abstract more concise and to better reflect what is actually achieved in the work. Currently, the abstract rises rather too high expectations which are not really met in the work.
The main limitations of the approach as demonstrated are that only very easy aberrations are corrected which might equally well be corrected with a simple intensity based approach, maybe also using deep learning.
There are many approaches with phase elements in the detection path, and these should be cited more carefully. Overall, the approach seems rather more complicate than existing ones with better performance.
Line 202: please remove „transformative“

Reviewer #2

(Remarks to the Author)
This paper presents a new method for optical wavefront sensing which jointly learns a diffractive phase mask to encode the wavefront into intensity and a reconstruction deep learning model which maps the intensity back to an estimate of the wavefront. The key insight of the paper is that one of the ambiguities of the standard phase to intensity mapping via far field diffraction is that that phases with negative or positive even components map to the same intensity. Thus by inserting a mask of sufficient complexity, this ambiguity is no longer observable within the realm of reasonably observable phases. By learning this phase mask jointly with a intensity to phase mapping, the authors are able to simultaneously improve the injectivity of the phase to intensity mapping and the ability to invert it. They term this strategy Aberration Prediction Network (APN) and demonstrate its viability in simulation and experimentally.

To train APN they created simulated pairs of phase maps (parametrized with low order Zernike coefficients < 4th order) and intensity distributions (via bluestein diffraction method), and trained the model to predict the coefficients given the intensity. They then tested the model on held out simulation data of the same type (zernike < 4th order) both uniformly sampled and correlated according to Kolmogorov turbulence covariances. Experimentally, they ran similar experiments by expressing the true aberrations on an SLM, recording with either an SLM displaying the learned mask or a custom made metasurface. They are able to then apply the conjugate estimate phase to retreive the unaberrated wavefront. They run these experiments over different beam types, a 200nm range of visible wavelengths, and up to 1pi radian of RMS aberration strength

Overall the performance of their method is impressive. Even in experiments, they show relatively high accuracy in estimating low order aberrations to the point where correcting them works well. Their methodology is also nicely motivated as they show the existence of one possible phase to intensity ambiguity and describe how to fix it. Their use of learning seems rigorous and effective, accounting for hyperparameters and model mismatch (via augmentation). They also do a great job of validating/ablating various components of their method including showing the state of ambiguity before and after the bias as well as comparing to fixed biases. I think the overall approach is novel both in the sense that it effectively uses end-to-end learning for wavefront sensing and because it recognizes (and demonstrably fixes) the even phase component ambiguity.

While, I think this approach could have great value to the community I do think there are a few important points to address, additional suggested experiments, and other clarifying questions/suggestions, which I put below. I apologize in advance for

any mistakes or misreads I may have and am looking forward to a productive exchange with the intent of improving this paper as much as possible!

Before getting to specifics, I want to summarize that the core limitation of this paper is that the authors never display this system in an actual application setup (e.g., imaging, metrology, etc). Thus claims of its use as a universal aberration corrector are a bit of a reach without further validation. Instead what the authors have shown is a novel method for wavefront sensing, which, if further applied could be used in the context of aberration correction. Suggestions to that end are put below including a few main points: language of the paper (including the title) should reflect this limited scope, the method appears to not be evaluated in a realistic SNR regime (its unclear what the laser power is) and is never directly evaluated on aberrations outside of those generated by the first 14 Zernike coefficients which the model is trained on.

Comments:

- Why are showing multiple beam types outside of Gaussian useful? Where do they come up in the applications you mention
- Maybe provide an example or two since readers (including myself) may not be familiar with why they are important

- I feel that the writing throughout borders on grandiose and, at times, overclaims. I suggest going through the writing and shifting the focus away from how it's a fundamental methodological breakthrough or how it will perform amazingly in imaging applications to instead in what ways your technique is quantifiably better than existing wavefront sensing, and how that perhaps could improve certain applications that use wavefront sensing/correction. At its core this is work uses a typical direct wavefront sensing setup but jointly learns the encoding element and decoding algorithm. It then shows that such an approach works in a proof-of-concept setup.
- The title for example 'One image, all aberrations' and 'universal framework for aberration correction' feels like a pretty strong overclaim. Indeed your method only demonstrates correction over the partial visible range, lower order zernike aberrations which are monochromatic (i.e., not chromatic aberrations) and spatially-invariant. Moreover, correction is does not seem like the main contribution of the paper, but rather wavefront sensing (no new methodology for correction is presented). Finally, you don't ever demonstrate it in an actual imaging context. I would tone down this title and make it more scientifically informative (e.g., "A end-to-end hybrid deep-learning approach for single-shot wavefront sensing") or something of the sort where the focus is on your contribution (single-shot wavefront sensing) and methodology (end-to-end learning of a phase bias and prediction network).
- Another example is something like "The proposed framework will enable cutting-edge imaging modalities, including high-resolution visualization of tissues through multilayered cellular structures with varying refractive indices, thereby delivering unparalleled spatial resolution and facilitating precise quantitative analysis within complex biological environments crucial for biomedical diagnostics and research." is a pretty large overclaim given that you never did any imaging experiments. It's fine to suggest future applications of your method and why it might the improve status quo but to suggest that it will create 'unparalleled resolution' is a large leap in logic.
- "Here, we report a unified, single-shot wavefront correction framework that departs fundamentally from conventional approaches." or "In summary, we present a transformative single-shot wavefront correction framework that fundamentally redefines conventional paradigms.". The claim of departing fundamentally for conventional approaches is unsubstantiated; it is the conventional setup of many wavefront sensing techniques to encode the wavefront via some optic (diffractive, lenslet, etc) and then map the resulting intensity back to phase via computation. The proposed approach builds on this line of thinking by jointly learning mask and reconstruction algorithm.
- "Our framework establishes a scalable foundation for real-time aberration correction in advanced optical systems, while simultaneously offering fundamental insights into the origin and behavior of optical aberrations". I don't think there is any evidence of this paper to support new insights into the "origin and behavior of optical aberrations". Perhaps you can claim insight into the nature of wavefront sensing ambiguity (e.g., via the FPA argument) but not aberrations themselves.

- What about dynamic aberrations and/or shift-varying aberrations; these are common in biological tissue and atmospheric turbulence. It is out of scope but can you at least put a discussion about how this method could extend to compensate for it? Perhaps it is as simple as just calibrating a per field-of-view point coefficient set and then sequentially applying correction for a point scanning system? But what if it's not a point scanning system?
- For example: https://opg.optica.org/optica/fulltext.cfm?uri=optica-11-2-297

- I feel that the related work section could talk about similar methods that also use a nontraditional element (like a diffuser) as an encoder and then do computational reconstruction (e.g., https://opg.optica.org/ol/abstract.cfm?uri=ol-42-24-5117). They often share properties of being inexpensive, compact, broadband, etc.

- Why not compare to a shack-hartmann wavefront sensor?
- I don't think it's necessary to actually do experimentally, but at least explain more specifically in what ways your method is better (speed, accuracy, etc). You mention a bit in the related work but it'd be nice for a practitioner to understand what benefits they'd get by switching to your system by comparing your results with the expected results of a traditional wavefront sensing system like shack-hartmann, which for many is the status quo.

- Is the network explicitly trained with different wavelengths or only a single wavelength? That is, when you generate your training data with Bluestein's method which wavelength(s) do you use? Is it possible that given your imaging geometry, wavelength simply does not have enough impact which is why you observe the broadband behavior?

- What about varying noise levels? I'm worried that a good chunk of success of this method is due to have a ton of light at the wavefront sensor which is often not this case in applications like astronomy and microscopy.

- To that end please discuss the power of the laser you used (I can't find it anywhere) and talk about the amount of light hitting your wavefront sensor and compare it to a standard level as would be seen in a adaptive optics context. If possible, I would love to see a small experiment showing the performance of the network at low signal levels (this can even be done in simulation)

- In line 59 and in the supplement proof of focal plane ambiguity can you explain the conjugate inverse of phi? phi is a real-valued function and thus it's conjugate is just itself? Do you intend to say the conjugate of the reflected pupil function? If so, be specific because I think the claim that conj(phi(-x)) = -(phi_odd(-x) + phi_even(-x)) is not true

- I may be mistaken but it seems like this condition of even symmetric bias is not a necessary condition. Any phase that has a non-zero even component (i.e., is not odd) should also accomplish the same outcome, no? The odd component won't affect anything but the even component should help disrupt the phase ambiguity. If this is true, you should be explicit about why you choose an even only component when any bias technically suffices.
- On that note, what happens if you optimize an arbitrary bias (instead of a purely even one), will it do better because it has more degrees of freedom or worse because it is inefficient in the sense that only the even component contributes to FPA. One could argue that changing the odd component could still help with other forms of ambiguity that prevent total injectivity.

- It would be nice to summarize the ambiguity correction into a single mathematical statement like "FPA is removed by any bias whose even component does not lie in the span of the finite set of even Zernike polynomials producible by the system" which you can prove quite easily. I think the discretization is nice for intuition but this statement I believe should hold in hilbert space as well.

- Another small confusion: you say you imposed radial symmetry constraints an only learn a 1/8th wedge. Your function doesn't appear to be radially symmetric (i.e., invariant to rotation) unless I'm using that term incorrectly. Moreover, to achieve a purely even function isn't it sufficient to just learn a quarter? Why do you pick 1/8?

- For the baseline biases, the zernike modes are nice because they validate that being in the span of the producible zernike modes fails to fix FPA. However it would be nice to also see a high order, even zernike mode beyond what the network can produce as another baseline bias. It satisfies the necessary condition to fix FPA and should have potentially less scattering than the random mask. I think have 3 lower order zernikes is redundant and fails to adequately capture reasonable baseline options.
- I'm curious how you implemented the random bias baseline. I imagine how smooth the random profile is may have great effect on its performance. Less smooth likely causes aberrations to be more distinguishable but also scatters more. Indeed I believe a maximally random mask should work quite well because the resulting speckle will be extremely sensitive to small phase changes. What if you make an even random mask (i.e., randomize 1/4 of the mask and then reflect to create the rest).

- How many coefficients does the model predict during inference? Is this number fixed or adaptable (do you have to retrain the model to accommodate more or less coefficients? If so did you retrain the model multiple times for different coefficient ranges?
- This seems like a potential practical drawback and perhaps should be discussed

- To get a better sense of how much the model helps, could you simply run Gerchberg-Saxton or a gradient-based approach that directly regressing coefficients to best match the measured intensity through your learned bias? It doesn't need to be crazy comprehensive but I'm curious to see it on maybe a small subset of the experimental/simulated data. It would also give a sense of how well the bias removes ambiguity (and not just the model). If there is a strong reason not to do this, than feel free to bring it up.
- I could argue that the classical approach could be more flexible and generalizable because you can always easily select exactly which coefficients you want to fit and you aren't worried about some unpredictable/uninterpretable model failure. Moreover, it is likely more agnostic to different imaging systems/parameters or explicitly incorporates them into the solution.

- The experimental calibration seems like an important step to get good results, can you talk about which steps were exclusively to ensure that you could properly measure error against your SLM-induced aberrations and which steps are critical to perform if you were to insert such a system into a practical imaging setup (e.g., adaptive optics).
- In this discussion can you also speak more to the wavelength specific calibration you did to ensure broadband performance? Would it be challenging to do this calibration in a real adaptive optics context?

- In fig s21 is there a reason the 3rd zernike coefficient consistently has the highest error?

- Is there a reason why you think the error on the held-out Kolmogorov coefficients is lower than on the uniform set (which you trained on)?
- Is it because the Kolmogorov set tends to feature 'easier' modes biased more toward lower orders?

- How would the model perform on more unstructured phase (e.g., smoothed random phase) not generated directly from low order zernike polynomials? Would it naturally find the best fitting coefficients or explode? Exploring this potential failure mode is of interest.
- I noticed you did a robustness test of zernike modes up to 55 but it's not clear to me if you actually tested the accuracy of predicting/correcting such modes with your model. If you didn't do this then explain why not.
- I would prefer you do the higher-order experiment and report the outcome. Given that the model appears to have a restricted set of coefficients it can learn, it's important to understand it's behavior for potentially higher modes (beyond what

it’s trained on) which could easily arise in an experimental setting. Even a few examples of what the model does when faced with something outside of its representational capabilities would be illuminating.

- I don’t have much experience with metasurfaces and cannot adequately evaluate that part of the manuscript.
- I noticed you only did the experiment at 600nm with the metasurface; is a potential limitation of using such a mask that it is not achromatic? If so, can you mention this, as it softens the claim of the system being ‘broadband’.

- One concern I have is how adaptable your model would be if I were to just place it into a new system with different imaging optics and parameters (NA, power, etc). Would you have retrain the model? Use a different bias? If so this provides additional overhead for a user and requires them to sufficiently understand their system and be able to calibrate it such that training data matches experimental data. I would love for a discussion on this potential limitation and how you envision your system being deployed as a practical wavefront sensor

Version 1:

Reviewer comments:

Reviewer #2

(Remarks to the Author)
The authors adequately addressed my concerns and added compelling new evidence to support their claims. I commend the authors for their receptive and comprehensive response. I support publication of this paper.

(Remarks on code availability)
The code seems well organized and documented, and has adequate instructions to theoretically recreate results. That being said I do feel the authors should include a minimal working example that can be run with very little setup and timing. A suggestion is to include a pretrained model and small evaluation dataset with a jupyter/colab notebook that executes an experiment from the paper. As of now, the code that was provided doesn't appear to have a pretrained model nor a simple already-set-up script that can be executed.

Open Access This Peer Review File is licensed under a Creative Commons Attribution 4.0 International License, which permits use, sharing, adaptation, distribution and reproduction in any medium or format, as long as you give appropriate credit to the original author(s) and the source, provide a link to the Creative Commons license, and indicate if changes were made.
In cases where reviewers are anonymous, credit should be given to 'Anonymous Referee' and the source.
The images or other third party material in this Peer Review File are included in the article’s Creative Commons license, unless indicated otherwise in a credit line to the material. If material is not included in the article’s Creative Commons license and your intended use is not permitted by statutory regulation or exceeds the permitted use, you will need to obtain permission directly from the copyright holder.
To view a copy of this license, visit https://creativecommons.org/licenses/by/4.0/

Reviewer #1 (Remarks to the Authors):

The authors propose a deep learning approach for aberration detection.
My recommendation would be to make the abstract more concise and to better reflect what is actually achieved in the work. Currently, the abstract rises rather too high expectations which are not really met in the work.

The main limitations of the approach as demonstrated are that only very easy aberrations are corrected which might equally well be corrected with a simple intensity based approach, maybe also using deep learning.

There are many approaches with phase elements in the detection path, and these should be cited more carefully. Overall, the approach seems rather more complicate than existing ones with better performance.

Line 202: please remove „transformative“

Response of authors

We would like to thank the reviewer for kindly dedicating the time and effort to reviewing our paper. In what follows, we address the comments of the reviewer and update the manuscript and supplementary information accordingly. The new updates of the manuscript are marked in blue.

Comments

"My recommendation would be to make the abstract more concise and to better reflect what is actually achieved in the work. Currently, the abstract rises rather too high expectations which are not really met in the work.“

Response

We thank the reviewer for this helpful suggestion. We fully agree that the abstract should concisely and accurately reflect the actual scope of the work, and we appreciate the reviewer's point that the previous version could raise expectations beyond what is demonstrated. Based on the reviewer's comment, we now updated the abstract.

“Optical aberrations fundamentally limit the performance of optical systems across scales, from microscopy and astronomy to quantum imaging and ubiquitous technologies such as smartphone cameras, autonomous vehicles, and optical communication networks. Despite sustained progress in wavefront sensing and correction, existing approaches typically require multiple intensity measurements and iterative phase retrieval algorithms, and are often restricted to narrowband operation and simple beam profiles. Their performance further degrades when aberrations are weak or obscured by noise.
Here we report a single-shot wavefront sensing and correction paradigm that overcomes the limitations of existing approaches. Central to the approach is the integration of a learned optical phase mask that acts as a physical encoder and is jointly optimized with a neural-network decoder. This encoding removes intrinsic phase ambiguities and significantly enhances sensitivity to weak aberrations. By leveraging this hybrid deep-learning architecture, our method directly and unambiguously retrieves Zernike-based phase distortions from a single focal-plane intensity image, eliminating the need for defocus measurements, or iterative phase reconstruction. The framework is intrinsically broadband, exhibits strong robustness to noise, and generalizes across a diverse class of structured light fields. Together, these capabilities establish a scalable and practical foundation for real-time aberration correction in next-generation optical and photonic systems.”

Additionally, we have revised the title, abstract, introduction, and conclusion to more precisely position the contribution as a generalizable single-shot wavefront sensing framework that enables aberration correction. All statements implying broad correction capability have been adjusted to reflect the actual demonstrated scope. The updated title is now “**An end-to-end hybrid deep-learning approach for single-shot wavefront sensing and correction**”.

We thank the reviewer again for this constructive comment, which has strengthened the manuscript.

Comments

“The main limitations of the approach as demonstrated are that only very easy aberrations are corrected which might equally well be corrected with a simple intensity based approach, maybe also using deep learning.”

Response

We thank the reviewer for this comment. To increase aberration complexity and improve the effectiveness of our proposed approach in detecting and correcting higher-order aberrations, we

trained and evaluated our network to handle aberrations represented by up to **27 Zernike polynomials**, corresponding to aberrations of up to 6th radial order. We achieved the average RMSE of $7.4 \times 10^{-2}\,\pi$ radians. Figure 1.a illustrates the distribution of errors obtained on the held-out set, while Figure 1.b presents the average L1 error for each Zernike polynomial on the same set, highlighting a consistent behavior across the Zernike modes**.** Figure 2 depicts several examples of the detection and correction process. Additionally, Figure 3 shows instances with predicted Zernike coefficients as well.

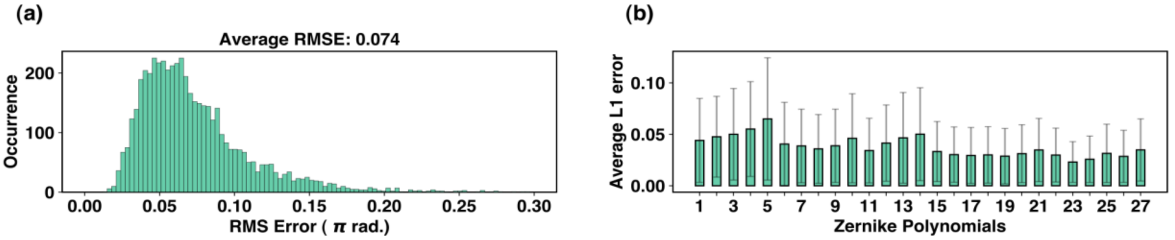

Figure 1. Evaluation results on the Z_{27} dataset. (a) error distribution on the test set for the dataset with aberrations created using the first 27 Zernike polynomials, (b) average L1 error for each Zernike polynomial on the test set.

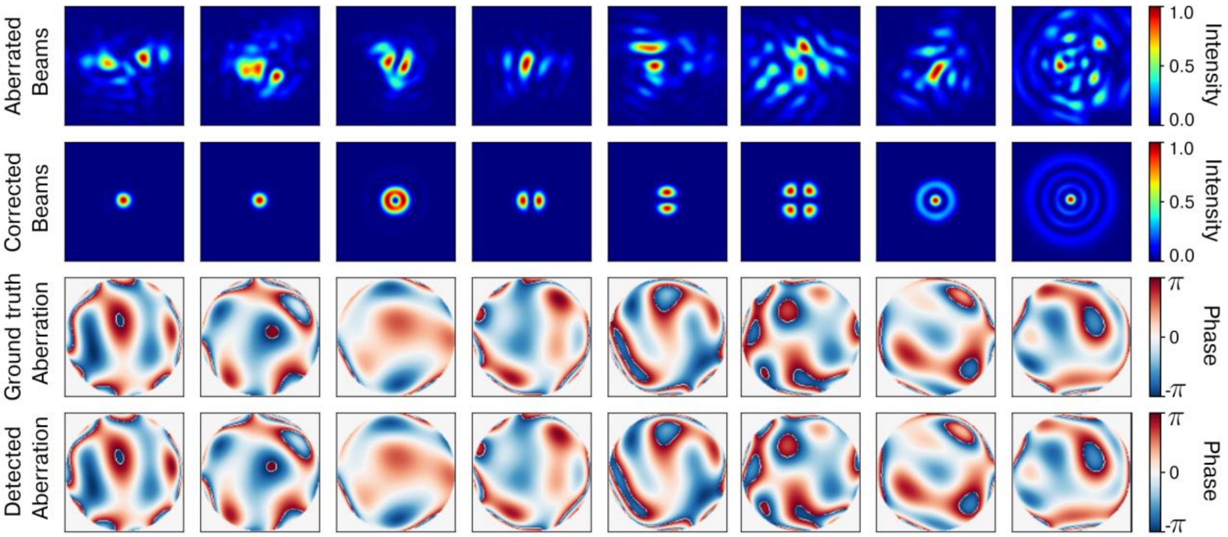

Figure 2. Qualitative examples of aberration detection and correction using our framework on the dataset with aberrations created using the first 27 Zernike polynomials.

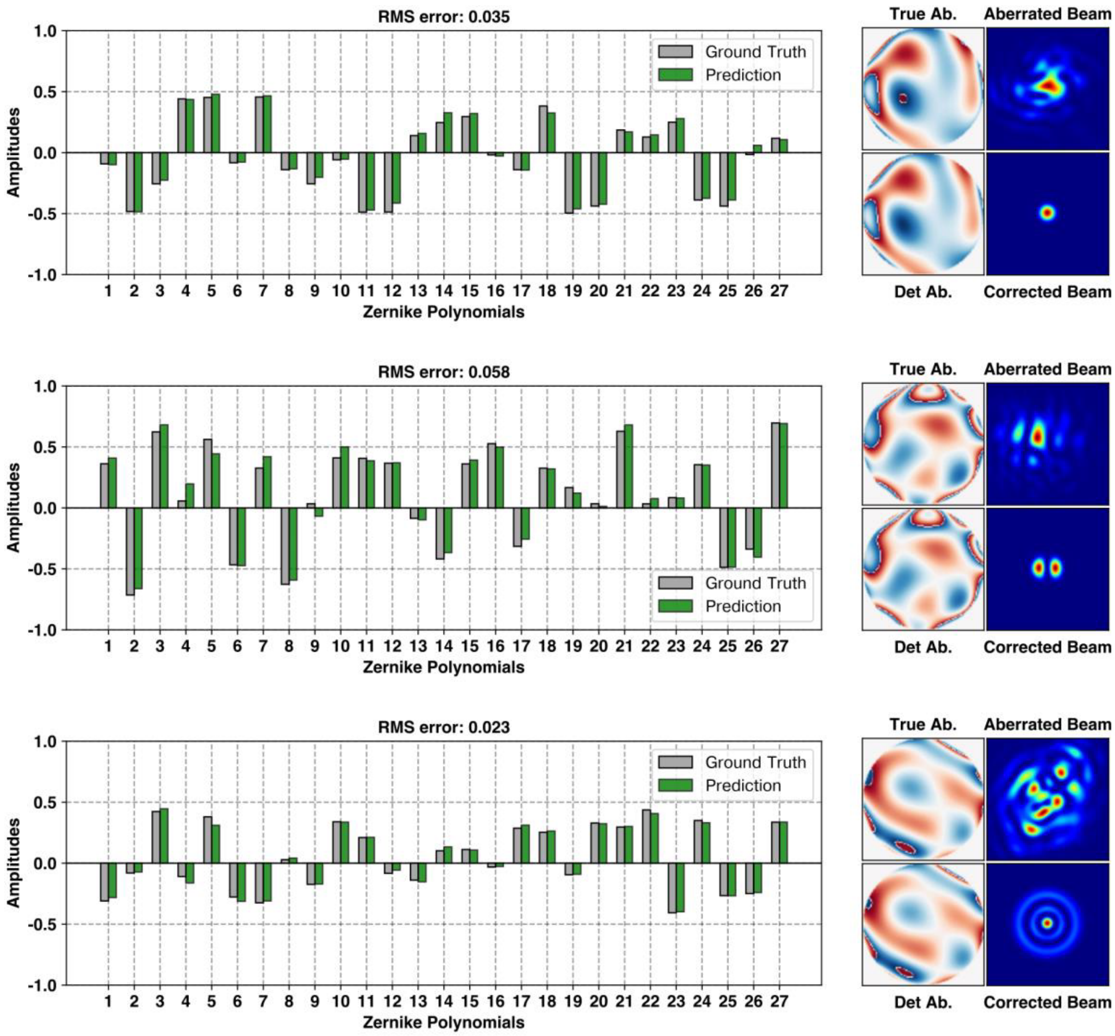

Figure 3. Qualitative examples of aberration detection and correction using our framework on the dataset with aberrations created using the first 27 Zernike polynomials in which the prediction of Zernike coefficients are also shown.

This experiment demonstrates that our method can be effectively applied to more complex scenarios. It successfully removes ambiguity and detects aberrations in higher-order beams. The framework requires only a one-time training session on the aberration domain at the beginning. After this initial training, it can perform inference significantly fast (~5ms) compared to the traditional gradient-based methods (~570ms), which is two orders of magnitude faster. We have added a section titled **"Higher-order Training and Evaluation"** to the Supplementary Information (Section 6.7) describing this experiment.

Comments

There are many approaches with phase elements in the detection path, and these should be cited more carefully.

Response

We thank the reviewer for this comment and apologize for any oversight in the previous version of the manuscript. We have revised the manuscript to more carefully and comprehensively cite prior works, including studies that use phase elements in the detection path. Below we list the additional papers that have been added to the manuscript: We will be happy to add any additional paper that the reviewer deems necessary to cite.

- *30. Li, J. et al. All-optical complex field imaging using diffractive processors. Light Sci. Appl. 13, 120 (2024).*
- *31. Shen, C.-Y. et al. All-optical phase conjugation using diffractive wavefront processing. Nat. Commun. 15, 4989 (2024).*
- *32. Fröch, J. E. et al. Computational imaging with meta-optics. Optica 12, 774 (2025).*
- *33. Arguello, H. et al. Shift-variant color-coded diffractive spectral imaging system. Optica 8, 1424 (2021).*
- *34. Lee, K. C. et al. Design and single-shot fabrication of lensless cameras with arbitrary point spread functions. Optica 10, 72 (2023).*
- *35. Zhang, F. et al. Phase retrieval by coherent modulation imaging. Nat. Commun. 7, 13367 (2016).*
- *36. Chung, J., Martinez, G. W., Lencioni, K. C., Sadda, S. R. & Yang, C. Computational aberration compensation by coded-aperture-based correction of aberration obtained from optical Fourier coding and blur estimation. Optica 6, 647 (2019).*
- *37. Wu, T., Guillon, M., Tessier, G. & Berto, P. Multiplexed wavefront sensing with a thin diffuser. Optica 11, 297 (2024).*
- *38. Gunjala, G., Sherwin, S., Shanker, A. & Waller, L. Aberration recovery by imaging a weak diffuser. Opt. Express 26, 21054 (2018).*
- *39. Berto, P., Rigneault, H. & Guillon, M. Wavefront sensing with a thin diffuser. Opt. Lett. 42, 5117 (2017).*

We also added a new paragraph describing the recently added references:

"Recently, wavefront-sensing methods rely on nontraditional optical encoders, such as photonic lanterns, optical neural networks, fixed phase masks, or diffusers, often combined with

computational reconstruction or data-driven inference. Photonic lanterns[10] have been proposed as wavefront sensors by placing them before the focal plane and reconstructing the wavefront from measured single-mode output intensities using deep learning. Emerging diffractive neural networks[11,29–31] have introduced promising hardware-encoded solutions for complex-field imaging. These include methods that map phase objects to intensity images[30] and an approach using diffractive neural networks to detect Zernike-based aberrations[11]. Photonic lanterns and diffractive neural networks, however, are constrained by computational limitations, narrowband operation, fabrication complexity, and limited compatibility with broadband, non-paraxial, or structured fields. Closely related to diffractive neural networks are works on differentiable optics[32,33] and PSF engineering[34], which aim to improve incoherent imaging. In these works, a fixed or optimized phase mask is introduced into the optical path and followed by computational reconstruction to enable lensless imaging and improve image quality. Coherent modulation imaging[35] employs a phase mask in the optical path to enhance both coherent and incoherent imaging. It often utilizes fixed, non-optimized patterns with iterative reconstruction, which may involve multiple masks[36]. Diffuser-based approaches have enabled single-shot estimation of multiple isoplanatic wavefronts[37], recovery of spatially varying aberrations via tilted illumination, and compact lenslet-free sensing that requires strict calibration and fixed geometry[38,39]. These works highlight the promise of compact, low-cost encoders; however, they remain limited in FPA detection and lack encoder phase optimization, and computational efficiency, and are less robust to noise due to beam scattering."

Comments

Overall, the approach seems rather more complicate than existing ones with better performance.

Response

We thank the reviewer for this remark. We apologize if the previous version of the manuscript was not clearly explaining the method. We have now revised the method section and the Supplementary Information to present the proposed method more clearly.

To highlight the performance of our proposed method compared to simple intensity-based approaches in a single-shot setting, we conducted an extensive comparison with the Gerchberg-Saxton (GS) algorithm and a Gradient-Descent-Based (GDB) optimization method, evaluating their performance on experimentally captured aberrated beams. Furthermore, to demonstrate the effectiveness of our optimal phase plate (i.e., the proposed trained bias), we compared the performance of our approach, GS, and GDB both with and without the use of the bias. In this

comparison, “APN without the bias” corresponds to a deep learning approach without any phase elements in the optical path. We conducted experiments using both the uniform and Kolmogorov datasets that were captured. The following table displays the average RMSE obtained for each dataset:

Distribution / Model	Uniform Distribution (RMSE π rad.)				Kolmogorov Distribution (RMSE π rad.)			
	Gaussian	OAM (l=1)	HG_{10}	Total	Gaussian	OAM (l=1)	HG_{10}	Total
**GS w/o Bias**	0.452 ± .120	0.403 ± 0.124	0.455 ± 0.122	**0.437±0.124**	0.295 ± 0.099	0.238 ± 0.083	0.323 ± 0.130	**0.285±0.111**
**GS w Bias**	0.460 ± 0.135	0.416 ± 0.127	0.462 ± 0.121	**0.446±0.130**	0.302 ± 0.114	0.243 ± 0.083	0.312 ± 0.123	**0.285±0.112**
**GDB w/o Bias**	0.333 ± 0.227	0.298 ± 0.235	0.365 ± 0.208	**0.332±0.225**	0.234 ± 0.198	0.118 ± 0.144	0.260 ± 0.182	**0.204±0.186**
**GDB w Bias**	0.167 ± 0.181	0.195 ± 0.229	0.251 ± 0.204	**0.204±0.209**	0.082 ± 0.149	0.081 ± 0.084	0.178 ± 0.139	**0.114±0.135**
**APN w/o Bias**	0.293 ± 0.127	0.125 ± 0.045	0.327 ± 0.125	**0.248±0.138**	0.224 ± 0.106	0.110 ± 0.042	0.277 ± 0.124	**0.204±0.120**
**(Our work) APN w Bias**	**0.074 ± 0.027**	**0.067 ± 0.023**	**0.110 ± 0.049**	**0.083±0.038**	**0.059 ± 0.017**	**0.057 ± 0.016**	**0.090± 0.033**	**0.069±0.028**

Table 1. Comparison of our approach to other single-shot intensity-based approaches using experimentally captured aberrated beam images.

The results indicate that the traditional single-shot intensity-based method, which lacks any phase elements in the optical path, has a large margin of error due to focal point ambiguity (RMSE of $43.7 \times 10^{-2}\ \pi$ radians for GS and RMSE of $33.2 \times 10^{-2}\ \pi$ radians for GDB for uniform dataset). In contrast, our approach achieved the lowest RMSE across both datasets, outperforming all other methods (RMSE of $8.3 \times 10^{-2}\ \pi$ radians for uniform dataset). It is important to note that by utilizing the bias, we have improved not only the neural network-based estimator (66.5% reduction in RMSE) but also the gradient-based estimator (38.5% reduction in RMSE). This demonstrates the effectiveness of the trained bias in enhancing the function's injectivity.

The GBD approach took an average of 570 ms to converge and predict a single aberration, while our approach required less than 5 ms for the same estimation (two orders of magnitude faster), as it does not rely on iterative reconstruction. In contrast, the Gerchberg-Saxton algorithm, implemented on the CPU, took significantly longer to converge due to its dependence on large Fourier transforms.

We have updated the Discussion section and Supplementary Section 7.3.3 to include these results.

Comments

Line 202: please remove „transformative“

Response

We thank the reviewer for this comment. The “Transformative” term is now removed in the revised manuscript.

Concluding remarks of authors

We would like to thank the reviewer again for the exceptional quality of their questions. We hope our responses have fully addressed all expressed concerns. The referee comments have helped us significantly to improve the quality of the paper and its presentation. We are happy to address any further questions and implement any modifications the reviewers suggest.

Reviewer #2 (Remarks to the Authors):

This paper presents a new method for optical wavefront sensing which jointly learns a diffractive phase mask to encode the wavefront into intensity and a reconstruction deep learning model which maps the intensity back to an estimate of the wavefront. The key insight of the paper is that one of the ambiguities of the standard phase to intensity mapping via far field diffraction is that phases with negative or positive even components map to the same intensity. Thus by inserting a mask of sufficient complexity, this ambiguity is no longer observable within the realm of reasonably observable phases. By learning this phase mask jointly with a intensity to phase mapping, the authors are able to simultaneously improve the injectivity of the phase to intensity mapping and the ability to invert it. They term this strategy Aberration Prediction Network (APN) and demonstrate its viability in simulation and experimentally.

To train APN they created simulated pairs of phase maps (parametrized with low order Zernike coefficients < 4th order) and intensity distributions (via bluestein diffraction method), and trained the model to predict the coefficients given the intensity. They then tested the model on held out simulation data of the same type (zernike < 4th order) both uniformly sampled and correlated according to Kolmogorov turbulence covariances. Experimentally, they ran similar experiments by expressing the true aberrations on an SLM, recording with either an SLM displaying the learned mask or a custom made metasurface. They are able to then apply the conjugate estimate phase to retrieve the unaberrated wavefront. They run these experiments over different beam types, a 200nm range of visible wavelengths, and up to 1pi radian of RMS aberration strength

Overall the performance of their method is impressive. Even in experiments, they show relatively high accuracy in estimating low order aberrations to the point where correcting them works well. Their methodology is also nicely motivated as they show the existence of one possible phase to intensity ambiguity and describe how to fix it. Their use of learning seems rigorous and effective, accounting for hyperparameters and model mismatch (via augmentation). They also do a great job of validating/ablating various components of their method including showing the state of ambiguity before and after the bias as well as comparing to fixed biases. I think the overall approach is novel both in the sense that it effectively uses end-to-end learning for wavefront sensing and because it recognizes (and demonstrably fixes) the even phase component ambiguity.

While, I think this approach could have great value to the community I do think there are a few important points to address, additional suggested experiments, and other clarifying questions/suggestions, which I put below. I apologize in advance for any mistakes or misreads

I may have and am looking forward to a productive exchange with the intent of improving this paper as much as possible!

Response of authors

We would like to thank the reviewer for kindly dedicating the time and effort to reviewing our paper. We greatly appreciate the highly constructive comments from the reviewer *"Overall the performance of their method is impressive. Even in experiments, they show relatively high accuracy in estimating low order aberrations to the point where correcting them works well. Their methodology is also nicely motivated as they show the existence of one possible phase to intensity ambiguity and describe how to fix it. Their use of learning seems rigorous and effective, accounting for hyperparameters and model mismatch (via augmentation). They also do a great job of validating/ablating various components of their method including showing the state of ambiguity before and after the bias as well as comparing to fixed biases. I think the overall approach is novel both in the sense that it effectively uses end-to-end learning for wavefront sensing and because it recognizes (and demonstrably fixes) the even phase component ambiguity. I think this approach could have great value to the community"*.

In what follows, we address the comments of the reviewer and update the manuscript and supplementary information accordingly. The new updates of the manuscript are marked in blue.

Comments

Before getting to specifics, I want to summarize that the core limitation of this paper is that the authors never display this system in an actual application setup (e.g., imaging, metrology, etc). Thus claims of its use as a universal aberration corrector are a bit of a reach without further validation. Instead what the authors have shown is a novel method for wavefront sensing, which, if further applied could be used in the context of aberration correction. Suggestions to that end are put below including a few main points: *language of the paper* (including the title) should reflect this limited scope, the method appears to not be evaluated in a *realistic SNR regime* (its unclear what the laser power is) and is never directly evaluated on *aberrations outside of those* generated by the first 14 Zernike coefficients which the model is trained on.

Response

We thank the reviewer for these detailed comments and fully share the reviewer's goal of strengthening the quality and clarity of the manuscript. Based on her/his comments and recommendations, we address the comments of the reviewer and update the manuscript and supplementary information accordingly.

Comments

Why are showing multiple beam types outside of Gaussian useful? Where do they come up in the applications you mention? Maybe provide an example or two since readers (including myself) may not be familiar with why they are important.

Response

We thank the reviewer for this question. We consider multiple beam types beyond the Gaussian to reflect real-world imaging and beam-shaping applications, where structured beams are commonly used and their performance depends critically on the preservation of spatial phase. Structured beams have emerged as a cornerstone of modern photonics, owing to their unique phase singularity and orbital angular momentum, which enable functionalities unattainable with conventional Gaussian beams. They play a central role in a broad range of high-impact applications, including optical trapping and micromanipulation, super-resolution imaging, high-capacity optical communications, and quantum information science[1–5]. A key advantage of vortex beams is their flexible generation, detection, and control across the visible and infrared spectrum, making them practical for real experimental and technological implementations.

A particularly compelling example is stimulated emission depletion (STED) microscopy using Laguerre-Gaussian (LG), where the helical phase structure $e^{il\phi}$of a first-order optical vortex creates a robust central intensity null that is essential for breaking the Abbe diffraction limit. This vortex-induced dark core is not merely beneficial but fundamentally required to achieve nanoscale resolution. Using visible wavelengths, STED microscopy has demonstrated resolutions on the order of $\lambda/100$, showing the transformative impact of vortex beams on optical imaging[6].

In quantum domains, it was recently demonstrated that using OAM to encode Bell states not merely realizes a high-dimensional Hilbert space, but also enhances the ability of quantum states to carry information[7–12]. In astronomy, where one may wish to block light from a bright star so as to make orbiting planets more visible. Such applications using spiral phase plates are currently under investigation and trial[13].

Additionally, OAM beams can serve as modulation matrices for single-pixel imaging by simultaneously undergoing angular and radial modulation, thereby enabling OAM-based single-pixel imaging. Zhao and colleagues successfully implemented OAM complex spectral analysis and single-pixel complex amplitude imaging using Laguerre-Gaussian (LG) beams as the modulation matrix[14].

Following the reviewer's suggestion, we have updated the Introduction to better highlight the significance of structured light fields.

Comments

I feel that the writing throughout borders on grandiose and, at times, overclaims. I suggest going through the writing and shifting the focus away from how it's a fundamental methodological breakthrough or how it will perform amazingly in imaging applications to instead in what ways your technique is quantifiably better than existing wavefront sensing, and how that perhaps could improve certain applications that use wavefront sensing/correction. At its core this work uses a typical direct wavefront sensing setup but jointly learns the encoding element and decoding algorithm. It then shows that such an approach works in a proof-of-concept setup.

Response

We thank the reviewer for this remark and apologize if any part of the manuscript appeared to be overstated. We have carefully revised the manuscript to reflect the scope and contributions of our work more accurately. Specifically, we have focused on how our approach differs from the existing methods (Introduction and Discussion), how it compares to other intensity-based approaches (Supplementary Information Section 7.3.3), how it can be applied in optical systems (Discussion section and Supplementary Information Section 9), and the improvements it offers. The manuscript has been updated to address the reviewer's suggestions, and the revised text is highlighted in blue in both the main manuscript and the Supplementary Information.

We thank the reviewer again for this constructive comment, which has strengthened the manuscript.

Comments

The title for example 'One image, all aberrations' and 'universal framework for aberration correction' feels like a pretty strong overclaim. Indeed your method only demonstrates correction over the partial visible range, lower order zernike aberrations which are monochromatic (i.e., not chromatic aberrations) and spatially-invariant. Moreover, correction is does not seem like the main contribution of the paper, but rather wavefront sensing (no new methodology for correction is presented). Finally, you don't ever demonstrate it in an actual imaging context. I would tone down this title and make it more scientifically informative (e.g., "A end-to-end hybrid deep-learning approach for single-shot wavefront sensing") or something

of the sort where the focus is on your contribution (single-shot wavefront sensing) and methodology (end-to-end learning of a phase bias and prediction network).

Response

We thank the reviewer for this thoughtful and constructive feedback regarding the title. We acknowledge that the previous phrasing may have suggested a broader scope than what is explicitly demonstrated. Following reviewer comment, we now changed the title to "***An end-to-end hybrid deep-learning approach for single-shot wavefront sensing and correction."*** The title is now updated in the main manuscript and the supplementary information.

Comments

Another example is something like "The proposed framework will enable cutting-edge imaging modalities, including high-resolution visualization of tissues through multilayered cellular structures with varying refractive indices, thereby delivering unparalleled spatial resolution and facilitating precise quantitative analysis within complex biological environments crucial for biomedical diagnostics and research." is a pretty large overclaim given that you never did any imaging experiments. It's fine to suggest future applications of your method and why it might the improve status quo but to suggest that it will create 'unparalleled resolution' is a large leap in logic.

Response

We thank the reviewer for this important clarification and fully agree with her/his assessment. The original phrasing in the conclusion overstated the immediate implications of our work, particularly with respect to biological imaging performance and spatial resolution, which were not experimentally demonstrated in this study. In response, we have revised the conclusion of the paper to remove language suggesting these (see below in blue):

"In conclusion, we present a framework for aberration sensing and correction that operates using single focal-plane intensity image. The method demonstrates accurate recovery of system aberrations in Zernike space across a wide range of illumination powers, numerical apertures, and optical configurations, without requiring additional training data or specialized calibration beyond standard SLM characterization. By incorporating the aberration-correction module into the imaging path and sampling a limited fraction of the focal-plane intensity image, wavefront

distortions can be inferred and pre-compensated with low latency, enabling improved aberration control at the specimen. This capability points toward future developments in applications that benefit from precise wavefront control, including structured-beam microscopy, high-resolution imaging, and controlled beam-matter interactions."

Comments

"Here, we report a unified, single-shot wavefront correction framework that departs fundamentally from conventional approaches." or "In summary, we present a transformative single-shot wavefront correction framework that fundamentally redefines conventional paradigms.". The claim of departing fundamentally for conventional approaches is unsubstantiated; it is the conventional setup of many wavefront sensing techniques to encode the wavefront via some optic (diffractive, lenslet, etc) and then map the resulting intensity back to phase via computation. The proposed approach builds on this line of thinking by jointly learning mask and reconstruction algorithm.

Response

We thank the reviewer for this thoughtful comment and agree that the original wording overstated the conceptual departure of our approach from prior wavefront sensing methodologies. Following reviewer comment, this sentence is now changed to:

"Here, we demonstrate a single-shot approach to wavefront sensing and correction that overcomes the aforementioned limitations of conventional techniques. Using a hybrid deep-learning architecture, the method directly reconstructs phase distortions in Zernike space from a single focal-plane intensity image, without requiring defocus measurements, iterative phase retrieval, or prior assumptions about the input beam profile. Beyond its conceptual simplicity, the approach is intrinsically broadband and demonstrates strong robustness to detector noise, low-signal conditions, and saturation effects. Importantly, it enhances the sensitivity of intensity-only measurements to weak aberrations that are otherwise difficult to detect. The framework further generalizes across a wide range of structured optical fields, including Orbital-Angular-Momentum (OAM), Hermite-Gaussian, and Laguerre-Gaussian modes."

Comments

"Our framework establishes a scalable foundation for real-time aberration correction in advanced optical systems, while simultaneously offering fundamental insights into the origin and behavior of optical aberrations". I don't think there is any evidence of this paper to support new insights into the "origin and behavior of optical aberrations". Perhaps you can claim

insight into the nature of wavefront sensing ambiguity (e.g., via the FPA argument) but not aberrations themselves.

Response

We thank the reviewer for this thoughtful comment and agree that the original wording overstated "origin and behavior of optical aberrations". The conclusion/discussion sections have been revised to reflect these changes (see below in blue):

"In conclusion, we present a framework for aberration sensing and correction that operates using single focal-plane intensity image. The method demonstrates accurate recovery of system aberrations in Zernike space across a wide range of illumination powers, numerical apertures, and optical configurations, without requiring additional training data or specialized calibration beyond standard SLM characterization. By incorporating the aberration-correction module into the imaging path and sampling a limited fraction of the focal-plane intensity image, wavefront distortions can be inferred and pre-compensated with low latency, enabling improved aberration control at the specimen. This capability points toward future developments in applications that benefit from precise wavefront control, including structured-beam microscopy, high-resolution imaging, and controlled beam-matter interactions."

Comments

What about dynamic aberrations and/or shift-varying aberrations; these are common in biological tissue and atmospheric turbulence. It is out of scope but can you at least put a discussion about how this method could extend to compensate for it? Perhaps it is as simple as just calibrating a per field-of-view point coefficient set and then sequentially applying correction for a point scanning system? But what if it's not a point scanning system?

-For example: https://opg.optica.org/optica/fulltext.cfm?uri=optica-11-2-297

Response

We thank the reviewer for highlighting this important point regarding dynamic and shift varying aberrations, which are common in biological tissue and atmospheric turbulence.

Regarding dynamic aberrations, since our framework does not rely on iterative correction or optimization loops, it enables fast aberration prediction at inference time. A single aberration estimation using our APN takes under 5ms, corresponding to a refresh rate of 200 frames per

second. In contrast, the gradient-based method that we evaluated requires, on average, 570ms to converge, resulting in a refresh rate of less than 2 frames per second. This capability makes our approach well suited for detecting time-varying aberrations. The SLM refresh rate ultimately determines the achievable correction speed and the temporal dynamics of the system. In our current implementation, the SLM operates at a refresh rate of 60 Hz so our system can temporally resolve aberrations under this refresh rate.

For the shift varying aberrations, we agree with the reviewer that it is as simple as just calibrating a per field-of-view point coefficient set and then sequentially applying correction for a point scanning system. Our current model handles on-axis configurations, with Zernike coefficients independent of source position. Extending to off-axis or shift-varying aberrations would require estimating separate coefficient sets for different field points, which can be efficiently done due to our fast prediction at inference time. In systems that are not point-scanning, the image must first be translated into the appropriate reference frame corresponding to the local aberration profile. In this way, the observed image is effectively treated as if the aberrations are spatially invariant within that localized region, enabling correct compensation. Based on the reviewer suggestion we have added this topic to the discussion section.

Comments

I feel that the related work section could talk about similar methods that also use a nontraditional element (like a diffuser) as an encoder and then do computational reconstruction (e.g., https://opg.optica.org/ol/abstract.cfm?uri=ol-42-24-5117). They often share properties of being inexpensive, compact, broadband, etc.

Response

We thank the reviewer for this helpful suggestion and for pointing us to diffuser-based approaches and approaches that use nontraditional elements for wavefront sensing. We have revised the manuscript to include a more comprehensive review of related works, including the references mentioned by the reviewer. Please find the revised paragraph below:

"Recently, wavefront-sensing methods rely on nontraditional optical encoders, such as photonic lanterns, optical neural networks, fixed phase masks, or diffusers, often combined with computational reconstruction or data-driven inference. Photonic lanterns[10] have been proposed as wavefront sensors by placing them before the focal plane and reconstructing the wavefront from measured single-mode output intensities using deep learning. Emerging diffractive neural networks[11,29–31] have introduced promising hardware-encoded solutions for complex-field imaging. These include methods that map phase objects to intensity images[30] and an approach

using diffractive neural networks to detect Zernike-based aberrations[11]. Photonic lanterns and diffractive neural networks, however, are constrained by computational limitations, narrowband operation, fabrication complexity, and limited compatibility with broadband, non-paraxial, or structured fields. Closely related to diffractive neural networks are works on differentiable optics[32,33] and PSF engineering[34], which aim to improve incoherent imaging. In these works, a fixed or optimized phase mask is introduced into the optical path and followed by computational reconstruction to enable lensless imaging and improve image quality. Coherent modulation imaging[35] employs a phase mask in the optical path to enhance both coherent and incoherent imaging. It often utilizes fixed, non-optimized patterns with iterative reconstruction, which may involve multiple masks[36]. Diffuser-based approaches have enabled single-shot estimation of multiple isoplanatic wavefronts[37], recovery of spatially varying aberrations via tilted illumination, and compact lenslet-free sensing that requires strict calibration and fixed geometry[38,39]. These works highlight the promise of compact, low-cost encoders; however, they remain limited in FPA detection and lack encoder phase optimization, and computational efficiency, and are less robust to noise due to beam scattering."

Comments

Why not compare to a shack-hartmann wavefront sensor?

I don't think it's necessary to actually do experimentally, but at least explain more specifically in what ways your method is better (speed, accuracy, etc). You mention a bit in the related work but it'd be nice for a practitioner to understand what benefits they'd get by switching to your system by comparing your results with the expected results of a traditional wavefront sensing system like shack-hartmann, which for many is the status quo.

Response

We thank the reviewer for this constructive suggestion. To provide a concrete comparison, we implemented a Shack-Hartmann wavefront sensor (SHWFS) numerically and compared its performance with our method. We used a 20x20 lenslet array mask, which is adequate for the spatial frequency of our dataset. Additionally, increasing the number of lenslets did not enhance accuracy, and only increased the computational burden. For each lenslet, we computed the intensity response, determined the local slope from the centroid displacement, and reconstructed Zernike coefficients using a least-squares method[15]. An example of this process is illustrated in the following figure:

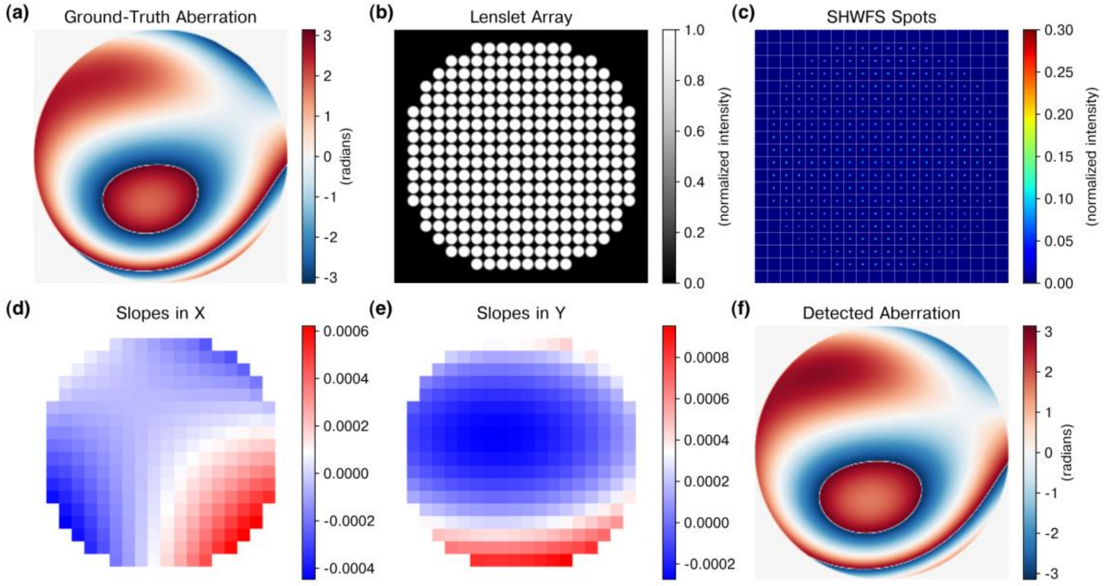

Figure 1. Shack Hartmann simulation process.

To evaluate the robustness of Shack-Hartmann in low-light conditions, we modeled shot noise and read noise with the same configuration used during our new evaluations in Supplementary Information Section 6.5. We set the brightest pixel of each intensity image to receive an average of 500 photons, considering a read noise with a standard deviation of 2. This mild noise disrupted the centroid calculations, as illustrated in the accompanying figure. Consequently, the slopes are no longer accurate, leading to a deterioration in overall accuracy.

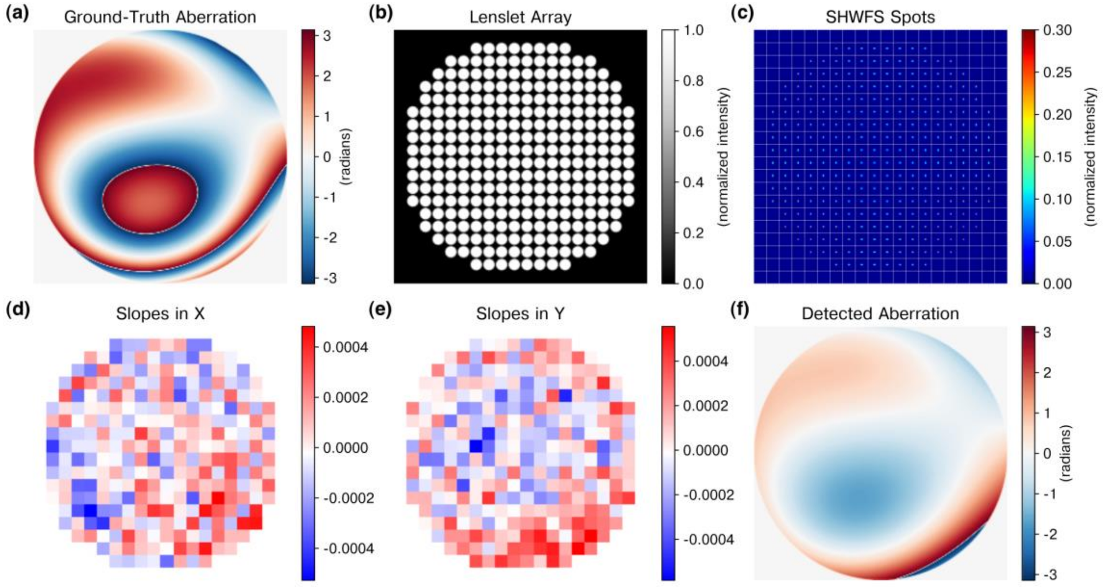

Figure 2. Shack Hartmann simulation process in presence of noise.

We further evaluated SHWFS on the Kolmogorov dataset (Z_9, D/r_o=5) and compared it to our method, both with and without noise. The evaluation was limited to only the Gaussian beam, as structured beams lead to significantly larger errors in the presence of noise for SHWFS. In the case without noise, the RMS error obtained from our method is 2.3×10^{-2} π radians, while the error associated with the Shack-Hartmann Wavefront Sensor (SHWFS) is 2.8×10^{-2} π radians which is **still higher even under ideal conditions**. In contrast, when mild photon shot noise is introduced into the system, the error for SHWFS increases significantly to 27.8×10^{-2} π radians. Meanwhile, our method maintains a consistent error of 2.5×10^{-2} π radians. Several examples of the detection and correction process are presented here:

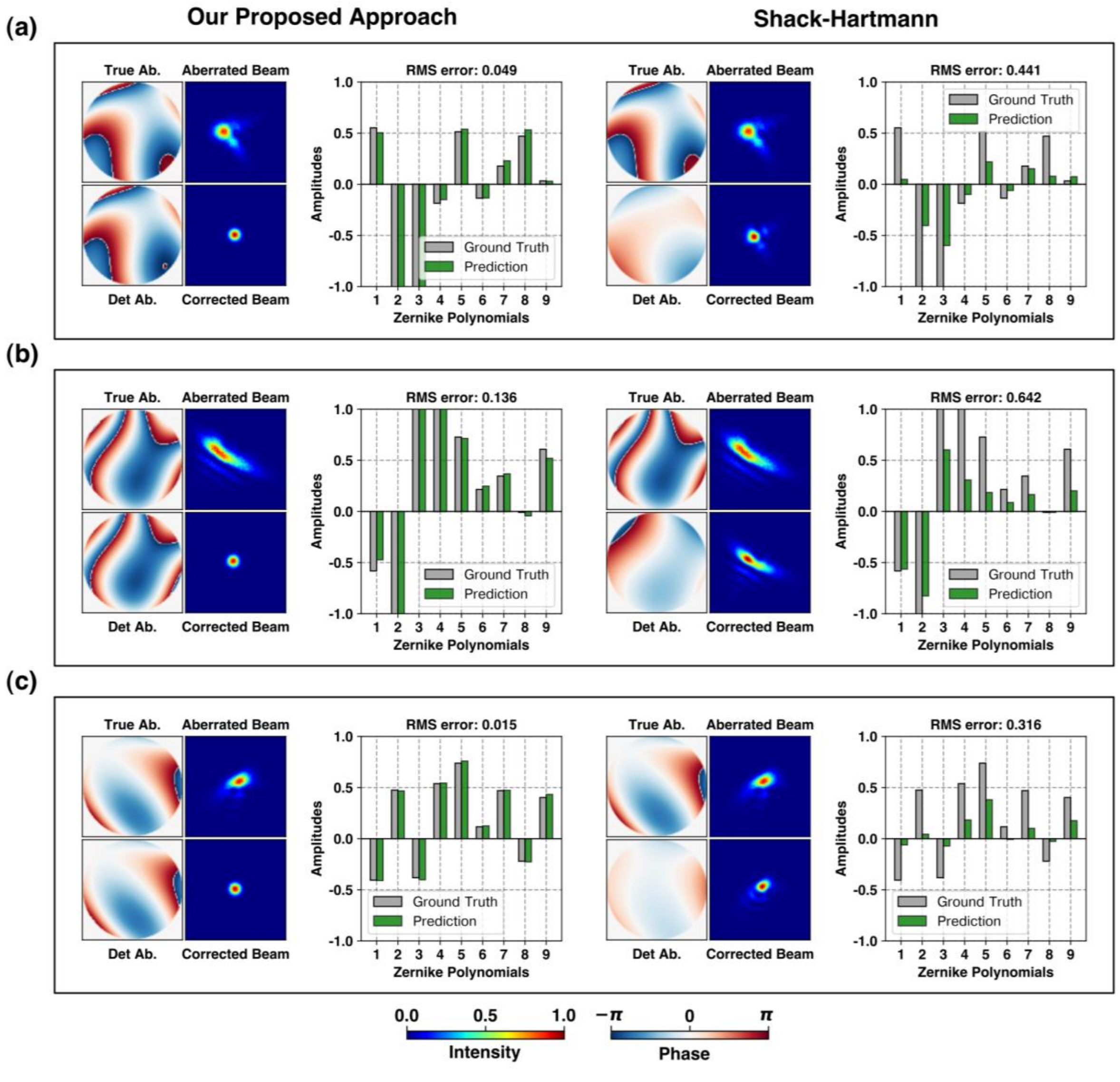

Figure 3. Instances illustrating the detection and correction process, comparing our method to the simulated Shack-Hartmann approach in the presence of noise.

The results indicate that the mild noise disrupted the performance of the SHWS. While the polarity of the Zernike modes was identified correctly, the magnitudes of each mode could not be accurately detected due to the noise. This limitation arises because SHWFS reconstruction depends on the precise estimation of spot centroids, and its performance is significantly affected by photon shot noise, camera read noise, and other fluctuations in intensity. This makes SHWFS particularly non-robust in low-light conditions, further limiting its applicability in many practical scenarios. In contrast, our network demonstrates consistent performance even in noisy conditions. The APN does not depend on the intensities of individual pixels; instead, it focuses on the local correlations between each pixel and its neighbors. As a result, the reconstruction is more robust to various types of noise.

Although Shack-Hartmann sensors offer the advantage of not requiring a reference wavefront, their performance is highly sensitive to vibrations, and the discretized sampling of the wavefront, due to pixelation of the microlens array, and consequently of the camera, inevitably leads to loss of spatial information. Most commercial Shack-Hartmann sensors provide no more than ~100um microlens array pitch, insufficient for many high-resolution applications. Even if some recent advances in microlens-array design improve sampling density but do not resolve the underlying dependence on mechanical stability, the discrete nature of measurements, or the need to infer aberration coefficients through indirect two-dimensional centroid analysis. Based on the reviewer suggestion, we have revised the introduction and discussion sections to better express the limitations of Shack-Hartmann sensors and highlight the aspects in which our approach surpasses SWFS. Additionally, we have included a section titled **"Shack-Hartmann Comparison"** in Supplementary Information Section 10, which presents these findings.

Comments

Is the network explicitly trained with different wavelengths or only a single wavelength? That is, when you generate your training data with Bluestein's method which wavelength(s) do you use? Is it possible that given your imaging geometry, wavelength simply does not have enough impact which is why you observe the broadband behavior?

Response

We thank the reviewer for bringing up this question. The Bluestein method operates with a fixed wavelength of 400 nm to generate the training data. Since we capture the intensity at the focal plane, changes in wavelength only change the geometrical scale of the intensity image ($\lambda/2NA$). As a result, capturing the intensity shape with just one wavelength is sufficient. To ensure that the model remains scale invariant in the entire bandwidth, the APN is trained with different scale sizes that mimic variations in wavelength within the bandwidth. We apologize for any lack of

clarity. We have updated the Methods Section of the manuscript and Section 5 of the Supplementary Information to provide better clarification regarding the wavelength.

Comments

What about varying noise levels? I'm worried that a good chunk of success of this method is due to have a ton of light at the wavefront sensor which is often not this case in applications like astronomy and microscopy.

Response

We thank the reviewer for raising this important concern regarding noise levels and the light intensity especially given the challenges of low-light conditions in astronomy, due to extremely distant sources, and in microscopy, due to scattering and attenuation.

To clarify that the success of our method does not rely on extremely high light levels, we note that, in our experiments, an ideal Gaussian beam at 600 nm would focus to a $1/e^2$ diameter of approximately 0.96 mm, and to about 1-2 mm when aberrations increase the k-vector spread and lower the overall intensity. This corresponded to roughly 8 μJ of energy per frame, which was sufficient to capture the beam profile without saturating the sensor and while still preserving the features needed to correct the beam. Importantly, our method does not require high-power illumination, it performs reliably even at the modest light levels. Using standardized gain and exposure times, we ensured that the sensor did not saturate under zero-aberration conditions, where intensity is highest, yet still captured sufficient contrast when aberrations redistributed energy resulting in lower intensities throughout the sensor. Notably, the system was able to reliably detect aberrations under these conditions.

Based on the reviewer's suggestion and to highlight network performance in situations where the camera does not receive a sufficient amount of light, we have added evaluations for low light conditions, defined by poor signal-to-noise ratio (SNR) (added in the Supplementary Information Section 6.5). This is also addressed in the next comment. Several examples of detection and correction processes under low light conditions (where the peak of a diffraction-limited beam only receives an average of 100 photons) are illustrated in the figure below. Please note that the intensity images are normalized before being fed to the network.

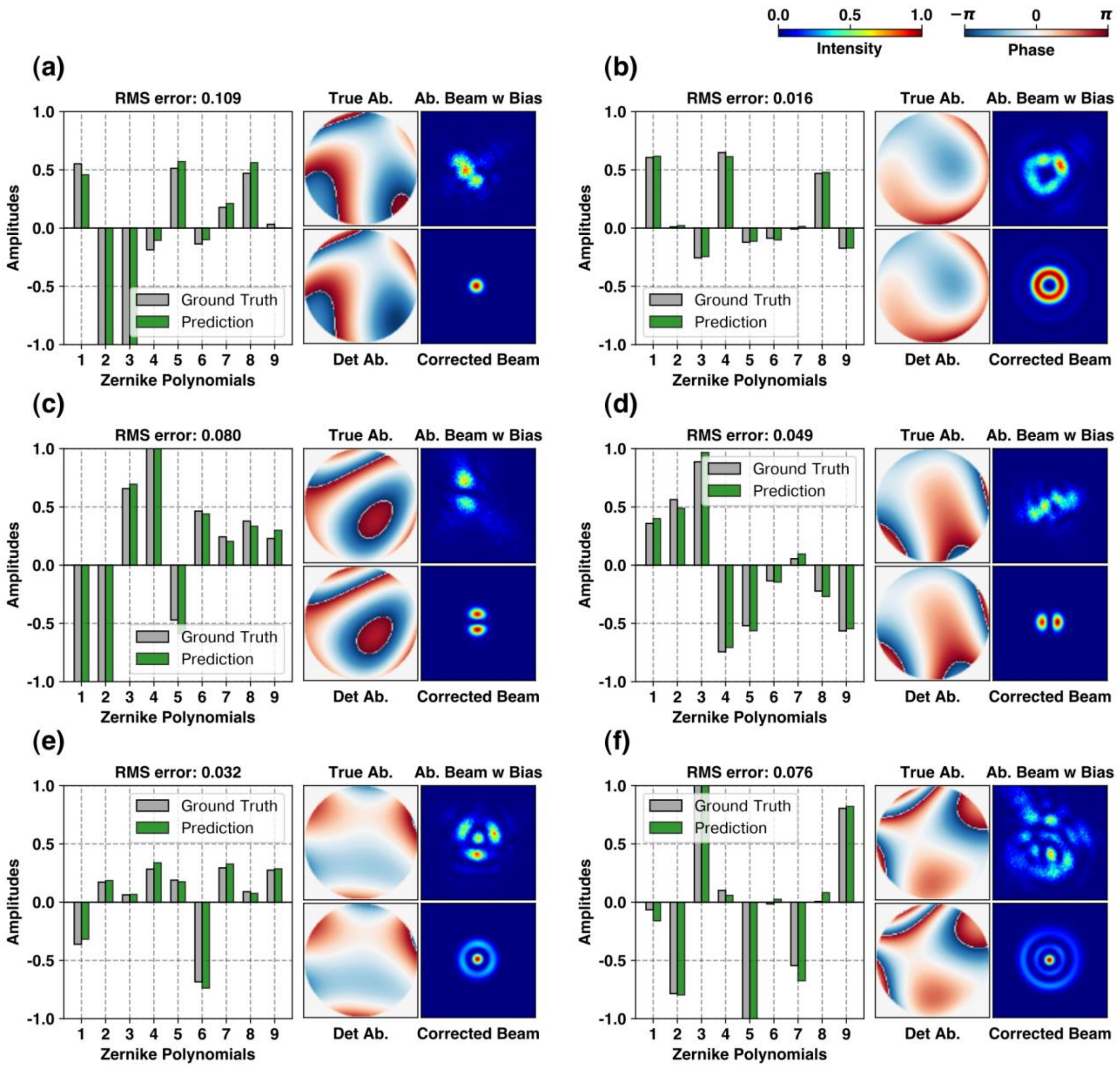

Figure 4. Qualitative examples of aberration detection using our framework in the presence of photon shot noise with 100 average photons received at the brightest pixel in the diffraction limited beam.

The results demonstrate the robust behavior of our network, even when the input intensity image contains noise. This is because our network is equipped with a regularization module (Supplementary Information Section 7.2) that prevents it from overfitting and relying on specific individual pixel values for detection.

Comments

To that end please discuss the power of the laser you used (I can't find it anywhere) and talk about the amount of light hitting your wavefront sensor and compare it to a standard level as would be seen in a adaptive optics context. If possible, I would love to see a small experiment showing the performance of the network at low signal levels (this can even be done in simulation)

Response

We thank the reviewer for this question, and we apologize for missing out on the details regarding the laser power and the associated parameters as it will make our argument about our model holistic. We also acknowledge the reviewer's suggestion regarding low-light conditions, as they are important considerations.

For practical experiments, the power of the Gaussian beam was around 50 µW. For the sensor, we used a BFS-U3-200S6C-C Blackfly USB 3.1 camera with an exposure range of 69 µs to 30 s and a gain range of 0-27 dB. All our data were collected at 0 dB gain and 80 ms exposure. For our experiments, we used a pulsed supercontinuum laser (NKT SuperK Fianium 15) together with an acousto-optic modulator to select the desired wavelengths. The tunable acousto-optic filter allowed us to isolate the specific wavelength of interest for each measurement. We then used a SuperK Connect module to couple the selected wavelength into a single-mode fiber, and an aspheric lens to collimate the output. This ensured that the beam emerging from the fiber was a high-purity Gaussian, providing a clean and well-defined input for all subsequent experiments.

Following the reviewer's suggestion, we evaluated the model's performance in low-light conditions, which are characterized by a low signal-to-noise ratio (SNR). To this end, we conducted a series of assessments and added a section titled **"Low-light Evaluations"** to the Supplementary Information (Section 6.5). In these evaluations, we modeled shot noise and camera read-out noise, and we observed the accuracy of our proposed method under these conditions. During these evaluations, we utilized the APN, which was trained with a noise-robust module. This module acts as a regularizer, helping the network to prevent overfitting and learn the dominant features in the image instead of relying on individual pixel values. We have utilized the following approach to model the SNR in the system:

$$\mathrm{SNR} = \frac{\mathrm{S}}{\sqrt{\sigma_{\mathrm{S}}^2 + \sigma_{\mathrm{D}}^2 + \sigma_{\mathrm{R}}^2}}$$

Where S is the total detected number of photons, σ_S^2 is the photon shot noise, σ_D^2 is the dark shot noise, and σ_R^2 is the read noise. We assume a quantum efficiency of one. The shot noise terms are modeled using a Poisson distribution, while the read noise is modeled using a Gaussian distribution. We evaluated our approach using two different configurations. In the first set of

evaluations, we assumed that the pixel with the highest intensity for each aberration receives on average a fixed number of photons in the presence of both dark shot noise (σ_D^2=2), and read noise (σ_S^2=2). In this scenario, the SNR for each type of aberration remains constant, and each intensity image is affected by a predefined, fixed amount of noise. Specifically, the brightest pixel is assumed to receive between 100 and 900 photons. When dark noise and read noise are taken into account, this corresponds to a linear SNR ranging from 9.71 to 29.90. We evaluated the model using the Kolmogorov dataset (Z_9, D/r_o=5), and the results are shown in the following figure:

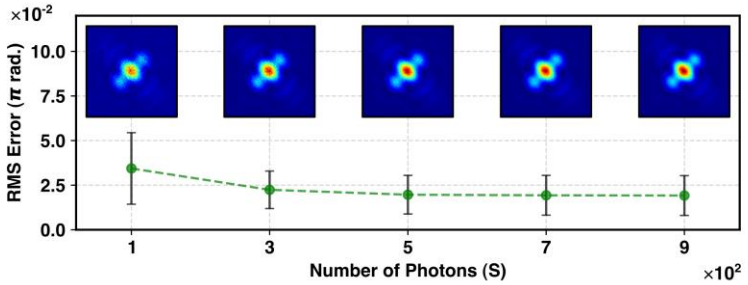

Figure 5. The performance of the model under low-light conditions. Evaluation has been done in the presence of photon shot noise, dark noise and read-out noise. The number of photons indicates the average photons received by the brightest pixel in each aberration. The insets in the figure show a Gaussian beam aberrated with a positive value of oblique astigmatism.

The results demonstrate the consistent performance of the network in the presence of both shot noise and camera noise. In the second set of evaluations, which are photon-limited, we used identical exposure settings for all aberrations. As a reference, we assigned the photon count based on the brightest pixel in the diffraction-limited beam, which serves as the baseline for all other beams. For example, in the 100-photon setting, the brightest pixel in the diffraction-limited beam receives, on average, 100 photons. For aberrated beams, the brightest pixel has a lower intensity than in the diffraction-limited case and therefore receives a proportionally smaller number of photons. This configuration models a realistic scenario in which the total photon budget is limited, the camera exposure time is fixed, and different aberrations or phase plates produce focal-plane intensity patterns with varying brightness. In this set of evaluations, we focus only on the effect of shot noise. The SNR of the brightest pixel varies with the aberration and ranges from 10 to 30 for the diffraction-limited beam. The results are shown in the following figure:

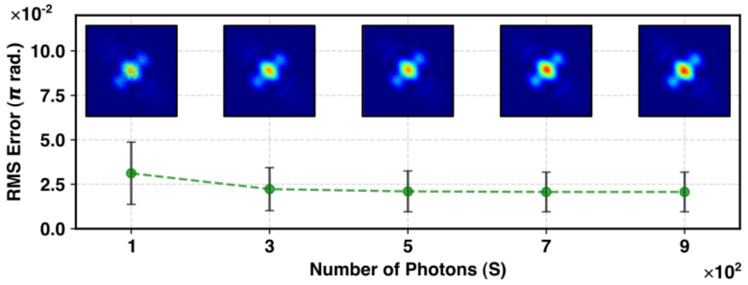

Figure 6. The performance of the model under low-light conditions. Evaluation has been done in the presence of photon shot noise. The number of photons indicates the average photons the brightest pixel a diffraction limited beam receives. The insets in the figure show a Gaussian beam aberrated with a positive value of oblique astigmatism.

These results indicate that the APN performs consistently well under low-light conditions, even when the captured intensity image is suboptimal.

Comments

In line 59 and in the supplement proof of focal plane ambiguity can you explain the conjugate inverse of phi? phi is a real-valued function and thus it's conjugate is just itself? Do you intend to say the conjugate of the reflected pupil function? If so, be specific because I think the claim that conj(phi(-x)) = -(phi_odd(-x) + phi_even(-x)) is not true

Response

We thank the reviewer for this remark and agree that the original phrasing could cause ambiguity for the reader. The conjugate inverse was intended to be defined for the field itself, not the phase. We have therefore revised the sentences in the text and the supplementary information to make the explanation clearer: The following are now added to the supplementary and the main paper respectively.

<u>Supplementary Information:</u>

Since the focal-plane intensity depends only on the magnitude of the Fourier transform, any phase function $\phi(x, y)$ and its sign-negated-reversed counterpart can produce identical intensity patterns. This introduces an ambiguity in which $\phi(x, y)$ and $-\phi(-x, -y)$ (the "ambiguous pair") yield the same focal-plane intensity, which we refer to as the Focal Plane Ambiguity (FPA). To illustrate this symmetry, we decompose $-\phi(-x, -y)$ into its even and odd components:

Main Text

Such phase ambiguity arises from a fundamental spatial symmetry in coherent imaging systems, whereby sign-negated-reversed phase distributions, $\phi(x, y)$ and $-\phi(-x, -y)$, produce identical intensity patterns at the image plane.

Comments

I may be mistaken but it seems like this condition of even symmetric bias is not a necessary condition. Any phase that has a non-zero even component (i.e., is not odd) should also accomplish the same outcome, no? The odd component won't affect anything but the even component should help disrupt the phase ambiguity. If this is true, you should be explicit about why you choose an even only component when any bias technically suffices.

Response

We thank the reviewer for highlighting this point. We agree that the phase for the bias does not need to be purely even. The necessary condition is that the phase must contain **a non-zero even symmetrical** component. Based on our experiment which is also briefly explained in this response, the presence of odd components does not contribute to resolving the ambiguity, and in some cases, as we observed, having an odd component may even slightly decrease the accuracy by introducing additional distortions. Therefore, while a purely even phase is not strictly necessary, it's the even component that is essential. We apologize if this was not clear before.

To investigate the role of odd components, as part of our original study, we evaluated bias configurations in which the bias was allowed to learn both even and odd components. Interestingly, in nearly all training runs, the learned bias spontaneously evolved into a predominantly even symmetrical pattern. This indicates that the optimization process naturally suppresses odd components. Below, we provide several examples where the bias converges into even symmetrical shapes:

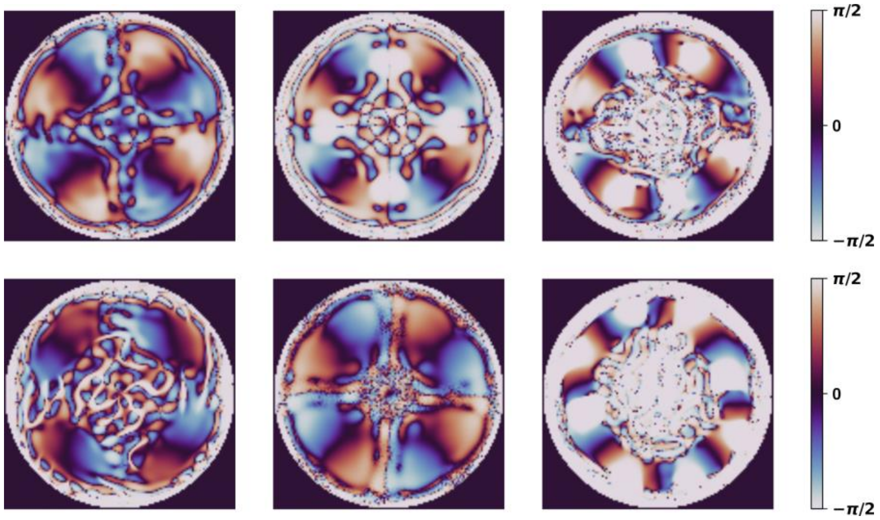

Figure 7. Multiple instances of the trained biases without symmetry constraints.

We conducted our final experiments using one arbitrary bias and two pure even symmetrical biases. One bias allowed learning from only a quarter of the data, while the other allowed learning from only one-eighth. Ultimately, the radially symmetric bias (one-eighth) demonstrated the most stable performance and the highest accuracy, as shown in the following figure. In addition, results from preliminary fabricated devices demonstrate that the proposed bias is significantly less sensitive to fabrication imperfections, providing a strong additional justification for its selection.

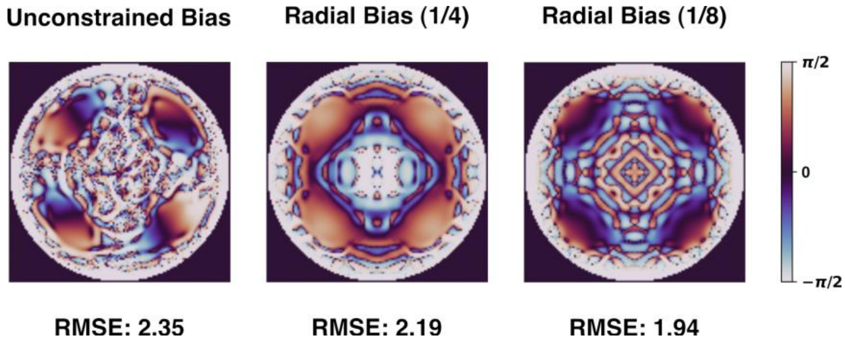

Figure 8. Trained biases with and without symmetry constraints (RSME 10^{-2} π rad.).

It is also worth mentioning that while any phase bias with a non-zero even symmetrical component should lift the focal-plane ambiguity, the specific choice of pattern is important in practical applications. For example, we have shown Z_{12}^{-10} as the bias in the following figure. This specific mode has a high spatial frequency and primarily induces changes outside the area of interest. In the following figure, the intensity response of the two ambiguous pairs of oblique astigmatism looks almost identical. Given this, one of our goals in learning the optimal bias is to

ensure that important information remains distinguishable and contained within a limited crop. We have revised the definition of bias in the manuscript and Supplementary Section 2.2.2. Additionally, we have added a subsection titled **"Odd Components"** (Section 2.2.3) to clarify this point further.

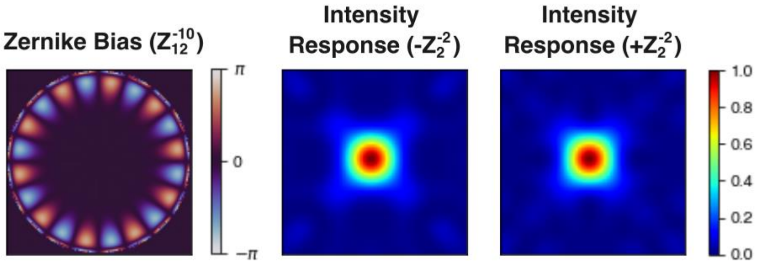

Figure 9. High-order Zernike polynomials as a bias, resulting in changes outside the cropped area.

Comments

On that note, what happens if you optimize an arbitrary bias (instead of a purely even one), will it do better because it has more degrees of freedom or worse because it is inefficient in the sense that only the even component contributes to FPA. One could argue that changing the odd component could still help with other forms of ambiguity that prevent total injectivity.

Response

We thank the reviewer for this comment and apologize for any apparent redundancy, given the close connection between these questions.

Based on our experiments, when an arbitrary bias is trained, it is highly likely that the optimization process will naturally suppress odd components. As a result, the pattern tends to evolve into a predominantly even symmetrical pattern (please refer to Figure 7). If the final pattern contains dominant odd components, these components will not impact the ambiguity removal. Our final experiments, which also involved training with an arbitrary bias, indicate that the accuracy achieved with these odd components is not as high as that of the purely even component. Specifically, the RMSE for the arbitrary bias was $2.35 \times 10^2 \pi$ radians, compared to $1.94 \times 10^2 \pi$ radians for the even component (please refer to Figure 8).

Comments

It would be nice to summarize the ambiguity correction into a single mathematical statement like "FPA is removed by any bias whose even component does not lie in the span of the finite set of even Zernike polynomials producible by the system" which you can prove quite easily. I think the discretization is nice for intuition but this statement I believe should hold in hilbert space as well.

Response

We appreciate the reviewer's constructive feedback. We agree that having a single clear mathematical statement to eliminate ambiguity is very helpful. In response to the reviewer's suggestion, we have added the following sentence to the manuscript:

"To lift the inherent ambiguity, we introduce a mathematical model that reveals a predominant requirement: the bias must have a non-zero even symmetrical component that lies outside the subspace spanned by the finite set of even Zernike polynomials producible by the system"

As well as an additional sentence in the Supplementary Information Section 2.2.2:

"Under this condition, any bias with a non-zero even component that lies outside the span of the system's producible even Zernike polynomials can resolve the focal-plane ambiguity."

Comments

Another small confusion: you say you imposed radial symmetry constraints and only learn a 1/8th wedge. Your function doesn't appear to be radially symmetric (i.e., invariant to rotation) unless I'm using that term incorrectly. Moreover, to achieve a purely even function isn't it sufficient to just learn a quarter? Why do you pick 1/8?

Response

We appreciate the reviewer's comment and apologize for any confusion. The radial symmetric term we use refers to a shape that has a repeating pattern when rotated by a certain degree (like a starfish), whereas circular symmetry describes shapes that remain unchanged under rotation. We have added further clarification in the Supplementary Information Section 3 to address this distinction. Yes, learning only a quarter of a phase plate can create a sufficient even bias. During our training trials, we explored these two types of radial biases: one where a quarter is learned (RSME of $2.19 \times 10^2\,\pi$ radians) and one where only one-eighth is learned (RMSE of

$1.94 \times 10^{2}\ \pi$ radians). We found that the bias we selected (one-eighth) demonstrated better performance and achieved a lower RMSE. This is the reason we chose this particular bias. Please refer to Figure 8 for the errors and phase profiles.

Comments

For the baseline biases, the zernike modes are nice because they validate that being in the span of the producible zernike modes fails to fix FPA. However it would be nice to also see a high order, even zernike mode beyond what the network can produce as another baseline bias. It satisfies the necessary condition to fix FPA and should have potentially less scattering than the random mask. I think have 3 lower order zernikes is redundant and fails to adequately capture reasonable baseline options.

Response

We thank the reviewer for this constructive suggestion and agree that having higher-order Zernike biases provide a more meaningful baseline. Following this suggestion, we conducted baseline tests using additional higher-order Zernike polynomials (**Z_{22}, Z_{23}, and Z_{37}**). For this comparison, the APN was trained using various fixed biases, while the biases themselves were not trained. Aberrations in the dataset were generated using the first nine Zernike polynomials (dataset with adjusted strengths). We note that the previous comparison was conducted using a dataset that included 14 polynomials. For a fair comparison, we have selected the dataset with 9 polynomials, the same number of polynomials our bias was trained on. To establish the baselines, we trained the model without any biases. with low-order Zernike modes **(Z_3 and Z_4)**, higher-order modes (**Z_{12}, Z_{22}, Z_{23}, and Z_{37}**), and two maximally random phase masks (regular and symmetric) as suggested by the reviewer. The following figure shows the validation curve from the training process.

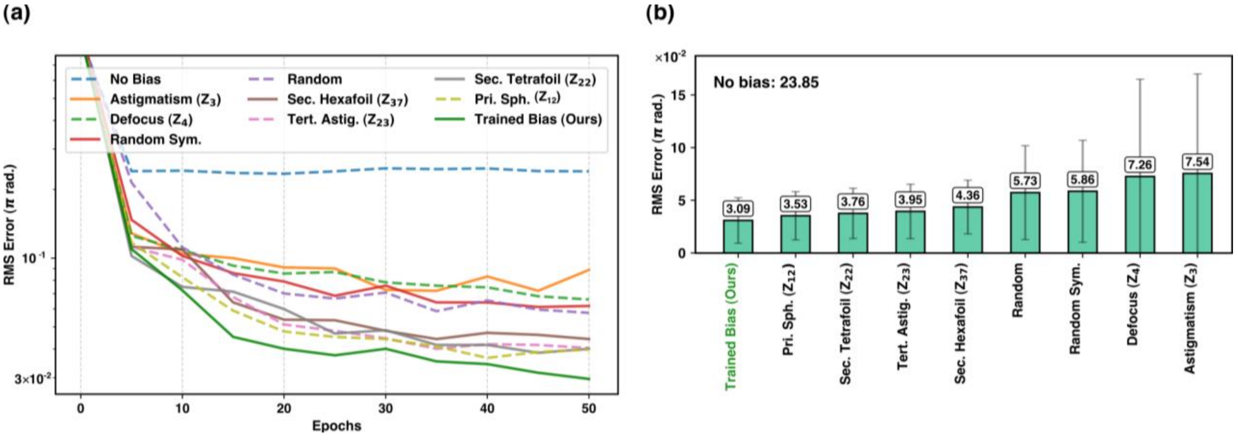

Figure 10. Comparison of the trained bias against other fixed biases. (a) the flow of the learning process and the corresponding validation errors, (b) the RMSE of the biases on the test set.

The results show that higher-order Zernike modes demonstrated improved performance compared to the lower modes and the random bias. This observation supports the theory discussed in Supplementary Information Section 2.2.2. Ultimately, the trained bias outperforms the other biases because it is specifically optimized to eliminate ambiguity, create distributable patterns, and account for crop properties such as resolution and frequency range, thus achieving the best results. Additionally, due to the complex, even symmetrical pattern of the trained bias, it is less likely to be canceled by higher-order aberrations compared to fixed biases.

To emphasize the effectiveness of each bias, we have also plotted the individual RMSE for each beam profile. This figure demonstrates that our trained bias outperforms all other biases in every setting. We have updated Section 6.1.1 of the Supplementary Information to include higher-order Zernike modes and have clarified the section on random masks.

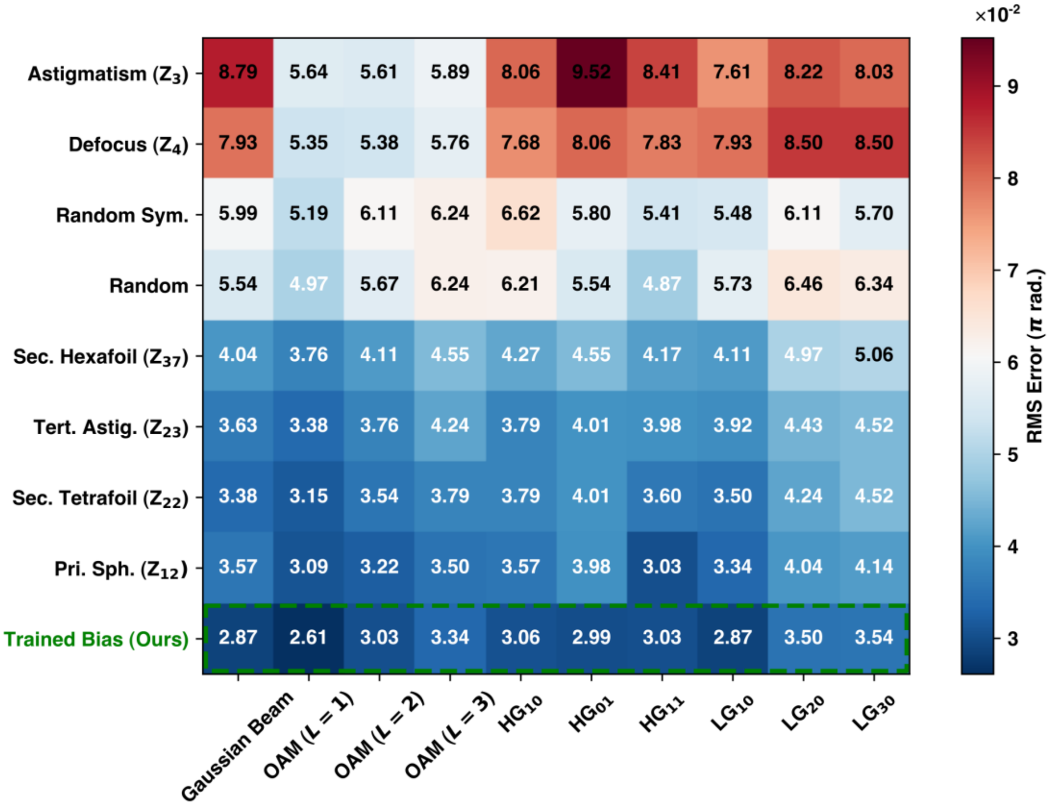

Figure 11. Individual RMSE values computed for each bias and each beam profile (The experiment with no bias was omitted for visualization purposes.)

Comments

I'm curious how you implemented the random bias baseline. I imagine how smooth the random profile is may have great effect on its performance. Less smooth likely causes aberrations to be more distinguishable but also scatters more. Indeed I believe a maximally random mask should work quite well because the resulting speckle will be extremely sensitive to small phase changes. What if you make an even random mask (i.e., randomize 1/4 of the mask and then reflect to create the rest).

Response

We thank the reviewer for this feedback, and apologize if this was not clear. Our random bias acts similarly to an optical diffuser. The phase shift for each pixel is uniformly distributed between 0 and 2π. As the reviewer indicated, this creates a maximally random mask that generates a speckle intensity pattern at the focal plane. Based on the reviewer suggestion, we have performed the baseline test with a symmetric random bias as well. The phase profiles of these biases are depicted in the following figure:

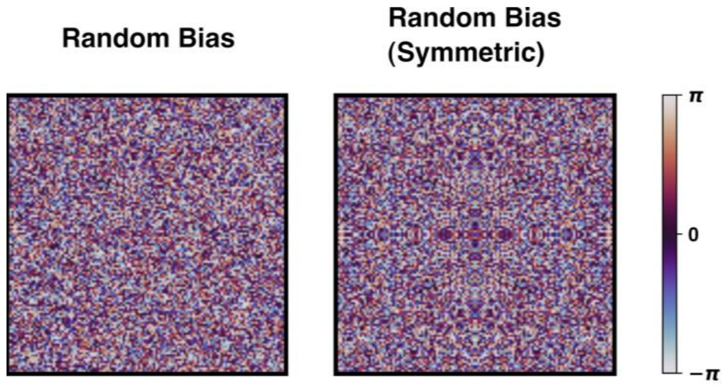

Figure 12. The phase profiles of random biases.

We compared the performance of the model under different bias schemes. Random phase profiles were expected to outperform low-order Zernike modes, as the latter can be partially canceled by the aberration itself. The results support this expectation. Specifically, the random biases achieved RMSE values of $5.73 \times 10^{-2}\pi$ radians and $5.86 \times 10^{-2}\pi$ radians for the symmetric case, compared with the baseline (no bias), which yielded an RMSE of $23.85 \times 10^{-2}\pi$radians. However, the random biases were less accurate than higher-order polynomial biases and the trained bias, which achieved a lower RMSE of $3.09 \times 10^{-2}\pi$ radians. (Please refer to Figure 10).

The random bias underperforms because the random phase creates a more complex and non-linear relationship between intensity and phase. This reduces the efficiency of interpolation and generalization performed by the neural network. Due to scattering, these biases spread valuable information over a larger area, making it more challenging for systems with limited crop sizes and

neural networks with low-resolution inputs. Furthermore, these random phase profiles are more susceptible to photon shot noise, leading to fewer photons being collected per unit area by the detector. That is the reason why we prefer a bias that keeps the light as focused as possible. To gain a better understanding of this, we conducted additional simulations and compared the trained bias to the random ones in the presence of photon shot noise. In this simulation, the brightest pixel in the diffraction-limited beam receives, on average, from 100 to 900 photons. The following figure demonstrates these results:

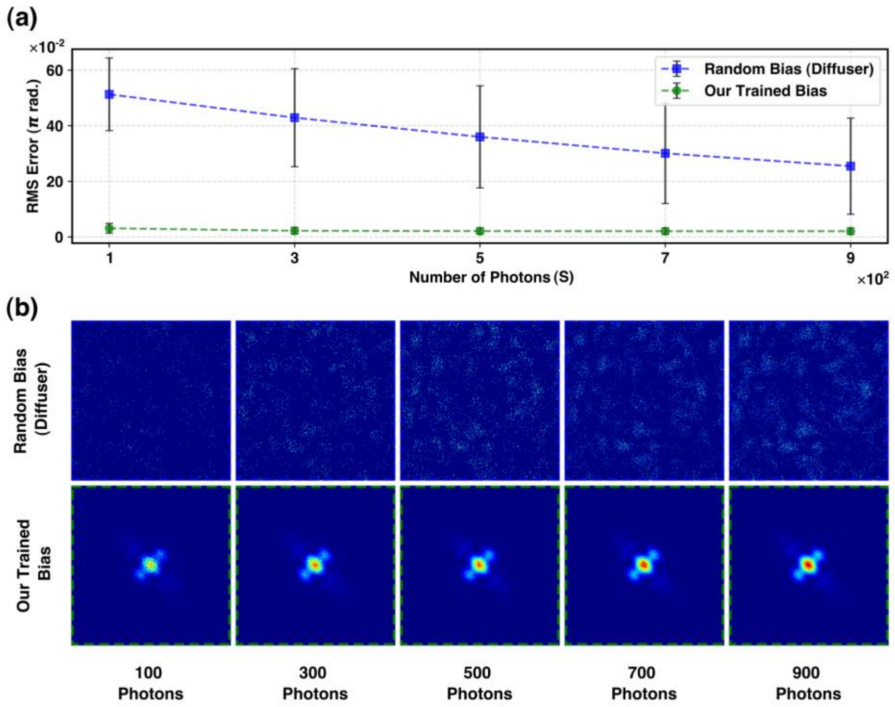

Figure 13. Comparison of trained bias and random bias in the presence of shot noise. (a) RMS error as a function of the number of photons, (b) intensity response of the biases to astigmatism aberration.

The intensity images (b) display the same aberrated beam, utilizing the trained bias and the random non-symmetric one. When using the random bias, the camera detects fewer photons, causing the pattern to be barely visible in low light conditions. As a result, the error in predicting the aberration is significant (RMSE of $25.47 \times 10^{-2}\pi$ radians, S=900). In contrast, the intensity pattern generated with the trained bias maintains its original shape, leading to a lower error margin (RSME of $2.07 \times 10^{-2}\pi$ radians, S=900). We have updated Section 6.1.1 of the Supplementary Information to clarify the random masks, and updated Section 6.5 to include the performance of the random mask in lowlight condition.

Comments

How many coefficients does the model predict during inference? Is this number fixed or adaptable (do you have to retrain the model to accommodate more or less coefficients? If so, did you retrain the model multiple times for different coefficient ranges?

This seems like a potential practical drawback and perhaps should be discussed

Response

We thank the reviewer for bringing up this insightful point. We trained two neural networks: one that predicts **9 coefficients** and the other predicts **14 coefficients** during the inference time. The first network was trained on a dataset in which aberrations were generated using the first nine Zernike polynomials. The second network was designed to demonstrate the model's capability to handle higher-order aberrations. Both networks were trained using the same bias settings. We apologize for any confusion; we have updated the methods section and Supplementary Information Section 3 for clarity. In the revised version of our work, we also included a network that can predict up to **27** Zernike coefficients (please refer to Supplementary Information Section 6.7).

Since the proposed method uses an indirect phase-prediction approach, the network is designed to output a fixed number of Zernike coefficients. We also trained the system using a direct method based on a U-Net architecture[16]. This network predicts the phase directly, rather than predicting the Zernike coefficients. However, this approach did not result in any improvements compared to the indirect method. The reason for this is that the training set is expressed in the Zernike domain, which limits the network's ability to perform better. Essentially, the network has the same expressive power as the dataset it was trained on.

Adding new categories to a neural network, such as new Zernike coefficients, typically requires retraining. However, some approaches involve less computational overhead, such as transfer learning[17], where the earlier layers of the network are fixed, and only a few output layers are trained on the new data with a lower learning rate. Despite these alternatives, the most accurate method is to retrain the entire network.

Although the number of coefficients is fixed, this does not present a practical limitation in our application, as retraining is fast enough to be feasible. For instance, training the network with 9 coefficients for 100 epochs takes approximately an hour and a half on our system (RTX 3080 TI). Even when trained for only 20 epochs, the network achieves decent accuracy, requiring around 20 minutes of training. The same holds true for the network with 14 coefficients; training it for 20 epochs also takes about an hour and a half. We appreciate the reviewer for highlighting this issue and acknowledge the potential drawbacks of neural networks. We have expanded the discussion section to address this concern more thoroughly.

Comments

To get a better sense of how much the model helps, could you simply run Gerchberg-Saxton or a gradient-based approach that directly regressing coefficients to best match the measured intensity through your learned bias? It doesn't need to be crazy comprehensive but I'm curious to see it on maybe a small subset of the experimental/simulated data. It would also give a sense of how well the bias removes ambiguity (and not just the model). If there is a strong reason not to do this, than feel free to bring it up.

Response

We appreciate the reviewer's insightful observation. It is indeed crucial to evaluate the effectiveness of the trained bias in eliminating the FPA when applied to other intensity-based approaches. Additionally, assessing the contribution of our neural network-based estimator to the overall accuracy of the system is important. Following reviewer's suggestion, we have conducted an extensive comparison on this topic and have added the relevant results section to Supplementary Section 7.3.3.

We have implemented the Gerchberg-Saxton (GS) and a Gradient Descent-Based (GDB) optimization method and compared them with our method, assessing both with and without the trained bias. The implemented GDB method uses the Adam optimizer and incorporates the Bluestein propagation method to get the intensity at the focal plane. The GS algorithm utilizes the Fast Fourier Transform (FFT) to propagate the beam at the focal plane. To achieve the same frequency range and scale as the Bluestein method, we needed to zero-pad the FFT to a larger size, which increases the computational complexity of the method. We conducted experiments using both the uniform and Kolmogorov datasets, which were collected experimentally. The following table displays the obtained average RMSE for each of the datasets, confirming that our proposed method outperforms both the GDB and the GS algorithm.

Distribution / Model	Uniform Distribution (RMSE π rad.)				Kolmogorov Distribution (RMSE π rad.)			
	Gaussian	OAM (l=1)	HG_{10}	Total	Gaussian	OAM (l=1)	HG_{10}	Total
**GS w/o Bias**	0.452 ± .120	0.403 ± 0.124	0.455 ± 0.122	**0.437±0.124**	0.295 ± 0.099	0.238 ± 0.083	0.323 ± 0.130	**0.285±0.111**
**GS w Bias**	0.460 ± 0.135	0.416 ± 0.127	0.462 ± 0.121	**0.446±0.130**	0.302 ± 0.114	0.243 ± 0.083	0.312 ± 0.123	**0.285±0.112**
**GDB w/o Bias**	0.333 ± 0.227	0.298 ± 0.235	0.365 ± 0.208	**0.332±0.225**	0.234 ± 0.198	0.118 ± 0.144	0.260 ± 0.182	**0.204±0.186**
**GDB w Bias**	0.167 ± 0.181	0.195 ± 0.229	0.251 ± 0.204	**0.204±0.209**	0.082 ± 0.149	0.081 ± 0.084	0.178 ± 0.139	**0.114±0.135**
**APN w/o Bias**	0.293 ± 0.127	0.125 ± 0.045	0.327 ± 0.125	**0.248±0.138**	0.224 ± 0.106	0.110 ± 0.042	0.277 ± 0.124	**0.204±0.120**
**(Our work) APN w Bias**	**0.074 ± 0.027**	**0.067 ± 0.023**	**0.110 ± 0.049**	**0.083±0.038**	**0.059 ± 0.017**	**0.057 ± 0.016**	**0.090± 0.033**	**0.069±0.028**

Table 1. Comparison of our approach to other single-shot intensity-based approaches using experimentally captured aberrated beam images.

By incorporating the trained bias, we have improved not only the neural network-based estimator **(66.5% reduction in RMSE)** but also the gradient-based one **(38.5% reduction in RMSE)**, demonstrating the effectiveness of the trained bias in enhancing the injectivity of the function. This result also indicates that once the bias is trained using our proposed approach, it can be utilized in other methods. Although this may lead to reduced performance, even if it may provide greater flexibility.

Both neural network-based and gradient-based models come with their own advantages and disadvantages. The neural network approach requires a large dataset for training and is typically less flexible than gradient-based methods. It may also display undesirable behavior if trained on a limited dataset or if the appropriate regularization techniques are not used. However, neural networks often deliver more accurate and faster predictions (~5ms per image), and they are generally more robust to noise.

On the other hand, gradient-based models offer greater flexibility and interpretability. However, they tend to be slower due to their iterative nature (~570ms per image) and are prone to getting stuck in local minima. Additionally, the choice of the initial point can significantly impact their performance. The obtained results confirm that the GDB underperforms compared to APN because of the mentioned issues. The GS algorithm did not show any improvements when incorporating the bias. It generally performed poorly in aberration detection because it lacks direct optimization and was designed to recover smooth, low-order, subtle aberrations. This makes it highly sensitive to noise and prone to stagnation. Based on feedback from the reviewer, this discussion has now been added to the discussion section of the manuscript.

Comments

I could argue that the classical approach could be more flexible and generalizable because you can always easily select exactly which coefficients you want to fit and you aren't worried about some unpredictable/uninterpretable model failure. Moreover, it is likely more agnostic to different imaging systems/parameters or explicitly incorporates them into the solution.

Response

We thank the reviewer for this valuable point. We agree with the reviewer that classical methods can offer greater flexibility and interpretability. However, there is an inherent trade-off between neural network-based approaches and classical search or optimization-based methods, and the choice depends on the specific application. Neural networks are typically single-shot, fast (~5ms per image), and capable of handling complex data with high accuracy. Notably, in our approach, the network does not require prior knowledge of the beam type. Additionally, the end-to-end training process allows the network to learn a trainable bias. The main limitations of this approach are the requirement for a large training dataset (on the order of 10^4 samples per beam profile), one-time training cost, and potential challenges in adaptability and interpretability.

Classical methods, such as gradient-based optimization, offer greater flexibility and interpretability and can explicitly incorporate system parameters. However, they tend to be slower due to their iterative nature (~570ms per image). These methods are also prone to getting stuck in local minima or saddle points and are highly sensitive to initial conditions, often necessitating multiple runs with different initializations to achieve convergence.

In practice, the choice depends on the system:

- If only a single aberration needs correction, or the system changes continuously, classical methods may be more suitable.
- If multiple aberrations must be corrected in a stable system, neural network-based approaches are advantageous due to their speed and accuracy.

In our study, we aimed to develop a single-shot system that is both fast and accurate. Following the suggestion by the reviewer, we evaluated the network using out-of-domain samples to verify its generalizability, and it demonstrated strong performance. We also compared our method to simple intensity-based approaches and found out that our approach outperformed them in terms of accuracy (66% reduction in RSME), which highlights the limitations of gradient-based optimization in reaching global minima. We have included a discussion on this topic in the discussion section.

Comments

The experimental calibration seems like an important step to get good results, can you talk about which steps were exclusively to ensure that you could properly measure error against your SLM-induced aberrations and which steps are critical to perform if you were to insert such a system into a practical imaging setup (e.g., adaptive optics).

Response

We thank the reviewer for this question, as calibration is indeed a critical step in our experiment. To ensure that the results are reproducible and aligned with our simulations, we first corrected the internal phase correction provided by the manufacturer (Santec). We ensured that the experimental system contained the fewest possible optical elements so that internal aberrations from the setup itself were minimized. The inherent aberrations were corrected using the model trained on theoretical data. The figure of merit here is the beam quality, which we compare directly against the ideal beam from theory. All optical elements were selected carefully based on the input wavelength, and coating specifications were chosen to avoid chromatic aberrations.

We tested all individual Zernike aberrations to confirm that our convention is consistent with the aberrations sent to the SLM. The amplitudes of the Zernike polynomials in the experiment match those used in simulation to make sure that the model outputs corrective Zernike amplitudes, which must scale proportionally when applied on the SLM. The beam size and the mapped Zernike phase size on the SLM were matched so that the beam experiences the full phase distribution. We used phase-profile scaling and a digital aperture to block unused SLM regions.

We applied a blazed grating on the SLM to spatially separate the modulated beam from the unmodulated components. The latter contain (i) the residual y-polarized light owing to the finite extinction ratio of the polarizer, and (ii) the unmodulated fraction of the x-polarized beam. We then ensured that the image is correctly formed at the focal plane of the imaging lens. Here, the ambiguous Zernike pair was used as a diagnostic, as both signs of the ambiguous pair should produce identical intensity images.

Following the reviewer comments, we have added the following text in the methods section:

SLM Calibration and Aberration Control

To ensure consistency between simulation and experiment, the SLM was first corrected using the manufacturer-provided internal phase calibration. The optical path was kept intentionally minimal to reduce system-induced aberrations. Any residual distortions were then compensated using our model trained on theoretical data, with beam-quality agreement to the ideal profile serving as the figure of merit. All optical components used were selected and coated for the

operating wavelength to avoid chromatic effects. We validated the phase response of the SLM by applying individual Zernike modes (Z_1-Z_9) and confirming that (i) our sign and amplitude conventions matched those used in simulation and (ii) the applied Zernike amplitudes scaled linearly when written to the SLM. The beam diameter was matched to the mapped Zernike phase profile using digital apertures to remove unused SLM regions. A blazed grating was added to spatially separate the modulated beam from residual unmodulated components arising from finite polarizer extinction. Finally, the image plane was verified at the focal plane of the collection lens, where the ambiguous Zernike pair served as a diagnostic since both signs must yield identical focal-plane intensity patterns.

Comments

In this discussion can you also speak more to the wavelength specific calibration you did to ensure broadband performance? Would it be challenging to do this calibration in a real adaptive optics context?

Response

We thank the reviewer for this question. The wavelength-specific calibration we performed primarily involved adjusting the blazed-grating angle for each operating wavelength. A blazed grating works by directing most of the optical power into the "blazed" diffraction order, which corresponds to a specific angle. This angle depends on the wavelength, so changing the wavelength alters both the diffraction efficiency and the diffraction angle. Therefore, for each wavelength, we updated the blaze angle to ensure that the maximum power remained in the blazed order and that the diffracted beam followed the user-defined optical path. This is important for keeping internal aberrations minimal, particularly those corresponding to Z_1 and Z_2, which represent x- and y-tilts.

We also ensured that the imaging lens used in the experiment had an AR coating suitable for the chosen wavelength and that it was achromatic. Apart from these adjustments, no additional wavelength-specific calibration was required in our experiment.

Comments

In fig s21 is there a reason the 3rd zernike coefficient consistently has the highest error?

Response

We thank the reviewer for highlighting this crucial point. It is indeed valuable to understand why the third Zernike coefficient (Z_3) consistently exhibits a slightly higher error in Figure S21. After careful consideration, we found that the slightly higher error (approximately 0.01 in Zernike space) was due to transient fluctuations near the end of the training. This occurred because of the relatively high learning rate during that stage and the stochastic nature of minibatch optimization. The variable Z_3 functioned as a trade-off to minimize the error on the training batch, which resulted in slightly larger errors in that same batch.

To address this issue, we implemented a learning rate scheduling strategy that decreases the learning rate during the final epochs. This change resulted in more stable and consistent predictions across all modes. Please note that the bias phase profile is unaffected, as it is trained for only the first 20 epochs and remains fixed to avoid saturation. We have updated Figure S5, S27 and S28, and the training section to reflect these improvements, and we appreciate the reviewer's constructive feedback.

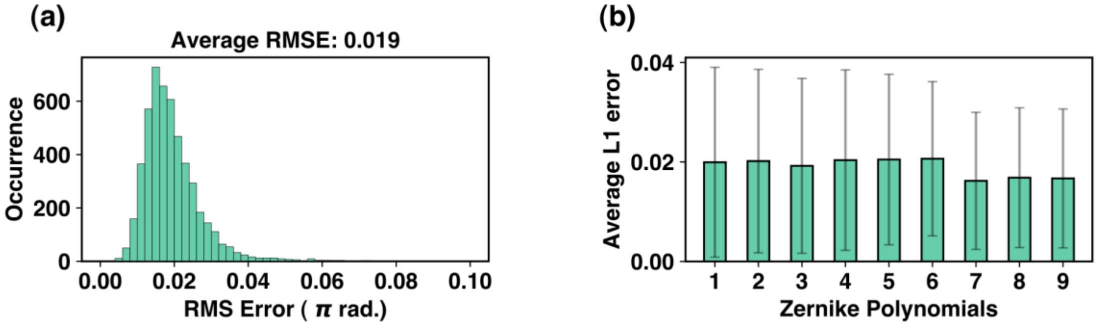

Figure 14. (a) error distribution on the test set, (b) average L1 error for each Zernike polynomial on the test set.

Comments

Is there a reason why you think the error on the held-out Kolmogorov coefficients is lower than on the uniform set (which you trained on)?

Response

We would like to thank the reviewer for her/his comment. The reason is closely related to what the reviewer mentioned, but we want to emphasize a subtle distinction and provide clarification here. The error measured in the Kolmogorov dataset is generally lower than that in the uniform dataset, because the overall aberration strength of the Kolmogorov samples is slightly less than that of the uniform distribution. However, the lower error is attributed to the total phase RMS

rather than the complexity of each mode. When the model was evaluated across all modes (please refer to Figure S13.b), it produced consistent results across all 14 Zernike modes. We have included this clarification in both the main manuscript and the Supplementary Information Section 6.3 to ensure this distinction is clear.

Comments

Is it because the Kolmogorov set tends to feature 'easier' modes biased more toward lower orders?

Response

In the Kolmogorov instances, the magnitude of each coefficient decreases significantly as we move to higher-order polynomials (please refer to Supplementary Table 2). Higher-order coefficients contribute less to the total phase aberration compared to a uniform distribution, where each coefficient has an equal contribution. This results in lower phase strength (the Kolmogorov set has lower average RMS strength compared to the uniform set, as illustrated in Figure S4). As a result, the error measured in the Kolmogorov dataset is typically lower than that in the uniform dataset.

Comments

How would the model perform on more unstructured phase (e.g., smoothed random phase) not generated directly from low order zernike polynomials? Would it naturally find the best fitting coefficients or explode? Exploring this potential failure mode is of interest.

Response

We thank the reviewer for bringing this important topic about generalizability to our attention. Based on this comment and the other valuable feedback we received regarding the behavior of the model on higher-order Zernike aberrations, we conducted extensive evaluations of our model with these out-of-domain samples and added a section titled **"Out-of-Domain Evaluations"** to the Supplementary Information Section 6.6

In this set of evaluations, we generated three types of samples from different domains: atmospheric turbulence not directly produced from Zernike polynomials[18], smooth random phase distributions, and higher-order Zernike aberrations. The higher-order Zernike evaluations are discussed in the next comment. For each aberration type, we evaluated performance under two levels of complexity. For the turbulence model, we selected two Strehl ratios (0.5 and 0.2),

representing less and more severe aberrations. For the smooth random phase, we chose two different spatial resolutions of 8 and 15. For each complexity level, we evaluated the model on 5000 random instances. Multiple examples of the detection process on turbulence samples are shown in the figure below:

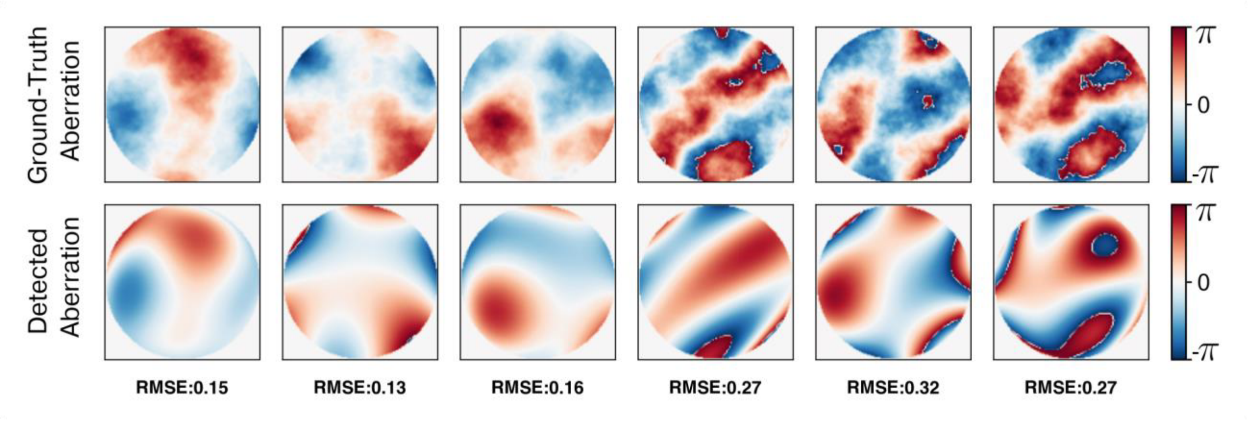

Figure 15. The first three instances on the left are generated with a Strehl ratio of 0.5, while the last three instances are generated with a Strehl ratio of 0.2. The error is represented in phase space using π radians.

The model effectively estimated the dominant low-order aberrations for both levels of turbulence severity, which confirms its robustness in dealing with atmospheric turbulence. The average RMSE for this experiment is 0.14 π radians for a Strehl ratio of 0.5 and 0.31 π radians for a Strehl ratio of 0.2.

Below, we also present examples for smooth random phases.

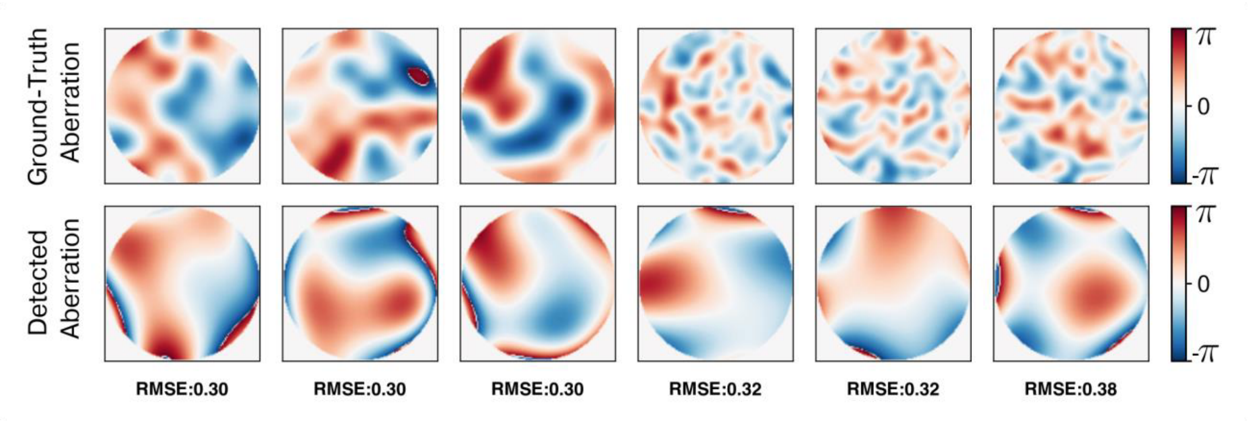

Figure 16. The first three instances on the left are generated with a spatial resolution of 8, while the last three instances are generated with a spatial resolution of 15. The error is represented in phase space using π radians.

For random phase patterns with relatively smooth spatial variations, the model identified the closest match within its representational capability. For random phases with more rapid spatial fluctuations, the model attempted to find the best match; however, due to the increased complexity, it primarily captured the center of mass of the phase distribution. The average RMSE is 0.38 π radians for a spatial resolution of 8 and 0.36 π radians for a spatial resolution of 15.

These results confirm that the proposed network shows good generalizability for out-of-domain instances. They also indicate that the network has not over-fitted to the training domain and has successfully learned the key features in the intensity images that are essential for predicting the phase.

Comments

I noticed you did a robustness test of zernike modes up to 55 but it's not clear to me if you actually tested the accuracy of predicting/correcting such modes with your model. If you didn't do this then explain why not.

Response

We thank the reviewer for bringing up this insightful point. We did not explicitly train and evaluate the prediction accuracy of the network for aberration spaces up to 55 Zernike modes. Instead, we performed a robustness test to assess whether the FPA is eliminated in higher-order aberration spaces by examining the intensity profiles of ambiguous phase pairs.

We used the LPIPS metric to quantitatively measure the perceptual differences between a given phase and its corresponding ambiguous pair. In the absence of bias, such phase pairs produce identical intensity profiles, resulting in a one-to-many inverse mapping. This test therefore directly examines whether the learned bias renders previously ambiguous phases distinguishable, thereby improving injectivity without requiring explicit network training.

This approach is computationally efficient and well-suited for high-dimensional aberration spaces (e.g., 55 Zernike modes), where direct training would require prohibitively large datasets due to the curse of dimensionality. Nevertheless, we did perform full training and evaluation for Zernike modes up to the 27th order (6th radial order), and these results are reported in Section 6.7. Based on the reviewer's suggestion, we have clarified Section 6.1.3, with the changes.

Comments

I would prefer you do the higher-order experiment and report the outcome. Given that the model appears to have a restricted set of coefficients it can learn, it's important to understand it's behavior for potentially higher modes (beyond what it's trained on) which could easily arise in an experimental setting. Even a few examples of what the model does when faced with something outside of its representational capabilities would be illuminating.

Response

We thank the reviewer for raising this important concern regarding the generalizability of the model. Based on the valuable feedback we received regarding the model's behavior on out-of-domain samples, including higher-order Zernike aberrations and unstructured random phase patterns, we conducted extensive additional evaluations and added a new section titled **"Out-of-Domain Evaluations"** to the Supplementary Information Section 6.6. For the higher-order Zernike aberrations, we generated aberrations using the first 35 Zernike coefficients (7th radial order). We evaluated performance under two different levels of complexity: (i) coefficient sets with equal contributions across the modes, and (ii) coefficient sets with more dominant lower-order modes. For each complexity level, we evaluated the model on 5,000 random instances. Representative examples are shown in the figure below:

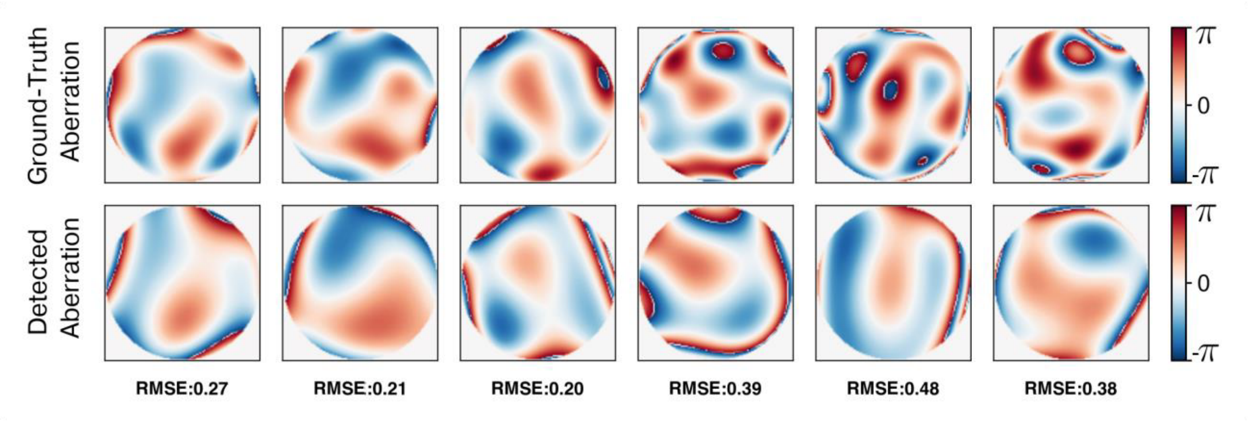

Figure 17. The first three instances on the left correspond to aberrations dominated by lower-order modes, while the last three instances correspond to aberrations with coefficients evenly distributed across all 35 modes. The phase error is represented in units of π radians.

As the results indicate, the model effectively estimated the dominant low-order aberrations in both complexity regimes and identified the closest match within its representational capacity. The predicted phase maps closely matched the lower-order components of the generated aberrations without collapsing. The average RMSE for instances with evenly distributed coefficients is 0.68 π radians, while for those with dominant lower-order modes it is 0.26 π

radians. These results confirm that the proposed network shows good generalizability to aberrations outside the distribution used in the training set. The discussion section has been updated to include this topic as well.

Comments

I don't have much experience with metasurfaces and cannot adequately evaluate that part of the manuscript.

Response

We appreciate the reviewer for their fairness in the statement. All required data in both simulations and experiments are included in the main paper or the supplement.

Comments

I noticed you only did the experiment at 600nm with the metasurface; is a potential limitation of using such a mask that it is not achromatic? If so, can you mention this, as it softens the claim of the system being 'broadband'.

Response

We thank the reviewer for this helpful feedback. The metasurface we designed is broadband, with the highest efficiency occurring at 600 nm. Geometric metasurfaces, which rely on both geometric phase and orientation, are generally broadband because their operation depends on the local phase gradient at the interface where light interacts with the substrate, which in our case is glass. To shape this phase, we orient and arrange the metasurface elements, each defined by a unit cell known as a meta-atom. In our device, the meta-atoms are fabricated from TiO_2 because TiO_2 exhibits very low optical loss in the visible wavelengths used in our experiment.

As with any optical element, the refractive index dispersion of the chosen material determines its effective bandwidth, and therefore our device also has a finite operational bandwidth. If we were to operate at other wavelength ranges, such as the infrared, we could instead use Si meta-atoms, which have low loss at those wavelengths. This would allow the metasurface to operate in the mid-IR or IR range depending on the design. Moreover, because Si has a higher refractive index, the meta-atoms could be made smaller, as the effective wavelength inside the material decreases.

In summary, our choice of material, which is primarily dictated by minimizing optical loss, determined both the fabrication constraints and the resulting bandwidth. Since our goal in this work was to operate efficiently in the visible regime, TiO_2 was the most suitable and well-studied option. However, the same design can be scaled to higher wavelengths using either TiO_2 or alternative materials that exhibit lower loss in the target spectral band.

Comments

One concern I have is how adaptable your model would be if I were to just place it into a new system with different imaging optics and parameters (NA, power, etc). Would you have retrain the model? Use a different bias? If so this provides additional overhead for a user and requires them to sufficiently understand their system and be able to calibrate it such that training data matches experimental data. I would love for a discussion on this potential limitation and how you envision your system being deployed as a practical wavefront sensor.

Response

We thank the reviewer for this thoughtful and important question. It is indeed pertinent, as our goal is for the system to remain adaptable across different imaging configurations, each of which may have its own parameters, constraints, and complexities. Since our primary objective is to ensure that the beam reaching the sample is as pure and aberration-free as possible, the only requirement is that the user is able to capture the focal-plane image. Regardless of the illumination power, numerical aperture, or other specifics of the imaging system, we can recover the phase of the optical system from that focal-plane measurement. No additional training data or special calibration is required beyond the standard SLM calibration discussed earlier.

We also appreciate the reviewer's request to elaborate on how the wavefront sensor can be deployed in practical applications. In the supplementary, we have added the section below regarding the system that can be inserted to measure wavefront aberrations in various cases, we have also mentioned this topic in the discussion section.

Inserting aberration correction system into a practical optical setup

To integrate this system into a practical configuration, only minimal changes are needed. The primary modification is the entry point of the laser into the imaging path. The key idea is to guarantee that the beam illuminating the specimen (for example, biological tissue) is itself not aberrated.

A relevant example comes from STED microscopy, where donut-shaped beams have been widely used. In many implementations, by the time the donut beam reaches the specimen, the optical

system has already introduced aberrations. Our approach ensures that the beam reaching the specimen is pre-corrected. Experimentally, this is achieved by taking a portion of the focal-plane image through an unequal beam splitter (e.g., R:T = 8:92), feeding it to the SLM for pre-compensation, and ensuring that the beam transmitted toward the specimen is corrected.

To guarantee that the beam remains pure, we collect approximately 8% of the beam using an unequal beam splitter and send it into our aberration-correction system. This system consists of a focusing lens and the metasurface used to lift the FPA ambiguity. The captured intensity image on the camera is then used to infer the aberration, and the corresponding conjugate phase is applied by the SLM. In this way, the SLM pre-compensates the optical system for the aberrations that would otherwise distort the OAM mode before it reaches the DUT.

This pre-compensation ensures that a clean, well-defined OAM beam interacts with the sample, allowing us to quantify the effects of both the sign and magnitude of the OAM mode on the DUT. In practice, the procedure requires only a single intensity image, and the aberration-correction algorithm supplies the necessary corrective phase. As a result, we can observe both the corrected beam (for aberration detection) and the aberration-free beam (for evaluating the DUT response) on the camera.

Concluding remarks of authors

We would like to thank the reviewer again for the exceptional quality of their questions. We hope our responses have fully addressed all expressed concerns. The referee comments have helped us significantly to improve the quality of the paper and its presentation. We are happy to address any further questions and implement any modifications the reviewers suggest.

References

1. Allen, L., Beijersbergen, M. W., Spreeuw, R. J. C. & Woerdman, J. P. Orbital angular momentum of light and the transformation of Laguerre-Gaussian laser modes. *Phys. Rev. A* **45**, 8185–8189 (1992).
2. Padgett, M. & Bowman, R. Tweezers with a twist. *Nat. Photonics* **5**, 343–348 (2011).
3. Hell, S. W. & Wichmann, J. Breaking the diffraction resolution limit by stimulated emission: stimulated-emission-depletion fluorescence microscopy. *Opt. Lett.* **19**, 780 (1994).
4. Wang, J. *et al.* Terabit free-space data transmission employing orbital angular momentum multiplexing. *Nat. Photonics* **6**, 488–496 (2012).
5. Fickler, R. *et al.* Quantum Entanglement of High Angular Momenta. *Science* **338**, 640–643 (2012).
6. Rittweger, E., Han, K. Y., Irvine, S. E., Eggeling, C. & Hell, S. W. STED microscopy reveals crystal colour centres with nanometric resolution. *Nat. Photonics* **3**, 144–147 (2009).
7. Barreiro, J. T., Wei, T.-C. & Kwiat, P. G. Beating the channel capacity limit for linear photonic superdense coding. *Nat. Phys.* **4**, 282–286 (2008).
8. Leach, J. *et al.* Violation of a Bell inequality in two-dimensional orbital angular momentum state-spaces. *Opt. Express* **17**, 8287 (2009).
9. Jack, B. *et al.* Entanglement of arbitrary superpositions of modes within two-dimensional orbital angular momentum state spaces. *Phys. Rev. A* **81**, 043844 (2010).
10. Malik, M. *et al.* Multi-photon entanglement in high dimensions. *Nat. Photonics* **10**, 248–252 (2016).
11. Kong, L.-J. *et al.* Complete measurement and multiplexing of orbital angular momentum Bell states. *Phys. Rev. A* **100**, 023822 (2019).
12. Ru, S. *et al.* Quantum state transfer between two photons with polarization and orbital angular momentum via quantum teleportation technology. *Phys. Rev. A* **103**, 052404 (2021).
13. Swartzlander, Jr., G. A. *et al.* Astronomical demonstration of an optical vortex coronagraph. *Opt. Express* **16**, 10200 (2008).

14. Zhao, S. *et al.* Measuring the complex spectrum of orbital angular momentum and radial index with a single-pixel detector. *Opt. Lett.* **45**, 5990 (2020).

15. Cubalchini, R. Modal wave-front estimation from phase derivative measurements. *J. Opt. Soc. Am.* **69**, 972 (1979).

16. Ronneberger, O., Fischer, P. & Brox, T. U-Net: Convolutional Networks for Biomedical Image Segmentation. in *Medical Image Computing and Computer-Assisted Intervention – MICCAI 2015* (eds. Navab, N., Hornegger, J., Wells, W. M. & Frangi, A. F.) vol. 9351 234–241 (Springer International Publishing, Cham, 2015).

17. Yosinski, J., Clune, J., Bengio, Y. & Lipson, H. How transferable are features in deep neural networks? in *Proceedings of the 28th International Conference on Neural Information Processing Systems - Volume 2* 3320–3328 (MIT Press, Cambridge, MA, USA, 2014).

18. Rosales-Guzmán, C. *How to Shape Light with Spatial Light Modulators*. (Society of Photo-Optical Instrumentation Engineers (SPIE), Bellingham, 2017).

Reviewer #2 (Remarks to the Authors):

The authors adequately addressed my concerns and added compelling new evidence to support their claims. I commend the authors for their receptive and comprehensive response. I support publication of this paper.

Response of authors

We sincerely thank the reviewer for their thorough evaluation of our work. We are pleased that our responses have addressed the concerns raised. We greatly appreciate the reviewer's constructive guidance throughout the peer-review process and their recommendation for publication.

Reviewer #2 (Remarks on code availability):

The code seems well organized and documented, and has adequate instructions to theoretically recreate results. That being said I do feel the authors should include a minimal working example that can be run with very little setup and timing. A suggestion is to include a pretrained model and small evaluation dataset with a jupyter/colab notebook that executes an experiment from the paper. As of now, the code that was provided doesn't appear to have a pretrained model nor a simple already-set-up script that can be executed.

Response of authors

We thank the reviewer for this constructive suggestion. In response, we have now created a lightweight Jupyter-notebook demonstration that handles dataset generation, model training, and evaluation. For immediate verification, we have also provided a small-scale dataset and the corresponding pre-trained model. The data and code are temporarily restricted due to an ongoing patent but have been shared with editors and will be publicly released upon patent publication. In the meantime, they are available from the corresponding author upon reasonable request.

Concluding remarks of authors

We would like to thank the reviewers once again for their time and the extensive, insightful feedback on our paper. The reviewer’s comments have been invaluable in helping us improve both the content and presentation of the manuscript. We greatly appreciate their careful consideration and thoughtful suggestions.
